# [*d*]-Carbon–carbon double bond engineering in diazaphosphepines: a pathway to modulate the chemical and electronic structures of heteropines†

**DOI:** 10.1039/c6sc00519e

**Published:** 2016-03-15

**Authors:** Yi Ren, Melda Sezen, Fang Guo, Frieder Jäkle, Yueh-Lin Loo

**Affiliations:** a Department of Chemical and Biological Engineering, Princeton University NJ 08544 USA lloo@princeton.edu; b Department of Chemistry, Rutgers University Newark NJ 07102 USA

## Abstract

We have designed and synthesized the first examples of 7-membered diazaphosphepines using phosphorus–amine (P–N) chemistry. Different from previous functional protocols of heteropines, the installation of π-conjugated substituents having diverse chemistries at the [*d*]-C=C double bond position of heteropine core allows us to effectively control the chemical and electronic structures in both the ground and excited states of these diazaphosphepines. This functionalization has led to a diverse set of crystal structures, which has in turn provided access to rich photophysical and redox properties. Of particular interest is the evidence for planar π-conjugated backbone in our non-aromatic heteropine and twisted intramolecular charge transfer, which have never been reported for heteropines. The introduction of electron-accepting substituents at [*d*]-position of diazaphosphepines results in heteropines that are more electron deficient than any heteropine reported to-date. As proof of concept, we have fabricated organic solar cells with heteropines as non-fullerene acceptors.

## Abstract 2

We report the first examples of 7-membered diazaphosphepines using phosphorus–amine (P–N) chemistry.

## Graphical abstract

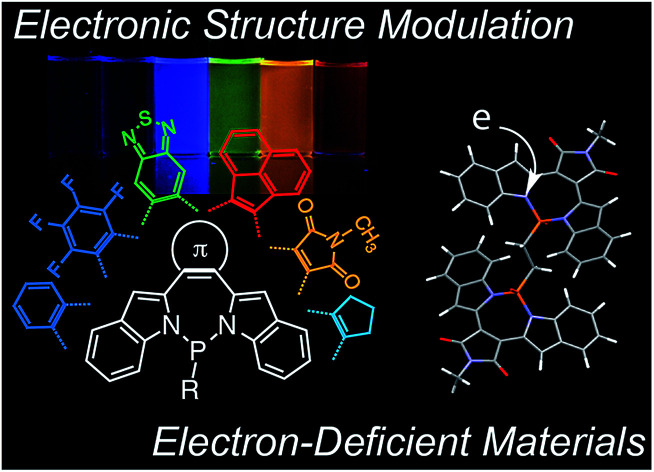

## Introduction

π-Conjugated cyclic building blocks that contain heteroatoms (B, N, O, Si, P, and S) have attracted a lot of attention due to their diverse chemical and electronic structures, and tunable electronic properties.^1–21^ Among these cyclic building blocks, five-membered heteroatom-containing cyclopentadienes, such as aromatic thiophene, furan, and pyrrole, are commonly installed in molecular and polymer semiconductors for light-emitting and light-harvesting applications.^21,22^ Anti-aromatic borole^23–26^ and non-aromatic phosphole^11–15^ have also become increasingly popular building blocks as they impart unique photophysical and redox character compared to their aromatic counterparts; derivatives containing these heteroatom-containing five-membered cyclopentadienes exhibit low bandgaps, strong electron-accepting capability and intramolecular charge transfer. By the same token, replacing the C=C double bond in the classical six-membered benzenoid building block with isoelectronic B–N fragments has generated azaborine and borazine; materials comprising these substitutions display absorption and emission at energies lower than their pure carbon counterparts and, as such, have been incorporated in optoelectronic devices.^27–32^

Heteroatom-containing seven-membered cycloheptatrienes, namely heteropines, have not been studied extensively. Previous studies have focused on their fundamental characteristics, including their conformational change, chemical reactivity and electronic structure.^33–40^ With the exception of Group 13-element-containing aromatic derivatives,^41–52^ these heteropines generally adopt “boat-like” structures and constantly undergo conformational inversion in solution, severely limiting backbone π-conjugation.^52–56^

**Chart 1:**
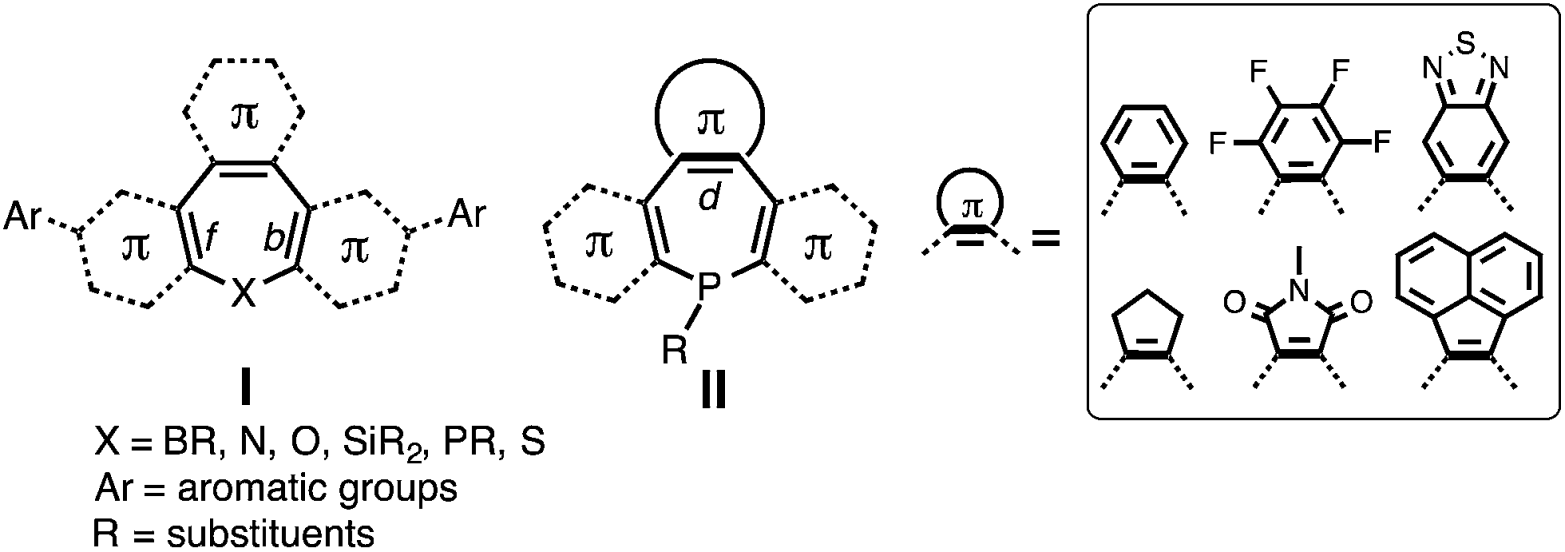
The chemical structures of heteropines in previous studies (I) and diazaphosphepines in current study (II).

Further exploration of heteropines for optoelectronic applications has thus far been hindered by significant synthetic challenges. “Naked” heteropines are unstable at elevated temperatures; some have even been reported to degrade at room temperature.^33–40^ As such, annulation of the [*b*,*d*,*f*]-C=C double bonds is required to stabilize the seven-membered ring (I in Chart 1).^33^ But the synthesis of annulated heteropines is not trivial given that these reactions involve intermediates that are difficult to handle and the synthetic protocols are limited to specific functional groups.^33–40^ Consequently, post-functionalization protocols that introduce aromatic substituents to the [*b*,*f*]-annulated heteropine core (*e.g.*, the introduction of Ar on I in Chart 1) have been the only synthetic pathway for tuning their chemical, electronic, and optoelectronic properties.^41–47^

Less known compared with other heteropines are the annulated P-analogues, particularly in the context of their optoelectronic properties.^11^ Given the Lewis basic character of their P-center, post functionalization should allow us to further fine-tune the properties of these derivatives. In this contribution, we report the design, synthesis and characterization of the first examples of seven-membered P-containing derivatives, namely diazaphosphepines, using mild phosphorus–amine (P–N) chemistry. Different from previous post-functionalization protocols, we have directly installed π-conjugated substituents at the [*d*]-C=C double bond position of the heteropine core (II in Chart 1) to modulate their chemical and electronic properties in both ground and excited states. Introducing electron-withdrawing substituents at the [*d*]-position of the heteropine core while simultaneously reducing sterics on the P-center has resulted in highly electron-deficient diazaphosphepines that are viable non-fullerene acceptors in organic solar cells.

## Results and discussion

### Synthetic design of diazaphosphepines *via* P–N chemistry

**Scheme 1:**
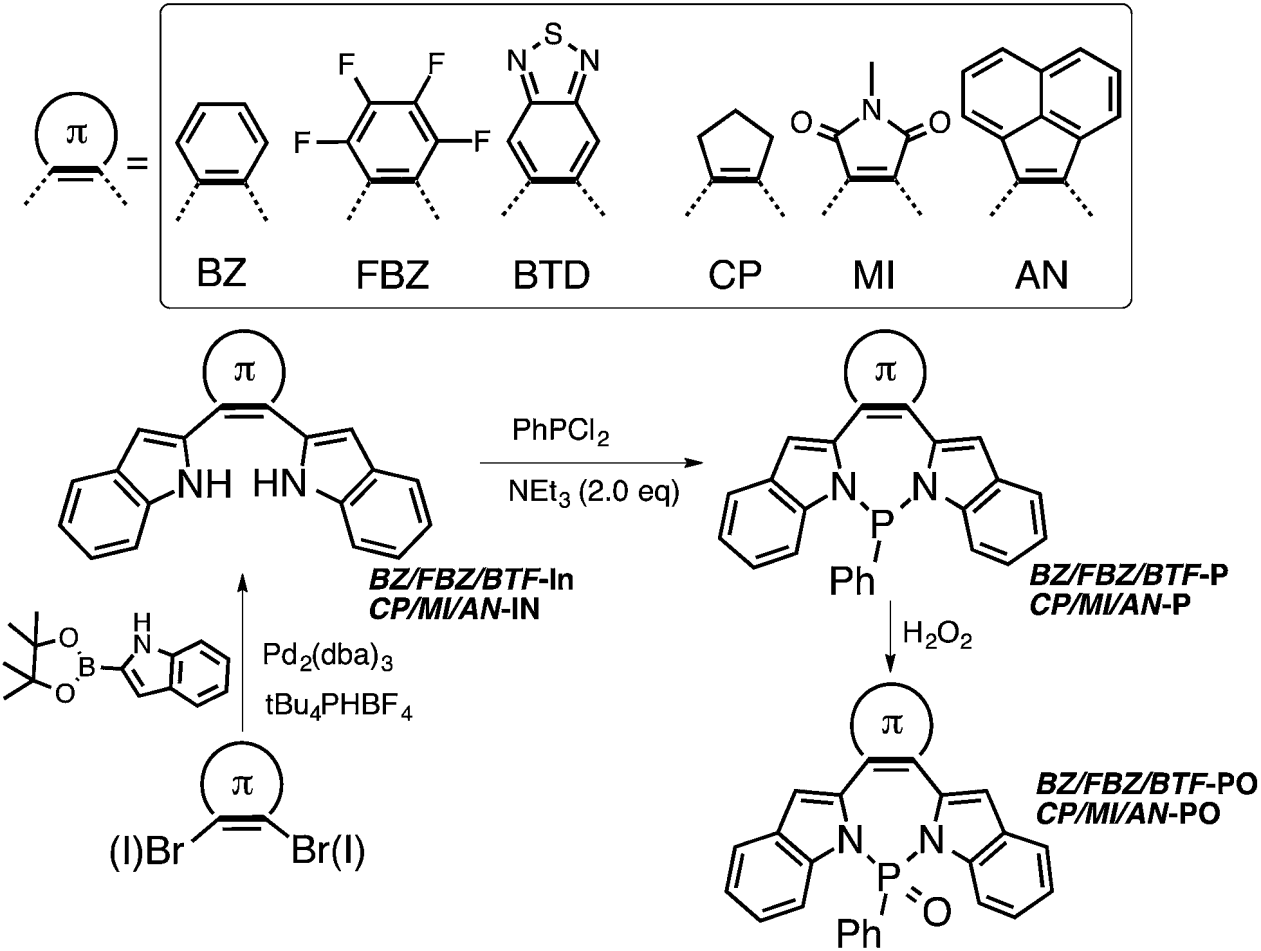
Synthesis of diazaphosphepines in this study.

We have chosen to introduce six π-conjugated substituents (Scheme 1), including aromatic benzene (BZ), perfluorobenzene (FBZ), and benzothiadiazole (BTD), and non-aromatic cyclopentene (CP), maleimide (MI), acenaphthylene (AN)^57,58^ at the [*d*]-position of the diazaphosphepine core. Due to differences in their aromaticity, BZ, FBZ, and BTD substituents should exhibit stronger confinement of the π-electrons compared to CP, MI and AN substituents. When installed at the [*d*]-position of the diazaphosphepine core, this difference in electronic confinement should impact the character of the resulting C=C bond accordingly. We thus expect BZ, FBZ, and BTD substituents to weaken electron delocalization in BZ-P, FBZ-P, and BTD-P, while CP, MI, and AN substituents to enhance electron delocalization in CP-P, MI-P, and AN-P. Electronic structure aside, we expect sterics to play an important role as well. Compared to five-membered ring substituents, like CP and MI, six-membered ring substituents, like BZ, FBZ, and BTD, as well as naphthalene-functionalized AN should exhibit stronger steric hindrance that will induce more twist when installed at the [*d*]-position of the diazaphosphepine core. This steric effect can also influence electron delocalization. The combination of both offers a powerful handle to fine-tune the intramolecular electronic communication of diazaphosphepines.

The synthesis of diazaphosphepines is summarized in Scheme 1. The syntheses of precursors BZ-, FBZ-, BTD-, CP-, MI- and AN*-*In are described in ESI.† Formation of the seven-membered ring can be readily achieved by refluxing BZ-, FBZ-, BTD-, CP-, MI- and AN*-*In with PhPCl_2_ in the presence of NEt_3_ in anhydrous acetonitrile solution, resulting in BZ-, FBZ-, BTD-, CP-, MI- and N*–*P in 55–80% yields. While the compounds were all obtained at reflux conditions, the ring-closing reaction to yield BTD-P and MI-P proceeds readily even at 0 °C, an indication of the ease with which these compounds can be made. Unique to this synthetic scheme are the mild reaction conditions and the absence of reactive intermediates, thereby enabling the installation of strong electron-withdrawing substituents, such as FBZ, BTD, and MI, which were inaccessible through previous synthetic protocols of heteropines and P-containing cyclic materials.^33–47,52–63^

Unlike carbon-based phosphepines, these diazaphosphepines are highly air-stable because the electronegative N atoms draw electron densities away from the P(iii) center.^64,65^ We also can further functionalize their P-center. In the presence of H_2_O_2_ (30% in H_2_O), BZ-, FBZ-, BTD-, CP-, MI- and AN*-*P are converted to their oxide derivatives in yields of 80–90%. The oxidation of these diazaphosphepines requires four days for complete conversion, which suggests their resistance against oxidation under ambient conditions. That our compounds are stable against moisture is perhaps not surprising given previous studies that have shown aminophosphines having pyrrole moieties to also be stable in water and alcohols. This phenomenon was attributed to the participation of N lone-pair electrons in the ring resonance.^66,67^^31^P NMR spectra of the diazaphosphepines confirm the presence of two distinct classes of π-substituents in these species stemming from the different electronic character of the substituents; the ^31^P NMR spectra of BZ-, FBZ-, BTD*-*Ps exhibit chemical shifts in the range of 38.5–38.7 ppm, which are up-field shifted compared to those of CP-, MI- and AN*-*Ps (40.3–44.4 ppm). All diazaphosphepines have been fully characterized, the results of which are summarized in ESI.†

### Crystal structures of diazaphosphepines

**Fig. 1:**
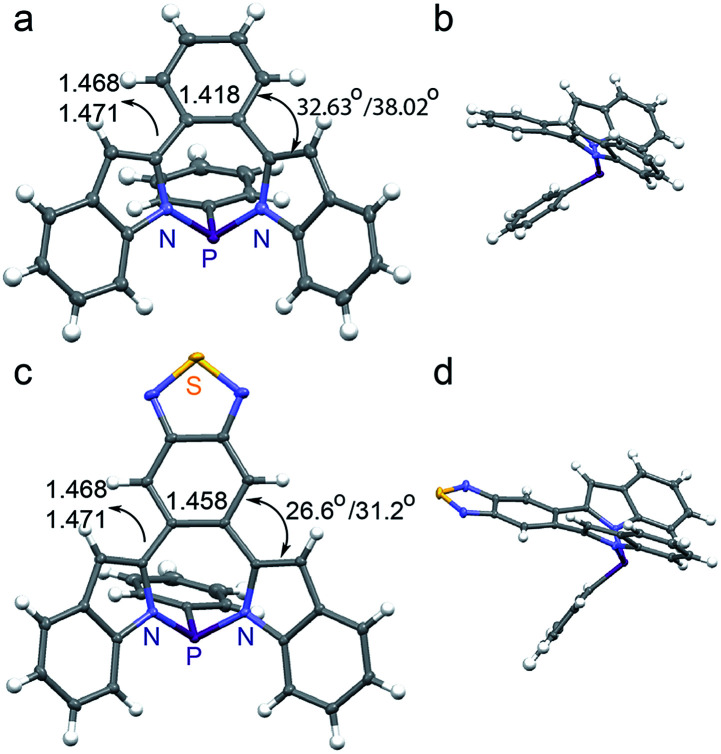
Crystal structures of (a and b) BZ-P and (c and d) BTD-P. Torsion angles and C–C/C=C lengths [Å] are included.

We were able to obtain high quality single crystals of BZ-P, BTD-P, MI-PO, AN-P and AN-PO. Not unlike prior N/S/Si/P-containing heteropines, the crystal structures of BZ-P (Fig. 1a and b) and BTD-P (Fig. 1c and d) reveal twisted backbones, with torsion angles of 32.6° and 38.0° between BZ and its two *ortho*-indole moieties in BZ-P, and 26.6° and 31.2° between BTD and its *ortho*-indole moieties in BTD-P. The crystal structures of BZ-P and BTD-P also reveal elongation of the [*d*]-C=C double bond of BZ and BTD that now bridges the two *ortho*-indole moieties. This [*d*]-C=C double bond is, for example, 1.418(2) Å in BZ-P compared to 1.392 Å in free-standing benzene,^68^ and 1.458(1) Å in BTD-P compared to 1.42 Å in free-standing benzothiadiazole.^69^ Concurrently, the [*c*,*e*]-C–C single bonds that connect BT and BZ to the indole moieties in BZ-P (1.473(2) and 1.477(2) Å) and BTD-P (1.468(1) and 1.471(1) Å) are shorter than typical C–C single bonds (1.533(3) Å).^70^ Collectively, these observations imply some π-electron delocalization between the BZ and BTD substituents and the indole moieties in BZ-P and BTD-P despite the lack of coplanarity.

**Fig. 2:**
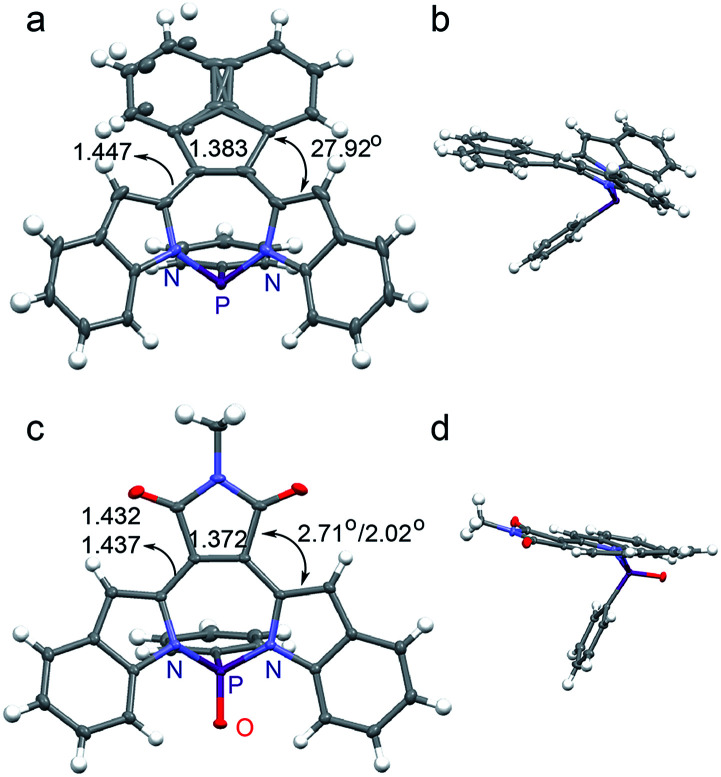
Crystal structures of (a and b) AN-P and (c and d) MI-PO (torsion angles and C–C/C=C length [Å] are shown).

The crystal structures we have examined thus far are aligned with our expectation based on the electronics of the substituents. We are thus surprised that the crystal structure of MI-PO (Fig. 2c and d) is substantially more planar than those of the other diazaphosphepines. The torsion angles between the MI and the indole moieties in MI-PO are only 2.71° and 2.02° for one isomer, and 2.96° and 12.98° for its second isomer (Fig. S3†), which are much smaller than the torsion angles of BZ-P, BTD-P and AN-P, as well as those of non-aromatic heteropines.^52–56^ Such small torsion angles are reminiscent of those in aromatic borepins (ranging from 1–20°) that adopt planar conformations.^41–47^ MI exhibits weaker electronic confinement; its incorporation on the heteropine core thus promotes a more planar conformation compared to the other substituents. Additionally, intramolecular H-bonding can take place between the carbonyl group of MI and the protons on the indole moieties; this interaction should also help planarize MI-PO. MI-PO is the first example of a non-aromatic heteropine adopting a planar backbone. We thus believe substitution at the [*d*]-C=C double bond position to be a promising route for modifying the chemical and electronic structures of heteropines.

The crystal structure of AN-P also reveals a twisted backbone (Fig. 2a and b), presumably due to the sterics of the two adjacent protons on AN and the indole moieties. Compared with BZ-P and BTD-P, AN-P shows a longer [*d*]-C=C double bond and a shorter [*c*,*e*]-C–C single bond, suggesting stronger electronic communication in AN-P. These observations also imply the electronic structure of the [*d*]-substituents to dictate π-electron delocalization in diazaphosphepines, with sterics playing a secondary role. The crystal structure of AN-PO (Fig. S2†) shows comparable [*d*]-C=C double bond length and [*c*,*e*]-C–C single bond length with that of AN-P, suggesting oxidation of the P-center does not affect the electronic communication between AN and the indole moieties in AN-PO.

### Electronic structure of diazaphosphepines

**Fig. 3:**
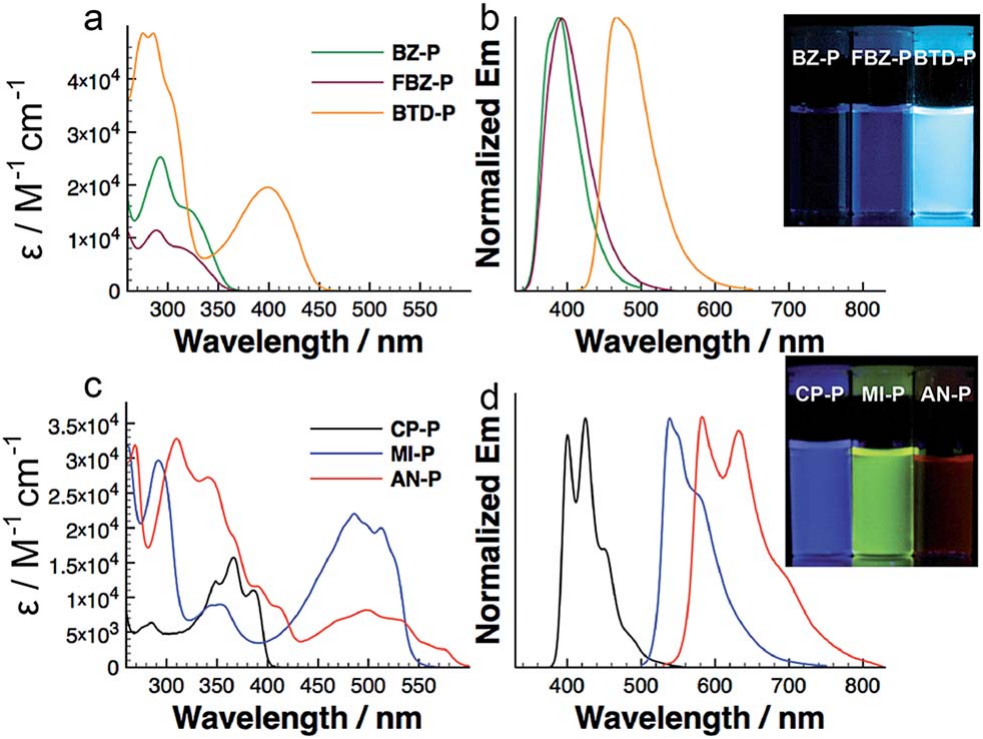
UV-vis absorption and emission spectra of diazaphosphepines in hexane at concentration at 10^−5^ M (photographs reveal their fluorescence under UV light).

We carried out photophysical studies to probe the electronic structures of these diazaphosphepines. We start by comparing BZ-P and CP-P; these compounds comprise the simplest of the aromatic and non-aromatic substitutions, respectively. Fig. 3a and b show that the absorption and emission spectra of CP-P are red-shifted compared to those of BZ-P, despite the fact that BZ has the larger conjugated π-system of the two. Both the absorption and emission spectra of CP-P show strong vibronic structures; these vibronic structures are not observed in the absorption and emission spectra of BZ-P. Additionally, the spectra of CP-P (Δ*λ*_max_ = 974 cm^−1^) show a smaller Stokes shift compared to those of BZ-P (Δ*λ*_max_ = 5641 cm^−1^). The fluorescence quantum yield of CP-P (14.5%) is about three times higher than that of BZ-P (5.4%). Collectively, the photophysics data suggest CP-P to exhibit a more conjugated and rigid structure compared to BZ-P. This assertion is further consistent with our theoretical calculations that reveal CP-P to possess a smaller HOMO–LUMO gap (3.52 eV) compared to BZ-P (4.12 eV; *vide infra*).

Given the local aromaticity of BZ, the π-electrons of the [*d*]-C=C double bond experiences strong electronic confinement. This electronic confinement prevents efficient intramolecular electronic communication between the two indole moieties. In contrast, the π-electrons of the same C=C double bond in CP are less electronically confined and should instead allow more efficient electronic communication between its two indole moieties. This hypothesis is further supported by the comparison between their precursors BZ-In and CP-In that is detailed in Fig. S7.† The absorption spectrum of BZ-In is blue-shifted compared to that of CP-In. In addition to electronic effect of the [*d*]-C=C double bond, the twisted structure observed in crystal of BZ-P could also limit efficient electron delocalization.

We are able to further fine-tune the photophysical properties of diazaphosphepines through the introduction of other electron-donating and electron-withdrawing substituents (Fig. 3). In general, introducing electron-poor FBZ and BTD results in intramolecular charge transfer (ICT) character and higher fluorescence quantum yields in FBZ-P and BTD-P compared to BZ-P. BTD-P exhibits a 84 nm red shift in its largest absorption *λ*_max_ compared to that of BZ-P. This effect is yet more pronounced when non-aromatic MI and AN substituents are introduced to the heteropine core. We observe a 120 nm red shift in the largest absorption *λ*_max_ of MI-P compared to that of CP-P, presumably due to the ICT character that is present in MI-P but not in CP-P (Fig. S8†). In fact, the absorption spectrum of MI-P is also red-shifted compared to that of BTD-P despite the fact that the conjugated π-system of MI is smaller than that of BTD. We ascribe this peculiarity to the strong π-conjugation of the MI-P backbone induced by its stronger C=C double bond character. It follows that the introduction of AN allows a further red shift in the absorption of AN-P compared to the MI-P. Since both absorption and emission spectra of AN-P do not strongly depend on solvent polarity (Fig. S9†), it is reasonable to conclude that its red-shifted absorption is not due to ICT, but mainly due to a further extension of π-conjugation in the twisted AN-P. The ease with which we are able to tune the photophysical properties of diazaphosphepines is unique as previous functionalization protocols of heteropines to modify the optical bandgap has only been met with limited success (*λ*_max_ = 300–450 nm).^41–51^

In addition to tuning the absorption of these diazaphosphepines over a broad range (*λ*_max_ = 315–574 nm), the introduction of these substituents also tunes the emission spectra of these compounds broadly (*λ*_max_ = 389–589 nm). To our best knowledge, these diazaphosphepines are the first examples of luminescent phosphepines. Unlike diazaphosphepines, the backbones of previous phosphepines are less rigid; these could be channels for non-radiative decay.^33,52,53^ Comparing more generally to heteropines that luminesce, AN-P is particularly intriguing since it emits low-energy red luminescence previously not observed. The fluorescence quantum yields of CP-P, MI-P, and AN-P range from 13–67%; such yields are substantially higher than that exhibited by naphthoborepin (1%) having a comparable backbone.^41^ This observation is also consistent with the higher oscillator strengths obtained with CP-P, MI-P, and AN-P compared to naphthoborepin (*f* = 0.14 for CP-P, 0.27 for MI-P, 0.58 for AN-P, *cf. f* = 0.025 for naphthoborepin^41^), and can stem from differences in the electronic structure between non-aromatic diazaphosphepines and the aromatic borepin. The donor–acceptor architecture in BTD-P and MI-P results in larger Stokes shift with increasing solvent polarity (Fig. S8a and b†). By plotting the Stokes shift as a function of solvent polarity (Fig. S8c†), we find BTD-P to show a stronger solvatochromic effect than MI-P.

In fact, the effects of [*d*]-substitution on the photophysical properties of diazaphosphines appear to fall in two broad classes, with BZ-P, FBZ-P and BTD-P defining one class and CP-P, AN-P and MI-P defining the other. The photophysical characteristics of BZ-P, FBZ-P and BTD-P are consistent with those of non-aromatic flexible heteropines having featureless absorption and emission spectra,^52,54–56^ while those of CP-P, AN-P and MI-P share photophysical traits of aromatic heteropines having well-resolved absorption, emission spectra and small Stokes shift.^41–47^ The observation that AN-P shares the same photophysics as CP-P and MI-P further suggests that the electronics of substituents is the primary factor governing electron delocalization of diazaphosphepines, with sterics being a secondary factor. Else, we should not observe evidence for conjugation and rigidity in AN-P given that its backbone is twisted.

The diazaphosphepine oxide derivatives show systematically similar photophysical characteristics to their unoxidized counterparts. For completeness, the photophysical characterization of the oxide analogues is detailed in Fig. S6 and Table S1.† This observation suggests that P-center of the diazaphosphepines is not directly involved in the electronic structure of this seven-membered ring system, which is characteristically different from P-containing five- and six-membered ring systems.^11–15^

### Theoretical studies of diazaphosphepines

**Fig. 4:**
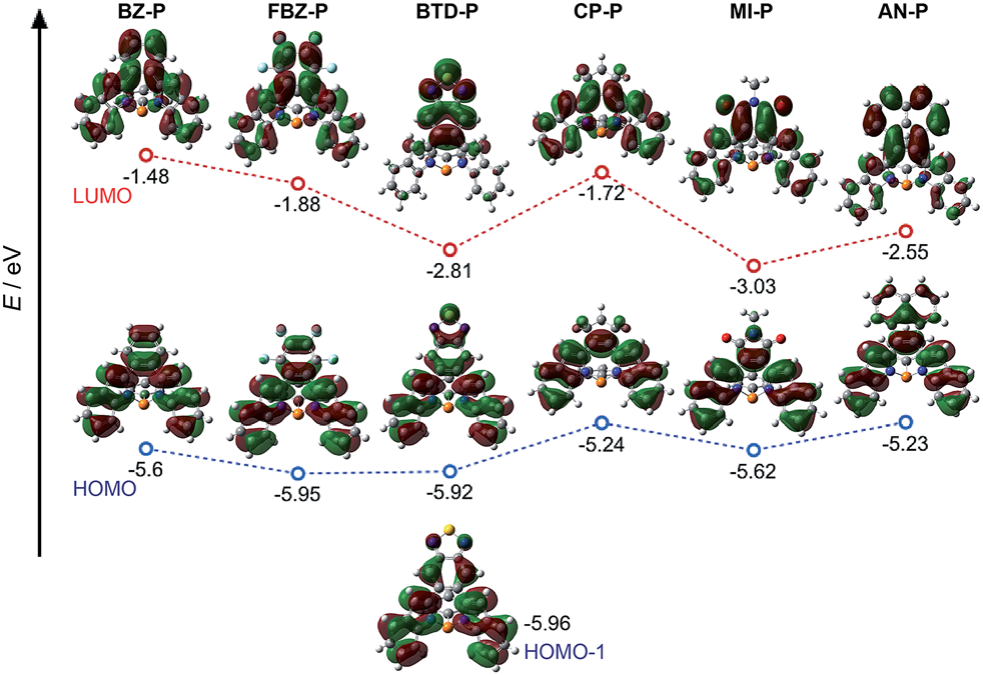
Frontier molecular orbitals of diazaphosphepines based on DFT at B3LYP/6-31+g(d) level.

To further understand their electronic structures, DFT calculations were carried out on these diazaphosphines (see ESI† for details). Their frontier molecular orbitals are provided in Fig. 4; the calculated HOMO and LUMO energy levels are summarized in Table 1. In line with the electronic confinement hypothesis put forth, the HOMOs of BZ-P, FBZ-P and BDT-P show stronger π-electron delocalization of [*d*]-C=C double bond within the substituents compared to HOMOs of CP-P, MI-P and AN-P. Compared to the other compounds in the series, BTD-P has the longest [*d*]-C=C bond, it thus exhibits the weakest C=C double bond character, as evidenced by its degenerate HOMO−1 (Fig. 4).

**Table 1:** Photophysical and redox characteristics of diazaphosphepines

Compound	*λ*_abs_ a [nm]	*λ*_em_ a [nm]	*ϕ* b	*E*_ox_ c	*E*_red_ c	LUMO^d^ [eV]	HOMO^e^ [eV]	LUMO^e^ [eV]
BZ-P	319	389	5.4%	0.86^f^	n.d.^g^	n.d.^g^	−5.60	−1.48
FBZ-P	315	392	14.5%	1.12^f^	n.d.^g^	n.d.^g^	−5.95	−1.88
BTD-P	399	466	19.3%	n.d.^g^	−1.72	−3.08	−5.92	−2.81
CP-P	385	400	13.9%	0.65^h^	n.d.^g^	n.d.^g^	−5.24	−1.72
MI-P	512	538	66.8%	0.87^f^	−1.35^f^, −1.94^f^	−3.45	−5.62	−3.03
AN-P	574	589	15.2%	0.51^f^, 0.81^f^	−1.79^f^	−3.01	−5.23	−2.55

a Absorption and emission maximum measured in hexane.

b Fluorescence QY determined using a calibrated integrating sphere system in CH_2_Cl_2_.

c *vs.* Fc^0/+^, 0.1 M Bu_4_N[PF_6_] in CH_2_Cl_2_.

d LUMO determined by −(4.8 + *E*_red,1/2_).

e DFT calculated at B3LYP/6-31+g(d) level.

f Quasi-reversible or reversible (*E*_red_(*E*_ox_) = 1/2(*E*_pc_ + *E*_pa_)).

g Not detected in the solvent range.

h Irreversible (*E*_red_(*E*_ox_) = *E*_pc_(*E*_pa_)).

We further carried out nucleus-independent chemical shift (NICS) calculations to verify the aromatic character of the central seven-membered P-ring in our diazaphosphepines at the GIAO/B3LYP/6-31+g(d) level of theory. The NICS(1) values are all near-0, implying the non-aromatic character of these P-containing seven-membered rings (Table S2†). This result begs the question – then – of why the photophysical character of CP-P, AN-P and MI-P resembles those of aromatic, as opposed to non-aromatic, heteropines. We believe this discrepancy can be addressed by comparing the calculated HOMOs of CP-P, AN-P and MI-P with those of dibenz-/dithieno[*b*,*f*]borepins and dibenz[*b*,*f*]silepins reported in the literature.^41,47,56^Fig. 4 confirms that the P-centers do not contribute to either the HOMOs or the LUMOs of diazaphosphepines. While B-center contributes electron delocalization in the HOMOs of dibenz-/dithieno[*b*,*f*]borepins.^41,47^ Furthermore, unlike the HOMOs of dibenz-/dithieno[*b*,*f*]borepins and dibenz[*b*,*f*]silepins, the HOMOs of CP-P, AN-P and MI-P show weak double-bond character at the [*b*,*f*]-N–C bonds of the central heteropine ring, which can in turn induce weak π-electron delocalization within the central seven-membered ring, but strong π-electron delocalization between the [*d*]-substituents and the [*b*,*f*]-substitutes in CP-P, AN-P and MI-P. Collectively, this effect should enhance electron delocalization.

**Fig. 5:**
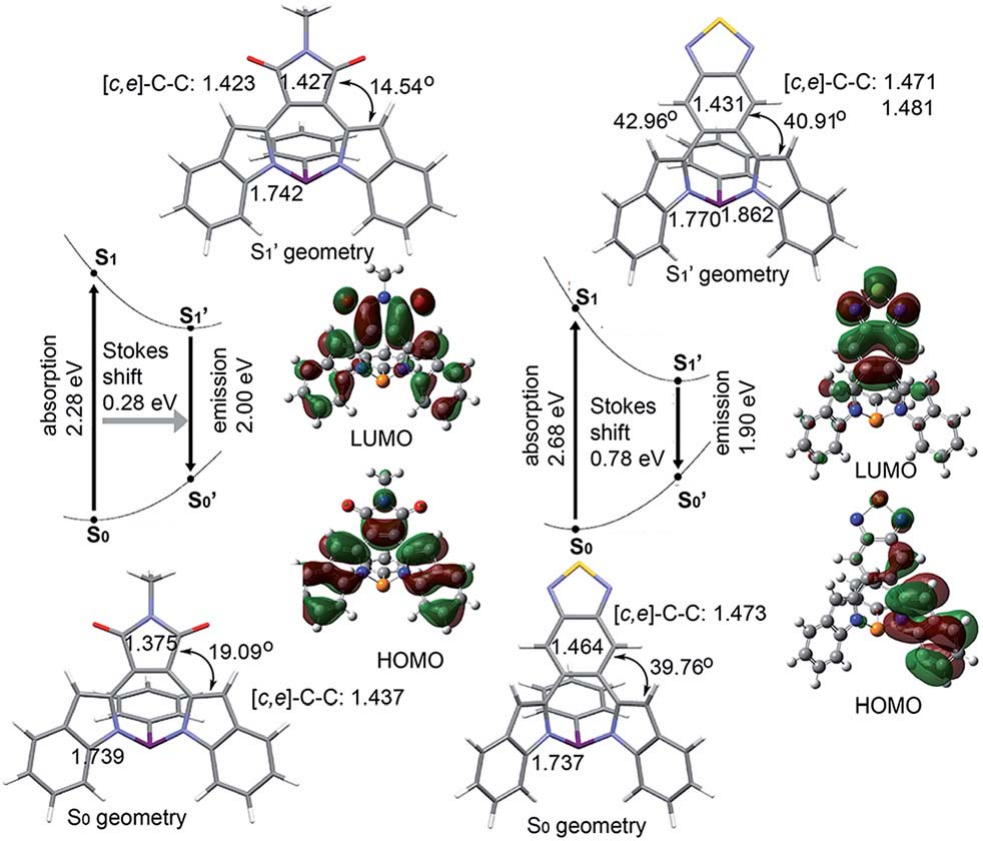
Optimized structures for the S_0_(S′_0_) and S_1_(S′_1_) electronic states, an energy diagram and the S′_1_ state frontier molecular orbitals of MI-P and BTD-P calculated at the B3LYP/6-31+g(d) level (torsion angles and C=C length [Å] are shown).

That BTD-P shows a stronger solvatochromic effect than MI-P seems counter-intuitive since MI is a stronger electron-accepting substituent than BTD.^71^ To further understand the solvatochromism effect in the emission spectra of BTD-P and MI-P, we carried out theoretical calculations on their excited states. The excited-state structures were optimized by TD-DFT. Their HOMOs and LUMOs were calculated at the B3LYP/6-31+g(d) level. In Fig. 5, MI-P shows enhanced planarization in the S′_1_ state along with an elongation of the [*d*]-C=C double bond, a shortening of the [*c*,*e*]-C–C single bonds and a decrease in the torsion angle compared to its S_0_ state. This comparison suggests enhanced π-conjugation in the excited state. The S′_1_ structure of BTD-P, on the other hand, is more twisted, with a shortening of its [*d*]-C=C double bond and an increase in its torsion angle compared to its S_0_ state. This opposite trend suggests instead decreased π-conjugation in the excited state of BTD-P. Looking more closely at BTD-P, photoexcitation leads to a conformational change from its symmetric structure in its S_0_ state to an asymmetric structure in its S′_1_ state. In its S′_1_ state, the HOMO of BTD-P is mainly localized on one indole moiety, while the LUMO of BTD-P is mainly localized on the BTD substituent. Accordingly, the excited-state molecular dipole moment (2.49 D) is larger than that of its S_0_ state (1.63 D). Both observations point to enhanced intramolecular charge separation (Fig. 5) compared to its S_0_ state as shown in BTD-P's HOMO and LUMO (Fig. 4). These observations are also consistent with the proposed mechanism of twisted ICT in typical donor–acceptor systems.^72^ In fact, the excited-state molecular dipole moment of BTD-P is also larger than that of MI-P (2.40 D). This difference is consistent with the stronger solvatochromic effect observed in the emission spectra of BTD-P compared to that of MI-P. Further, the excited- and ground-state oscillator strengths of BTD-P are smaller compared to those of MI-P, which is also in line with the lower fluorescence quantum yield observed in BTD-P compared to MI-P. Finally, the calculated Stokes shift of BTD-P (0.78 eV) is larger than that of MI-P (0.28 eV), which is also consistent with experimental data.

While the above comparison highlights the ground- and excited-state differences between diazaphosphepines having aromatic (BTD-P) and non-aromatic (MI-P) substituents, the conformation of BTD-P appears unique, even amongst the diazaphosphepines having aromatic substitutions. Whereas the conformations of the excited states of BZ-P and FBZ-P are more planar compared to their ground states (Fig. S11†), the conformation of the excited state of BTD-P is more twisted compared to its ground state. Given this comparison among derivatives having aromatic substituents and similar steric hindrance, the more twisted conformation in the excited state of BTD-P must stem from its weak [*d*]-C=C double bond character. In addition to modulating the ground-state electronic structure of diazaphosphepines, our calculations thus show that [*d*]-C=C engineering tunes the excited state character of these compounds.

### Redox properties of diazaphosphepines

**Fig. 6:**
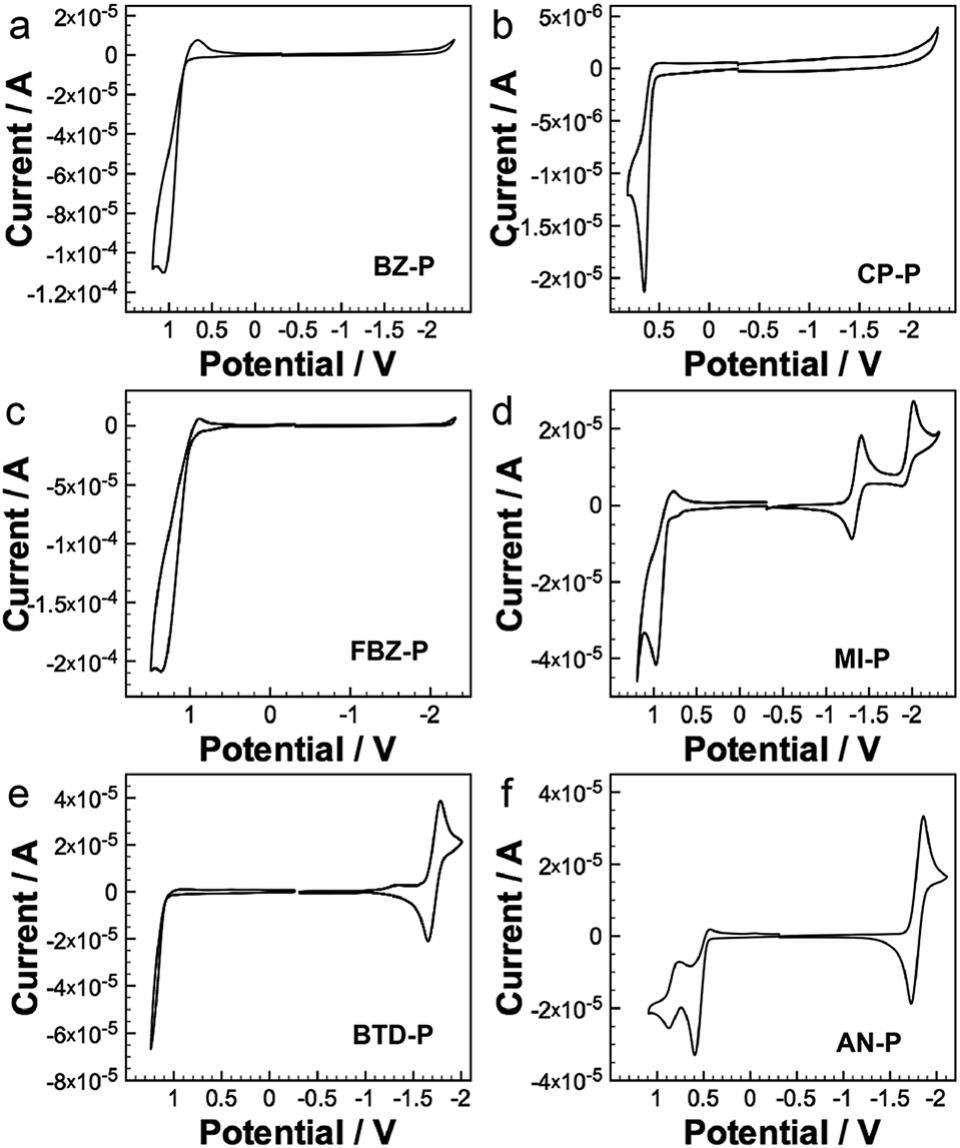
Cyclic voltammograms of our diazaphosphepines.

The introduction of substituents at the [*d*]-position also offers diazaphosphepines rich redox characteristics. The cyclic voltammograms of diazaphosphepines are shown in Fig. 6; the data are summarized in Table 1. Compared to BZ-P (*E*_ox_ = 0.86 V), CP-P shows a smaller oxidation potential at 0.65 V that is consistent with its higher theoretical HOMO energy level and stronger π-conjugation observed in our photophysical studies. Enhancing the electron-accepting characteristics by replacing BZ with FBZ and BTD reduces FBZ-P’s and BTD-P’s susceptibility to oxidation, with BTD-P's oxidation potential outside the scan window. Compared to BZ-P, FBZ-P, and BTD-P, the oxidation potentials of CP-P, MI-P, and AN-P show a weaker dependence on the electronic character of the substituents. In fact, the oxidation potential of MI-P is much lower than those of FBZ-P and BTD-P, despite electron-accepting character of MI is stronger than both of FBZ and BTD. Such easy oxidation of CP, MI, and AN derivatives implies that strong electron delocalization along the backbone of CP-P, MI-P, and AN-P dominates their redox character. This observation is consistent with the trend of theoretical HOMO energy levels determined for these compounds.

Of the diazaphosphepines, only BTD-P, MI-P, and AN-P show reversible reduction within the scan range. These reduction potentials are universally less negative than those of any other heteropines (−1.8 V to −2.6 V),^41–47,52–56^ including borepins containing an electron-deficient boron center, that have been reported to-date. We also estimated the LUMO energy level of these compounds based on their reduction potentials. Introducing a stronger electron-accepting substituent, such as MI, significantly lowers the LUMO energy level of MI-P compared to BTD-P and AN-P. While oxidation of the phosphorus center does not affect their photophysical properties, it generally lowers the LUMO energy levels in these compounds, (Fig. S12†) with the LUMOs of BTD-PO, MI-PO and AN-PO estimated at −3.18 V, −3.57 V and −3.09 V respectively, due to the electron-withdrawing character of the PO center.

### P–N chemistry enabling highly electron-deficient heteropines

**Scheme 2:**
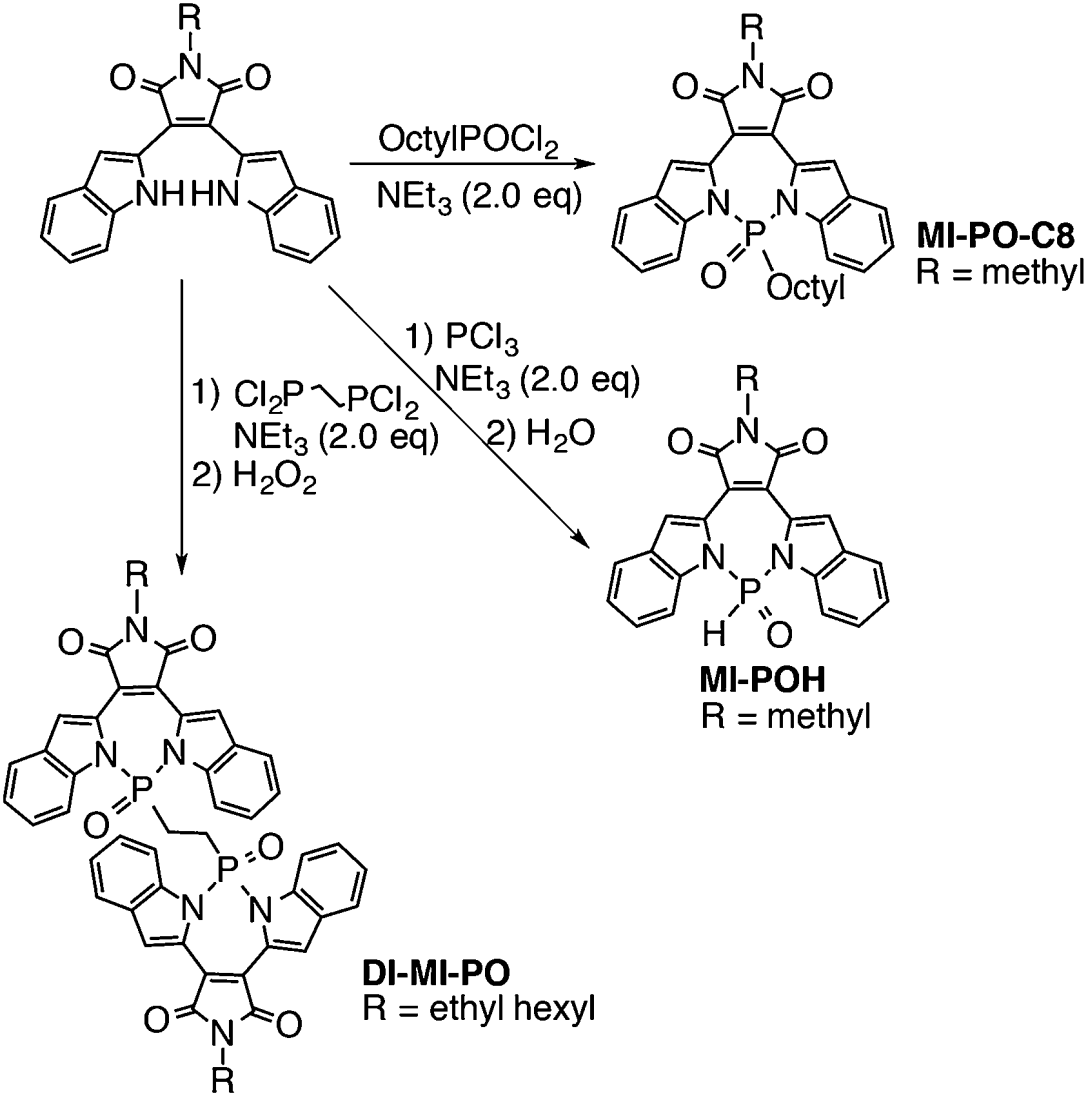
Synthesis of MI-derivatives *via* P–N chemistry; the photograph reveals the fluorescence of Di-MI-PO in different solvents.

The highly electron-deficient character of MI-PO determined in the CV experiments promises its potential application as non-fullerene acceptors. To further optimize its intermolecular packing in the solid state, we replaced the bulky phenyl group on the P-center of MI-PO with a proton. Using PCl_3_ as the starting reagent, we have obtained MI-POH with 82% yield. However, MI-POH exhibits low solubility in organic solvents. To increase solubility but still retain its less bulky characteristic compared to MI-PO, we have additionally synthesized MI-PO-C8 and Di-MI-PO (Scheme 2) using commercially available phosphorus reagents under similar reaction conditions.

**Fig. 7:**
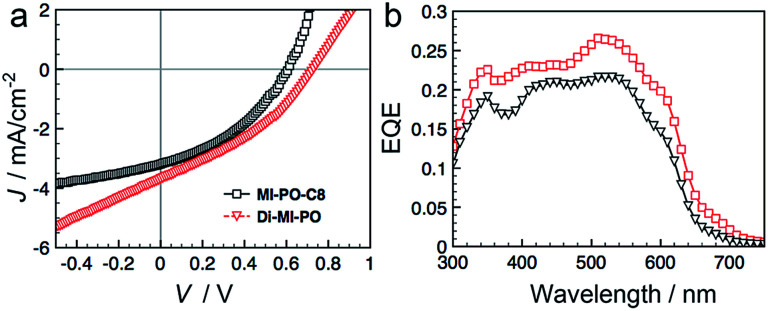
(a) *J*–*V* characteristics and (b) EQE of bulk-heterojunction solar cells containing MI-PO-C8 or Di-MI-PO as the electron acceptor and P3HT as the electron donor.

We fabricated organic solar cells with Di-MI-PO or MI-PO-C8 as electron acceptors and poly(3-hexyl thiophene) as electron donor. The performance metrics of these devices are provided in Table S3.† The *J*–*V* characteristics of representative devices are shown in Fig. 7a. Of four devices containing MI-PO-C8 tested, we obtain an average power-conversion (PCEs) of 0.71 ± 0.02%, with a short-circuit current density (*J*_sc_) of 3.07 ± 0.1 mA cm^−2^, an open-circuit voltage (*V*_oc_) of 0.61 ± 0.01 V, and a fill factor (FF) of 38 ± 0.5%. Devices containing Di-MI-PO exhibit higher PCEs of 1.04 ± 0.02%, with *J*_sc_ of 3.77 ± 0.7 mA cm^−2^, *V*_oc_ of 0.71 ± 0.02 V, and FF of 39 ± 3%. Compared to those with MI-PO-C8, devices containing Di-MI-PO show higher *J*_sc_, which is consistent with its higher EQE in Fig. 7b. Additionally, devices containing Di-MI-PO also show higher *V*_oc_; this observation is consistent with its higher LUMO (−4.2 eV *versus* −4.0 eV).^73^ Interestingly, the *V*_oc_ of devices containing Di-MI-PO is 100 mV higher than devices having phenyl-C_61_-butyric acid methyl ester, PCBM, as the electron acceptor despite the observation that Di-MI-PO exhibits a lower LUMO energy level compared to PCBM (−3.8 eV).^74^ We thus speculate that the presence of symmetry-breaking charge transfer in Di-MI-PO also contributes to the *V*_oc_ enhancement in its devices (see ESI† this assertion).

## Conclusions

By leveraging the versatility of P–N chemistry, we demonstrated that we can engineer the [*d*]-C=C double bond in functionalized heteropines, and accordingly modify the properties of these materials. Both the electronics and sterics of the substituents impact the strength of the [*d*]-C=C double bond, providing access to electronic properties previously inaccessible in both the ground- and excited-states of heteropines. Particularly, our strategy allows us to access heteropines having much broader absorption and emission compared to the previous protocols. The MI-substituted diazaphosphepine exhibits planar π-conjugated structure that has not been accessed in non-aromatic heteropines. Having the weakest [*d*]-C=C double bond character of the current series, the BTD-substituted diazaphosphepine exhibits twisted ICT in its excited state. This excited-state conformation contrasts the planar conformations of previous heteropines in their excited states. With the same chemistry, we have introduced electron-accepting substituents at the [*d*]-C=C double bond, which has resulted in compounds having more electron deficient character than any previously reported heteropines. Despite their low photocurrents, the operation of devices comprising these materials demonstrates—for the first time—the viability of heteropines as electron acceptors for organic photovoltaics.

## Supplementary Material

SC-007-C6SC00519E-s001

SC-007-C6SC00519E-s002
