# Supplementary material for: [d]-Carbon–carbon double bond engineering in diazaphosphepines: a pathway to modulate the chemical and electronic structures of heteropines
Source: Chem Sci. 2016 Mar 15;7(7):4211–9. doi: 10.1039/c6sc00519e (PMC6014086; doi:10.1039/c6sc00519e)
Supplement: SC-007-C6SC00519E-s001 [file SC-007-C6SC00519E-s001.pdf]

Electronic Supplementary Material (ESI) for Chemical Science.
This journal is © The Royal Society of Chemistry 2016

[*d*]-Carbon-Carbon Double Bond Engineering in Diazaphosphepines: A Pathway to Modulate the Chemical and Electronic Structures of Heteropines

Yi Ren,[a] Melda Sezen,[a] Fang Guo,[b] Frieder Jäkle,[b] Yueh-Lin Loo[a]

[a]Department of Chemical and Biological Engineering, Princeton University, NJ 08544 (USA)

[b] Department of Chemistry, Rutgers University, Newark, NJ 07102 (USA)

Index **Page**

Index	Page
Materials and Experiments	S2
Synthetic Procedures	S3-S11
Crystal Structures	S12-S13
Photophysics Data	S14-S16
Optimized structure at S_1’ state	S17
Cyclic Voltammograms	S18
Photophysics Data of R-Substituted Diazaphosphepins	S19
Theoretical Calculations	S20
Devices Characteristics and GIXD Images of Blends	S21-S23
NMR Spectra	S23-S50
References	S51

Materials and Experiments

General. All manipulations were carried out under a dry nitrogen atmosphere employing standard Schlenk techniques. Reagents were purchased from Sigma-Aldrich and Alfa Aesar, and were, unless otherwise noted, used as-received. NMR solvents were purchased from Cambridge Isotope Laboratories. ^{1}H-NMR, ^{13}C{1H}-NMR, and ^{31}P {1H} NMR were recorded on Bruker Avance (III) 300 and 500 MHz spectrometers. High-resolution mass spectra were obtained by Dr. Brandon Fowler of the Mass Spectroscopy Facility at Columbia University and John Eng of the Mass Spectroscopy Facility at Princeton University. We were not able to obtain high-resolution mass spectra on the other compounds as they degraded under the conditions at which they were ionized. The crystal structures of **BZ-P** (CCDC1427667), **BTD-P** (CCDC1427666), **MI-PO** (CCDC1427668), **AN-P** (CCDC1427664) and **AN-PO** (CCDC1427665) were obtained by Dr. Philip Jeffrey of the X-ray Crystallography Facility at Princeton University. GIXD experiments were conducted at the G1 station of the Cornell High Energy Synchrotron Source. UV-vis experiments were carried out on a UV-vis-NIR Cary 5000 spectrophotometer. The fluorescence measurements were performed using a Hitachi F-7000 fluorescence spectrophotometer. Absolute quantum yields were obtained with a pre-calibrated Quanta-φ integrating sphere attached to a Fluorolog-3 instrument. Cyclic voltammetry experiments were carried out with a Pt disk as the working electrode, a Pt wire as the counter electrode and a Ag wire as the pseudo-reference electrode. The voltammograms were recorded with ca. 10^{-3} to 10^{-4} M solutions in CH_2Cl_2 containing $Bu_4N[PF_6]$ (0.1 M) as the supporting electrolyte. Theoretical calculations were carried out using the GAUSSIAN 03 suite of programs.[S1]

Device Fabrication. Patterned ITO (15 Ω/sq) on glass substrates was coated with 30-nm thick poly(3,4-ethylenedioxythiophene):poly-(styrenesulfonate), PEDOT:PSS (Clevios P), followed by thermal annealing at 150 °C for 10 min. The PEDOT:PSS was diluted with distilled water at a 1:1 volume ratio prior to use. The organic bulk-heterojunction layer was obtained by spin-coating solutions of P3HT and **MI-PO-C8** (1:2 wt) and P3HT and **Di-MI-PO** (1:4 wt) at 1200 rpm for 60 s atop the PEDOT:PSS layer. The total concentration of donor and acceptor in these solutions was kept constant at 20 mg/mL in $CHCl_3$. Aluminum (80 nm) top electrodes were thermally evaporated through a shadow mask at a pressure of 10^{-6} bar and an evaporation rate of 0.8 Å/s to define an active area of 0.18 cm^2. Devices containing P3HT and **MI-PO-C8** were annealed at 100 ^{o}C for 10 min. Devices containing P3HT and **Di-MI-PO** were annealed at 120 ^{o}C for 10 min. Current density–voltage (J-V) characteristics were acquired using a Keithley 2635 source measurement unit under AM 1.5G 100 mW/cm^2 illumination in a nitrogen-filled glovebox (<0.1 ppm of O_2 and H_2O).

Synthetic Procedures

General synthesis of ***B***- and ***O*-In**

In a 1-necked 150-mL Schlenk flask, dibromo or diiodo moieties (1.0 mole eq.) was mixed with indole-2-boronic acid pinacol ester (2.2 mole eq.), tris(dibenzylideneacetone)dipalladium(0) (0.05 mole eq.), tri-tert-butylphosphonium tetrafluoroborate (0.25 mole eq.), and potassium phosphate (9.0 mole eq.) in a mixture of THF and water (4:1 v/v) or toluene and water (4:1 v/v). The resulting mixture was degassed for 10 min, then refluxed under argon overnight. The reaction mixture was allowed to cool to room temperature, after which it was poured into 10 mL of water. The organic layer was extracted with chloroform. The crude product was purified by flash chromatography using dichloromethane and hexanes as eluent (from 1:9 to 4:6 by volume) to obtain ***B***- and ***O*-In**.

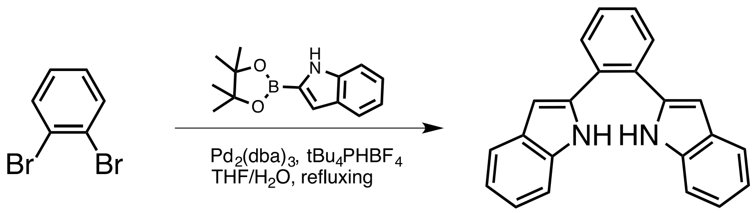

BZ-In was obtained as a white solid (Yield: 64%). ^{1}H NMR ($CDCl_3$, 500 MHz, δ): 7.94 (s, 2H), 7.66 – 7.64 (m, 2H), 7.61 – 7.59 (m, 2H), 7.43 – 7.41 (m, 2H), 7.11 – 7.06 (m, 6H), 6.68 (s, 2H) ppm. ^{13}C NMR ($CDCl_3$, 137.3, 136.8, 131.1, 131.0, 128.7, 128.6, 122.7, 120.8, 120.5, 111.4, 102.9 ppm. Due to the instability of this compound in the solution, HRMS could not be obtained by using ESI experimental conditions.

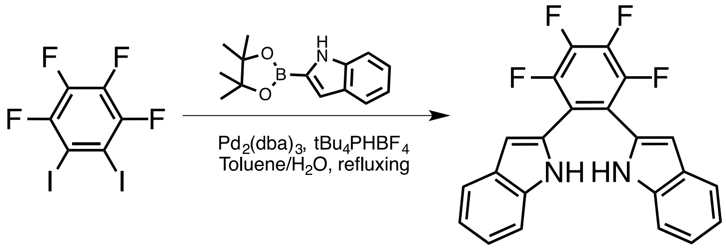

FBZ-In was obtained as a white solid (Yield: 56%). ^{1}H NMR ($CDCl_3$, 500 MHz, δ): 7.91 (s, 2H), 7.59 (d, J = 7.1 Hz, 2H), 7.12 – 7.06 (m, 6H), 6.71 (s, 2H) ppm. ^{13}C NMR ($CDCl_3$, 125 MHz, δ): 136.7, 128.0, 126.2, 123.7, 121.4, 120.8, 111.5, 107.1 ppm. HRMS: m/z = 378.0775 ($[M-2H]^+$, Calcd. 378.0780).

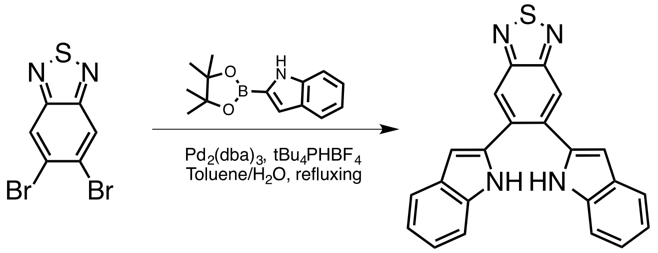

BTD-In was obtained as an orange solid (Yield: 67%). ^{1}H NMR ($CDCl_3$, 500 MHz, δ): 8.25 (s, 2H), 7.97 (s, 2H), 7.63 (d, J = 7.4 Hz, 2H), 7.14 – 7.09 (m, 6H), 6.77 (d, J = 2.1 Hz, 2H) ppm. ^{13}C NMR ($CDCl_3$, 125 MHz, δ): 154.6, 137.2, 135.7, 133.7, 128.4, 123.4, 122.7, 121.2, 120.8, 111.5, 104.5 ppm. HRMS: m/z = 367.1013 ($[M+H]^+$, Calcd. 367.1017).

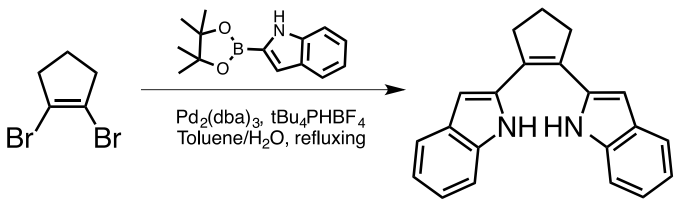

CP-In was obtained as a light yellow solid (Yield: 56%). ^{1}H NMR ($CDCl_3$, 500 MHz, δ): 8.42 (s, 2H), 7.58 (d, J = 7.8 Hz, 2H), 7.24 – 7.23 (m, 2H), 7.24 – 7.23 (m, 2H), 7.17 – 7.14 (m, 2H), 7.11 – 7.07 (m, 2H), 6.61 (s, 1H), 2.98 (t, J = 7.5 Hz, 4H), 2.09 (q, J = 7.6 Hz, 2H) ppm. ^{13}C NMR ($CDCl_3$, 125 MHz, δ): 136.5, 135.07, 128.9, 128.6, 123.0, 120.8, 123.0, 120.8, 120.5, 111.1, 103.5, 38.2, 22.3 ppm. HRMS: m/z = 299.1551 ($[M+H]^+$, Calcd. 299.1548).

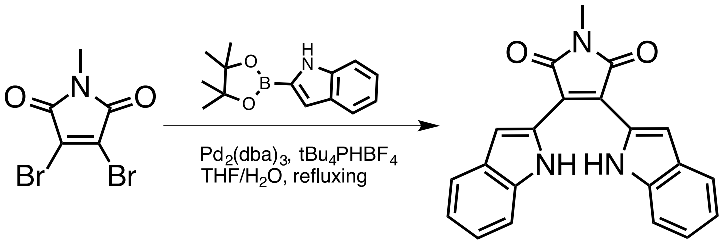

MI-In was obtained as a purple solid (Yield: 59%). ^{1}H NMR ($CDCl_3$, 500 MHz, δ): 10.10 (s, 2H), 7.65 (d, J = 0.6 Hz), 7.65 (d, J = 8.0 Hz, 2H), 7.42 (d, J = 8.2 Hz, 2H), 7.28 (t, J = 7.6 Hz, 2H), 7.13 (t, J = 7.5 Hz, 2H), 3.15 (s, 3H) ppm. ^{13}C NMR ($CDCl_3$, 125 MHz, δ): 172.3, 137.8, 128.0, 125.6, 122.1, 121.9, 121.3, 119.1, 108.5, 30.0, 24.6 ppm. HRMS: m/z = 342.1220 ($[M+H]^+$, Calcd. 342.1242).

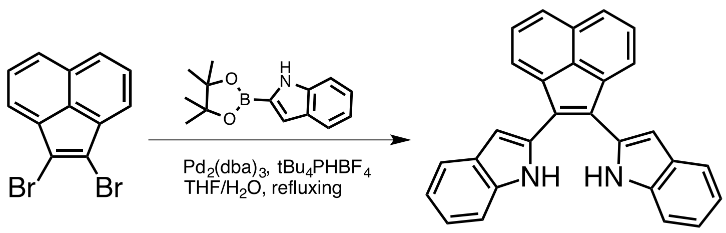

AN-In was obtained as a red solid (Yield: 62%). ^{1}H NMR ($CDCl_3$, 500 MHz, δ): 8.36 (s, 2H), 7.97 (d, J = 6.9 Hz, 2H), 7.86 (d, J = 8.1 Hz, 2H), 7.70 (d, J = 7.7 Hz, 2H), 7.61 (d, J = 15.1 Hz, 2H), 7.25 (d, J = 8.0 Hz, 2H), 7.20 (d, J = 8.0 Hz, 2H), 7.16 (d, J = 14.8 Hz, 2H), 7.05 (d, J = 1.6 Hz, 2H) ppm. ^{13}C NMR ($CDCl_3$, 125 MHz, δ): 139.3, 136.8, 132.3, 128.9(4), 128.8(9), 128.3(2), 128.2(9), 128.1, 124.6, 123.2, 121.0, 111.3, 105.1 ppm. HRMS: m/z = 383.1546 ($[M+H]^+$, Calcd. 383.1548).

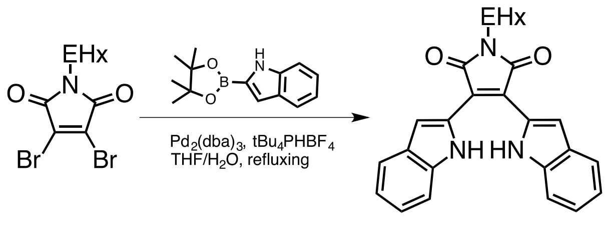

EHx-MI-In was obtained as a purple solid (Yield: 52%). ^{1}H NMR ($CDCl_3$, 300 MHz, δ): 10.08 (s, 2H), 7.78 – 7.71 (m, 2H), 7.65 (d, J = 8.7 Hz, 2H), 7.41 (d, J = 8.3 Hz, 2H), 7.30 – 7.25 (m, 2H), 7.16 – 7.11 (m, 2H), 3.53 (d, J = 7.1 Hz, 2H), 1.82 – 1.74 (m, 1H), 1.36 – 1.29 (m, 8H), 0.94 – 0.86 (m, 6H) ppm. ^{13}C NMR ($CDCl_3$, 125 MHz, δ): 172.6, 137.8, 128.1, 128.0, 125.5, 121.9, 121.8, 121.2, 111.9, 108.4, 42.5, 38.8, 30.8, 28.9, 24.2, 23.3, 14.4, 10.7 ppm. HRMS: m/z = 440.2322 ($[M+H]^+$, Calcd. 440.2338).

General synthesis of mono-substituted **m*B*-** and ***O*-In**

In a 1-necked 150-mL Schlenk flask, dibromo or diiodo moieties (1.0 mole eq.) was mixed with indole-2-boronic acid pinacol ester (2.0 mole eq.), tris(dibenzylideneacetone)dipalladium(0) (0.05 mole eq.), tri-tert-butylphosphonium tetrafluoroborate (0.25 mole eq.), and potassium phosphate (9.0 mole eq.) in a mixture of THF and water (4:1 v/v). The resulting mixture was degassed for 10 min, then refluxed under argon overnight. The reaction mixture was allowed to cool to room temperature, after which it was poured into 10 mL of water. The organic layer was extracted with chloroform. The crude product was purified by flash chromatography using dichloromethane and hexanes as eluent (from 1:9 to 4:6 by volume).

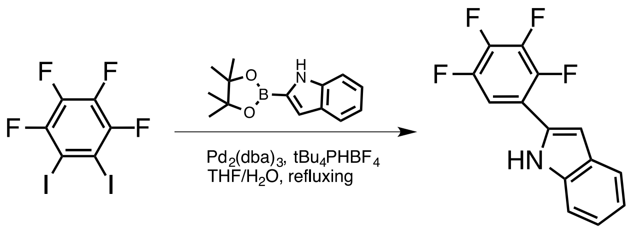

mFBZ-In was obtained as a white solid (Yield: 52%). ^{1}H NMR ($CDCl_3$, 500 MHz, δ): 7.63 (s, 1H), 7.41 (d, J = 8.2 Hz, 1H), 7.35 – 7.30 (m, 1H), 7.25 – 7.22 (m, 1H), 5.13 (t, J = 7.5 Hz, 1H), 6.89 (d, J = 1.9 Hz, 1H) ppm. ^{13}C NMR ($CDCl_3$, 125 MHz, δ): 147.8 (d, J = 240.7 Hz), 144.8 (d, J = 233.1 Hz), 139 (d, J = 26.08 Hz), 137.2, 129.8, 128.11, 123.9, 121.3, 121.1, 111.5, 108.7 (dt, J = 21.0, J = 3.8 Hz), 103.6 ppm. HRMS: m/z = 266.0585 ($[M+H]^+$, Calcd. 266.0593).

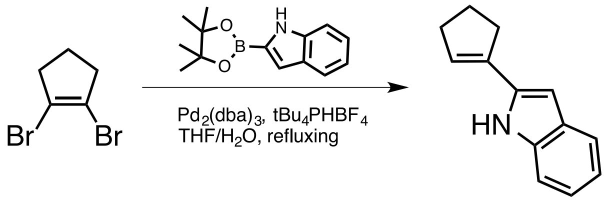

mCP-In was obtained as a white solid (Yield: 43%). ^{1}H NMR ($CDCl_3$, 500 MHz, δ): 8.08 (s, 1H), 7.53 (d, J = 7.8 Hz), 7.29 (d, J = 8.0 Hz, 2H), 7.12 (d, J = 15.8 Hz, 2H), 7.04 (d, J = 15.6 Hz, 2H), 6.40 (s, 1H), 5.99 (s, 1H), 2.75 – 2.72 (m, 2H), 2.56 – 2.53 (m, 2H), 2.04 – 1.98 (m, 2H) ppm. ^{13}C NMR ($CDCl_3$, 125 MHz, δ): 136.8, 136.7, 135.2, 129.2, 124.9, 122.5, 120.8, 120.1, 110.7, 33.7, 33.3, 23.5 ppm. HRMS: m/z = 184.1125 ($[M+H]^+$, Calcd. 184.1126).

General Synthesis of ***B-*** and ***O-*****P**s: In a 1-necked 150-mL Schlenk flask, phenylphosphine dichloride (1.0 mole eq.) was added to an acetonitrile solution of ***B-*** or ***O-*****In** (1.0 mole eq.) in the presence of triehtylamine (2.0 mole eq.) at 0 ^oC. The resulting mixture was refluxed under argon overnight. The reaction mixture was allowed to cool to room temperature. Acetonitrile was removed under vacuum. The crude product was purified by flash chromatography using dichloromethane and hexanes as eluent (from 1:9 to 9:1 by volume) to obtain **B-** and ***O-*** **P**.

BZ-P was obtained as a white solid (Yield: 55%). ^{1}H NMR ($CDCl_3$, 500 MHz, δ): 8.03 (d, J = 8.3 Hz, 2H), 7.61 (d, J = 7.8 Hz, 2H), 7.41 – 7.39 (m, 2H), 7.30 (t, J = 7.7 Hz, 2H), 7.19 (t, J = 15.0 Hz, 2H), 7.12 – 7.10 (m, 2H), 7.02 – 6.94 (m, 3H), 6.87 – 6.84 (m, 2H), 6.82 (s, 2H) ppm. ^{31}P NMR ($CDCl_3$, 121 MHz, δ): 38.5 ppm. ^{13}C NMR ($CDCl_3$, 125 MHz, δ): 143.3 (d, J = 6.7 Hz), 142.8 (d, J = 26.7 Hz), 137.1, 131.9, 130.6, 129.8 (d, J = 4.8

Hz), 129.3, 129.1, 128.5, 128.3 (d, J = 4.8 Hz), 123.2, 121.9, 120.9, 112.0, 109.3, ppm. HRMS: m/z = 415.1360 ($[M+H]^+$, Calcd. 415.1359).

FBZ-P was obtained as a white solid (Yield: 57%). ^1H NMR ($CDCl_3$, 500 MHz, δ): 8.00 (d, J = 8.4 Hz, 2H), 7.62 (d, J = 7.9 Hz, 2H), 7.34 (t, J = 7.7 Hz, 2H), 7.21 – 7.20 (m, 2H), 7.17 – 7.10 (m, 3H), 7.03 – 7.00 (m, 2H), 6.92 (s, 2H) ppm. ^{31}P NMR ($CDCl_3$, 121 MHz, δ): 38.7 ppm. ^{13}C NMR ($CDCl_3$, 125 MHz, δ): 145.5 (dbr, J = 242.6 Hz), 142.6 (d, J = 25.5 Hz), 140.1 (d, J = 262.2 Hz), 136.7, 130.1 (d, J = 6.5 Hz), 129.2 (d, J = 4.5 Hz), 128.8 (d, J = 17.4 Hz), 128.7 (d, J = 4.5 Hz), 124.2, 122.3, 121.5, 118.0 (d, J = 10.1 Hz), 113.7, 112.0 (d, J = 19.2 Hz) ppm. HRMS: m/z = 487.0986 ($[M+H]^+$, Calcd. 487.0982).

BTD-P was obtained as a green solid (Yield: 78%). ^1H NMR ($CDCl_3$, 500 MHz, δ): 8.04 (d, J = 8.3 Hz, 2H), 7.99 (s, 2H), 7.65 (d, J = 7.8 Hz, 2H), 7.35 (t, J = 7.7 Hz, 2H), 7.24 – 7.21 (m, 2H), 7.00 – 6.96 (m, 3H), 6.91 – 6.88 (m, 2H) ppm. ^{31}P NMR ($CDCl_3$, 121 MHz, δ): 38.5 ppm. ^{13}C NMR ($CDCl_3$, 125 MHz, δ): 154.2, 143.2 (d, J = 26.1 Hz), 141.5 (d, J = 6.7 Hz), 136.3, 134.7, 129.6, 129.5, 129.4 (d, J = 4.5 Hz), 128.3 (d, J = 4.7 Hz), 123.9, 122.1, 121.5, 121.2, 112.1 (d, J =19.5 Hz), 111.4 ppm. HRMS: m/z = 473.0987 ($[M+H]^+$, Calcd. 473.0984).

CP-P was obtained as a white solid (Yield: 61%). ^1H NMR ($CDCl_3$, 300 MHz, δ): 8.01 – 8.02 (m, 2H), 7.60 (d, J = 7.8 Hz, 2H), 7.34 – 7.27 (m, 2H), 7.19 (t, J = 7.9, 2H), 7.15 – 7.12 (m, 2H), 7.07 – 7.02 (m, 2H), 6.70 (s, 2H), 6.31 – 6.28 (m, 2H), 2.99 – 2.93 (m, 2H), 2.48 – 2,43 (m, 2H), 1.92 – 1.85 (m, 1H), 0.86 – 0.84 (m, 1H) ppm. ^{31}P NMR ($CDCl_3$, 121 MHz, δ): 41.8 ppm. ^{13}C NMR ($CDCl_3$, 125 MHz, δ): 142.6 (d, J = 27.7), 140.9 (d, J = 7.3 Hz), 139.2 (d, J = 7.0 Hz), 129.9, 129.9 (d, J = 5.0), 129.1 (d, J = 1.5 Hz), 128.4 (d, J = 4.5 Hz), 128.2 (d, J = 17.2 Hz), 123.3 (d, J = 1.5 Hz), 121.9 (d, J = 1.5 Hz), 121.0 (d, J = 1.3 Hz), 112.2 (d, J = 20.2 Hz), 108.8, 37.4, 22.5 ppm. HRMS: m/z = 405.1508 ($[M+H]^+$, Calcd. 405.1521).

MI-P was obtained as a red solid (Yield: 85%). ^1H NMR ($CDCl_3$, 500 MHz, δ): 8.07 (d, J = 8.3 Hz, 2H), 7.83 (s, 2H), 7.74 (d, J = 7.9 Hz, 2H), 7.43 (t, J =7.7 Hz, 2H), 7.27 (t, J = 7.5, 2H), 7.17 – 7.14 (m, 1H), 7.09 – 7.06 (m, 2H), 6.31 – 6.28 (m, 2H), 3.00 (s, 3H) ppm. ^{31}P NMR ($CDCl_3$, 121 MHz, δ): 44.4 ppm. ^{13}C NMR ($CDCl_3$, 125 MHz, δ): 169.6, 143.7 (d, J= 27.7 Hz), 137.4 (d, J= 7.56), 134.1 (d, J = 8.8 Hz), 130.0, 130.0, 129.2 (d, J = 5.0 Hz), 128.2 (d, J = 17.6 Hz), 126.0, 122.8 (d, J = 13.9 Hz), 117.3, 112.5, 112.4, 24.6 ppm. HRMS: m/z = 448.1215 ($[M+H]^+$, Calcd. 448.1208).

AN-P was obtained as a red solid (Yield: 61%). ^1H NMR ($CDCl_3$, 500 MHz, δ): 8.13 (d, J = 8.2 Hz, 2H), 7.93 (d, J = 6.9 Hz, 2H), 7.75 (d, J = 8.1 Hz, 2H), 7.71 (d, J = 7.8 Hz, 2H), 7.57 – 7.48 (m, 2H), 7.36 (d, J = 7.6 Hz, 2H), 7.26 (t, J = 7.5 Hz, 2H), 7.22 (s, 2H), 6.82 – 6.76 (m, 3H), 6.71 – 6.68 (m, 2H) ppm. ^{31}P NMR ($CDCl_3$, 121 MHz, δ): 40.30 ppm. ^{13}C NMR ($CDCl_3$, 125 MHz, δ): 142.5 (d, J = 27.7 Hz), 138.7, 137.6 (d, J = 7.5 Hz), 137.5, 130 (d, J = 5.0 Hz), 129.7, 129.3, 129.0, 129.7, 129.3, 129.0, 128.7, 128.5, 128.3 (d, J = 3.8 Hz), 128.1, 127.8, 124.3, 123.6, 122.1, 121.2, 112.2, 112.1, 110.4 ppm. HRMS: m/z = 489.1516 ($[M+H]^+$, Calcd. 489.1515).

General Synthesis of ***B***- and ***O*-PO**s: In a 1-necked 150-mL Schlenk flask, the corresponding ***B***- or ***O*-P** (1.0 mole eq.) was mixed with H_2O_2 (5.0 mole eq.) in dichloromethane. The resulting mixture was stirred 2 days at room temperature. The reaction mixture was poured into 10 mL of water. The organic layer was extracted with chloroform. The crude product was purified by flash chromatography using dichloromethane and hexanes as eluent (from 1:9 to 9:1 by volume) to obtain the corresponding ***B***- and ***O*-PO**.

BZ-PO was obtained as a white solid (Yield: 87%). ^1H NMR ($CDCl_3$, 500 MHz, δ): 8.68 (d, J = 8.4 Hz, 2H), 7.61 (dd, J = 7.8, J = 1.7 Hz, 2H), 7.40 – 7.36 (m, 2H), 7.33 (ddd, J = 8.5, J = 7.2, J = 1.4 Hz 2H), 7.28 – 7.24 (m, 4H), 7.22 – 7.21 (m, 9H), 7.16 – 7.13 (m, 9H), 7.12 – 7.08 (m, 9H), 6.82 (d, J = 3.0 Hz, 2H) ppm. ^{31}P NMR ($CDCl_3$, 121 MHz, δ): 15.1 ppm. ^{13}C NMR ($CDCl_3$, 125 MHz, δ): 141.5 (d, J = 3.9 Hz), 139.5 (d, J = 6.0 Hz), 133.2 (d, J = 3.1 Hz), 132.2, 130.9, 130.8, 130.8, 130.7, 130.2 (d, J = 8.82 Hz), 128.5 (d, J = 15.8 Hz), 124.6, 123.1, 120.8, 115.9, 110.1 (d, J = 6.9 Hz) ppm. HRMS: m/z = 431.1307 ($[M+H]^+$, Calcd. 431.1308).

FBZ-PO was obtained as a white solid (Yield: 85%). ^1H NMR ($CDCl_3$, 500 MHz, δ): 8.63 (d, J = 8.5 Hz), 7.62 (d, J = 7.9 Hz, 2H), 7.44 – 7.35 (m, 5H), 7.28 – 7.25 (m, 4H), 6.92 (s, 2H) ppm. ^{31}P NMR ($CDCl_3$, 121 MHz, δ): 13.9 ppm. ^{13}C NMR ($CDCl_3$, 125 MHz, δ): 141.2 (d, J = 3.78 Hz), 134.05 (d, J = 2.52 Hz), 131.9, 130.6 (d, J = 1.26 Hz), 129.6 (d, J = 8.82 Hz), 129.0 (d, J = 16.4 Hz), 126.8 (d, J = 5.04 Hz), 125.6, 123.5, 121.4, 115.9, 114.5 ppm. HRMS: m/z = 503.0929 ($[M+H]^+$, Calcd. 503.0931).

BTD-PO was obtained as a green solid (Yield: 90%). ^1H NMR ($CDCl_3$, 500 MHz, δ): 8.67 (d, J = 8.2 Hz, 2H), 7.99 (s, 2H), 7.64 (d, J = 7.8 Hz, 2H), 7.40 – 7.36 (m, 4H), 7.29 – 7.26 (m, 2H), 7.19 – 7.16 (m, 1H), 7.07 – 7.05 (m, 2H), 6.98 (d, J = 2.6 Hz, 2H) ppm. ^{31}P NMR ($CDCl_3$, 121 MHz, δ): 14.5 ppm. ^{13}C NMR ($CDCl_3$, 125

MHz, δ): 154.2, 142.2 (d, J = 3.7 Hz), 138.0 (d, J = 5.8 Hz), 133.82 (d, J = 3.2 Hz), 133.5, 131.3, 131.3, 130.9 (d, J = 162.5 Hz), 129.9 (d, J = 8.3 Hz), 128.8, 128.6, 125.4, 123.4, 122.1, 121.2, 116.0, 112.5 (d, J = 6.9 Hz) ppm. HRMS: m/z = 489.0934 ($[M+H]^+$, Calcd. 489.0933).

CP-PO was obtained as a white solid (Yield: 80%). ^{1}H NMR ($CDCl_3$, 500 MHz, δ): 8.70 (d, J = 8.3 Hz, 2H), 7.6 (d, J = 7.8 Hz, 2H), 6.71 (d, J = 2.5, 2H), 7.38 – 7.32 (m, 3H), 7.28 – 7.25 (m, 2H), 7.18 – 7.14 (m, 2H), 6.71 (d, J = 2.5 Hz, 2H), 6.69 – 6.64 (m, 2H), 3.05 – 2.99 (m, 2H), 2.56 – 2.51 (m, 2H), 1.98 – 1.91 (m, 1H), 0.83 – 0.78 (m, 1H) ppm. ^{31}P NMR ($CDCl_3$, 121 MHz, δ): 15.8 ppm. ^{13}C NMR ($CDCl_3$, 125 MHz, δ): 141.5 (d, J = 4.8 Hz), 136.5 (d, J = 5.9 Hz), 133.11 (d, J = 3.3 Hz), 132.3 (d, J = 158.4 Hz), 130.4 (d, J = 8.5 Hz), 129.6 (d, J = 11.9 Hz), 129.3, 128.8 (d, J = 15.6 Hz), 125.0, 123.4, 120.9, 116.4, 109.6 (d, J = 7.1 Hz), 37.8, 22.2 ppm. HRMS: m/z = 421.1463 ($[M+H]^+$, Calcd. 421.1470).

MI-PO was obtained as a red solid (Yield: 87%). ^{1}H NMR ($CDCl_3$, 500 MHz, δ): 8.73 (d, J = 8.5 Hz, 2H), 7.96 (d, J 2.5 Hz, 2H), 7.76 (d, J = 7.8 Hz, 2H), 7.51 – 7.45 (m, 2H), 7.47 – 7.37 (m, 2H), 7.35 (t, J = 7.5 Hz, 2H), 7.19 (td, J = 7.7, J = 4.2 Hz, 2H), 6.69 – 6.61 (m, 2H), 3.02 (s, 3H) ppm. ^{31}P NMR ($CDCl_3$, 121 MHz, δ): 15.0 ppm. ^{13}C NMR ($CDCl_3$, 125 MHz, δ): 169.0, 142.7 (d, J = 4.3 Hz), 134.1 (d, J = 3.2 Hz), 131.5, 130.6 (d, J = 8.3 Hz), 130.2 (d, J = 4.9 Hz), 129.7 (d, J = 12.2 Hz), 129.5 (d, J = 15.7 Hz), 127.7, 124.3 122.7, 121.4, 117.8 (d, J = 6.3 Hz), 116.6, 24.7 ppm. HRMS: m/z = 464.1154 ($[M+H]^+$, Calcd. 464.1158).

AN-PO was obtained as a red solid (Yield: 88%). ^{1}H NMR ($CDCl_3$, 500 MHz, δ): 8.80 (d, J = 8.80 Hz, 2H), 7.93 (d, J = 7.0 Hz, 2H), 7.78 (d, J = 8.10, 2H), 7.71 (d, J = 7.8 Hz, 2H), 7.59 – 7.53 (m, 2H), 7.39 (t, J = 8.3 Hz, 2H), 7.32 (t, J = 7.5 Hz, 2H), 7.21 (d, J = 2.7 Hz, 2H), 7.12 – 7.02 (m, 3H), 6.95 – 6.91 (m, 2H), ppm. ^{31}P NMR ($CDCl_3$, 121 MHz, δ): 15.7 ppm. ^{13}C NMR ($CDCl_3$, 126 MHz, δ): 141.3 (d, J = 5.04 Hz), 138.1, 134.0 (d, J = 6.3 Hz), 133.1 (d, J = 2.5 Hz), 132.2, 130.9, 130.7 (d, J = 8.8 Hz), 129.9 (d, J = 12.6 Hz), 129.0, 128,7 (d, J = 2.5 Hz), 128.6, 128.5, 128.2 (d, J = 10.8), 125.0, 124.5, 123.3, 121.0, 116.1, 110.6 (d, J = 7.5 Hz) ppm. HRMS: m/z = 505.1466 ($[M+H]^+$, Calcd. 505.1464).

General Synthesis of *B*- and *O*-PO-Rs: In a 1-necked 150-mL Schlenk flask, the appropriate phosphorus reagent (1.0 mole eq.) was added to an acetonitrile solution of ***B*-** or ***O*-In** (1.0 mole eq.) in the presence of triehtylamine (2.0 mole eq.) at 0 °C. The resulting mixture was refluxed under argon overnight. The reaction mixture was allowed to cool to room temperature. Acetonitrile was removed under vacuum. The crude product

was purified by flash chromatography using dichloromethane and hexanes as eluent (from 1:9 to 9:1 by volume) to obtain **B-** and ***O*-P-R**s.

MI-PO-C8 was synthesized with octylphosphonic dichloride as the phosphorus reagent. The final product was obtained as an orange solid (Yield: 66%). ^{1}H NMR ($CDCl_3$, 500 MHz, δ): 8.61 (dd, J = 8.5, 0.5 Hz, 2H), 8.02 (s, J = 2 Hz, 2H), 7.69 (d, J = 7.9 Hz, 2H), 7.43 – 7.38 (m, 2H), 7.30 – 7.25 (m, 2H), 3.20 (s, 3H), 2.07 – 2.00 (m, 2H), 1.05 – 0.93 (m, 9H), 0.86 – 0.81 (m, 3H), 0.78 (t, J = 7.3 Hz, 3H) ppm. ^{31}P NMR ($CDCl_3$, 121 MHz, δ): 28.8 ppm. ^{13}C NMR ($CDCl_3$, 125 MHz, δ): 169.3, 142.0 (d, J = 3.8 Hz), 130.6 (d, J = 7.5 Hz), 129.0 (d, J = 5.0 Hz), 127.6, 124.0, 122.6, 120.9, 118.4 (d, J = 6.3 Hz), 116.4, 31.8, 30.97, 30.0, 29.9 (d, J = 15.7 Hz), 28.9 (d, J = 3.8 Hz), 24.9, 22.8, 20.8 (d, J = 5.0 Hz), 14.3 ppm. HRMS: m/z = 500.2095 ($[M+H]^+$, Calcd. 500.2103).

AN-PO-C8 was synthesized with octylphosphonic dichloride as the phosphorus reagent. The final product was obtained as a red solid (Yield: 53%). ^{1}H NMR ($CDCl_3$, 300 MHz, δ): 8.56 (d, J = 6.0 Hz, 2H), 8.36 (s, 2H), 8.12 (d, J = 7.0 Hz, 2H), 7.95 (d, J = 9.0 Hz, 2H), 7.34 – 7.29 (m, 2H), 7.25 – 7.20 (m, 2H), 7.01 (d = 2.4 Hz, 2H), 2.14 – 2.03 (m, 2H), 1.12 – 1.03 (m, 4H), 1.00 – 0.83 (m, 8H), 0.75 (t, d = 7.2 Hz, 3H) ppm. ^{31}P NMR ($CDCl_3$, 121 MHz, δ): 29.4 ppm. ^{13}C NMR ($CDCl_3$, 125 MHz, δ): 140.6 (d, J = 3.8 Hz), 133.0 (d, J = 6.0 Hz), 130.6 (d, J = 8.0 Hz), 129.4, 129.2, 128.7, 128.5, 128.3, 125.3, 125.0, 123.1, 120.9, 116.0, 111.0 (d, J = 6.6 Hz), 31.7, 31.2 (d, J = 109.6 Hz), 30.0, 29.6 (d, J = 16.4 Hz), 28.8 (d, J = 15.1 Hz), 22.7, 21.2 (d, J = 5.0 Hz), 14.3 ppm. HRMS: m/z = 541.2404 ($[M+H]^+$, Calcd. 541.2408).

BT-PO-C8 was synthesized with octylphosphonic dichloride as the phosphorus reagent. The final product was obtained as an green solid (Yield: 71%). ^{1}H NMR ($CDCl_3$, 500 MHz, δ): 8.55 (d, J = 10.0 Hz, 2H), 8.36(s, 2H), 7.59 (d, J = 7.8 Hz, 2H), 7.33 – 7.29 (m, 2H), 7.24 – 7.21 (m, 2H), 7.01 (d, J = 2.4 Hz, 2H), 2.11 – 2.05 (m, 2H), 1.10 – 1.04 (m, 4H), 0.98 – 0.92 (m, 4H), 0.92 – 0.80 (m, 4H), 0.75 (t, d = 14.6 Hz, 3H) ppm. ^{31}P NMR ($CDCl_3$, 121 MHz, δ): 27.6 ppm. ^{13}C NMR ($CDCl_3$, 126 MHz, δ): 154.8, 141.4 (d, J = 3.0 Hz), 136.8 (d, J = 5.8 Hz), 129.7 (d, J = 7.6 Hz), 125.3, 123.2, 122.8, 121.1, 115.8, 112.6 (d, J = 6.6 Hz), 116.4, 32.9 (d, J = 110.4 Hz), 31.8, 29.8 (d, J = 16.1 Hz), 29.0, 28.9, 22.7, 21.4 (d, J = 4.8 Hz) ppm. HRMS: m/z = 525.1867 ($[M+H]^+$, Calcd. 525.1878).

MI-POH was synthesized with phosphorus trichloride as the phosphorus reagent. The reaction was quenched with water. The final product was obtained as an orange solid (Yield: 82%). ^{1}H NMR ($CDCl_3$, 500 MHz, δ):

8.49 (d, J = 656.6 Hz, 1H), 8.42 (dd, J = 8.5, J = 1.0 Hz, 2H), 7.91 (d, J = 2.8, 2H), 7.71 (d, J = 7.9, 2H), 7.44 (ddd, J = 8.5, J = 7.2, J = 1.3 Hz, 2H), 7.31 (ddd, J = 7.9, J = 7.1, J = 1.0 Hz, 2H), 3.21 (s, 3H) ppm. ^{31}P NMR (C_6D_6, 203 MHz, δ): -1.75 (d, J = 665.7 Hz) ppm. ^{13}C NMR ($CDCl_3$, 125 MHz, δ): 168.9, 140.7 (d, J = 4.6 Hz), 130.9 (J = 7.5 Hz), 128.3 (J = 7.5 Hz), 127.4, 122.9, 121.4, 118.1 (J = 6.3 Hz), 115.6 ppm. Due to the instability of this compound, HRMS could not be obtained by using ESI experimental conditions.

Di-MI-P was synthesized with 1,2-bis(dichlorophosphino)ethane as the phosphorus reagent. The final product was obtained as a red solid (Yield: 64%). ^{1}H NMR ($CDCl_3$, 500 MHz, δ): 7.82 (s, 4H), 7.71 (d, J = 8.4 Hz, 4H), 7.57 (d, J = 7.9 Hz, 4H), 7.36 – 7.32 (m, 4H), 7.18 – 7.15 (m, 4H), 3.52 (d, J = 7.2 Hz, 2H), 1.82 – 1.76 (m, 2H), 1.60 (t, J = 10.1, 4H), 1.37 – 1.29 (m, 16H), 0.93 (t, J = 7.4 Hz, 6H), 0.88 (t, J = 6.8 Hz, 6H) ppm. ^{31}P NMR ($CDCl_3$, 121 MHz, δ): 50.1 ppm. ^{13}C NMR ($CDCl_3$, 125 MHz, δ): 169.5, 142.7 (t, J = 13.8 Hz), 1433.5 (t, J = 4.1 Hz), 129.8 (t, J = 2.52 Hz), 126.1, 122.8, 122.5, 121.5, 118.0 (t, J = 1.2 Hz), 112.4 (t, J = 11.3 Hz), 42.7, 38.5, 30.8, 28.8, 24.4, 24.2, 23.3, 14.4, 10.7 ppm. HRMS: m/z = 987.3901 ($[M+Na]^+$, Calcd. 987.3892).

Di-MI-PO was synthesized by oxidation of **Di-MI-P** with H_2O_2 (5.0 mole eq.). The final product was obtained as a red solid (Yield: 61%). ^{1}H NMR ($CDCl_3$, 500 MHz, δ): 8.34 (d, J = 8.5 Hz, 4H), 7.99 (s, 4H), 7.58 (d, J = 7.8 Hz, 4H), 7.32 – 7.30 (m, 4H), 7.24 – 7.21 (m, 4H), 3.58 (d, J = 7.2 Hz, 2H), 1.85 – 1.81 (m, 2H), 1.73 (d, J = 3.5, 4H), 1.41 – 1.34 (m, 16H), 0.97 (t, J = 10.0 Hz, 6H), 0.92 (t, J = 10.0 Hz, 6H) ppm. ^{31}P NMR ($CDCl_3$, 121 MHz, δ): 23.5 ppm. ^{13}C NMR ($CDCl_3$, 125 MHz, δ): 168.6, 141.5, 130.4, 128.7, 127.9, 124.2, 119.8, 119.5, 116.1, 42.9, 38.6, 30.8, 30.0, 28.9, 24.2, 23.4, 14.4, 10.8 ppm. HRMS: m/z = 1019.3783 ($[M+Na]^+$, Calcd. 1019.3791).

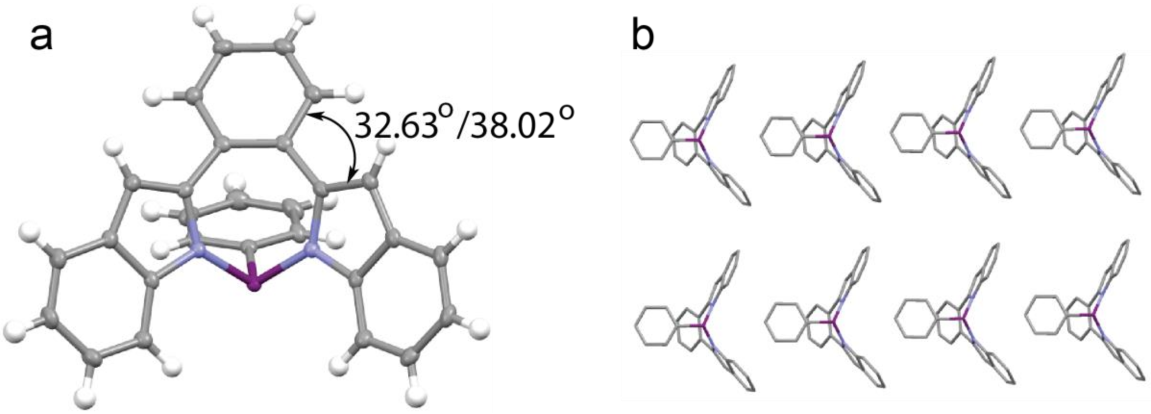

Figure S1. (a) The crystal structure of **BZ-P** and (b) its molecular stacking.

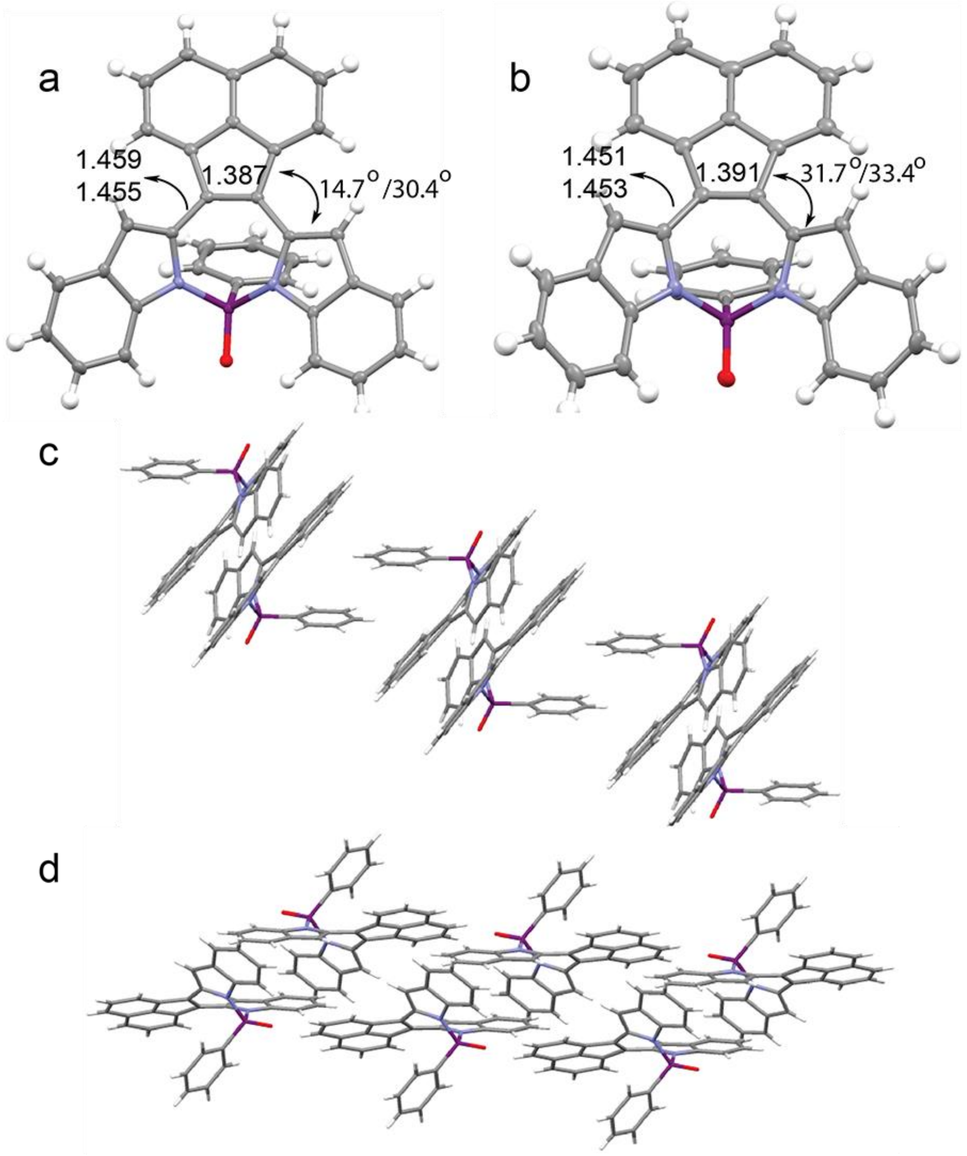

Figure S2. (a, b) The crystal structure of **AN-PO** and (c, d) its molecular stacking.

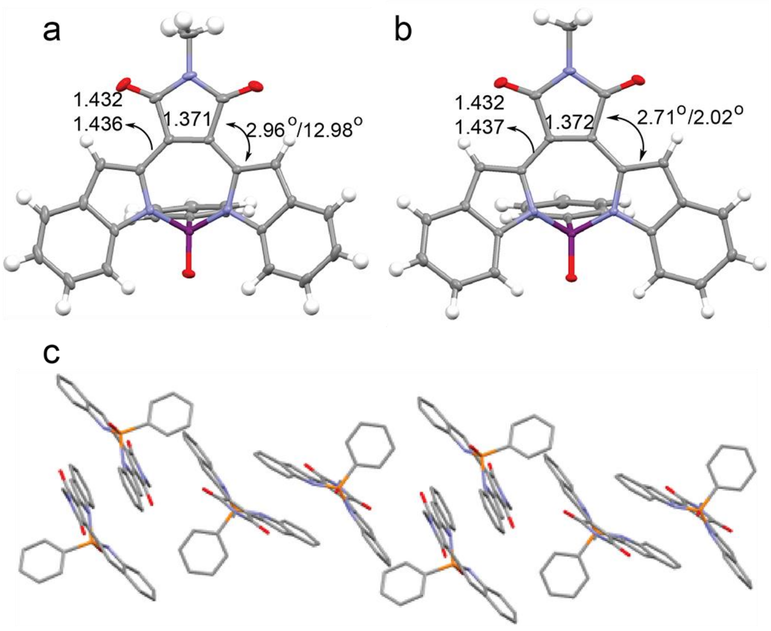

Figure S3. (a, b) The crystal structure of **MI-PO** and (c) its molecular stacking.

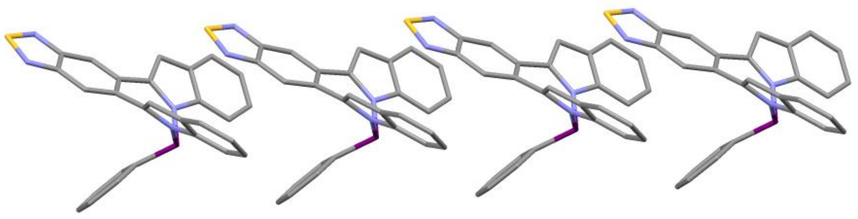

Figure S4. The molecular stacking of **BZ-P**.

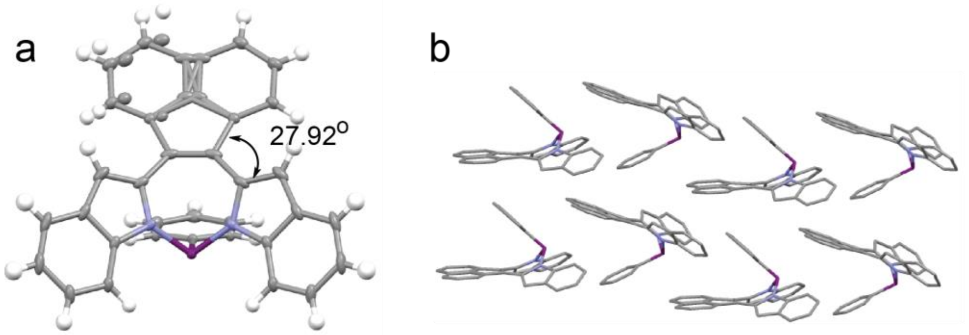

Figure S5. (a) The crystal structure of **AN-P** and (b) its molecular stacking.

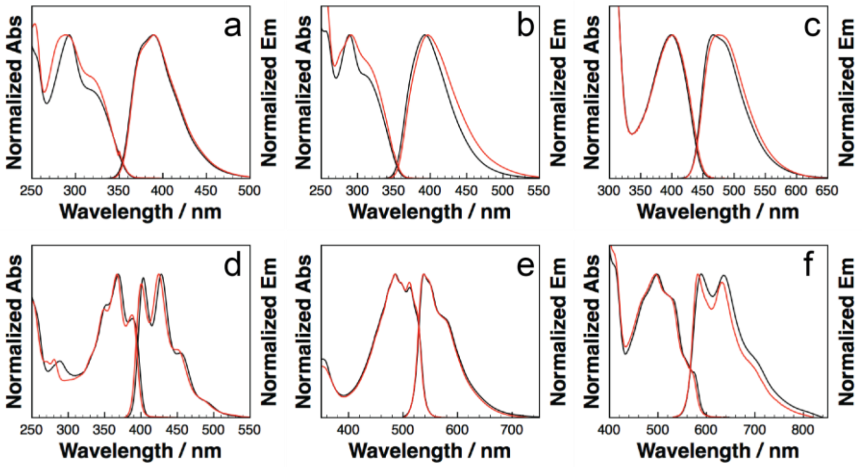

Figure S6. UV-vis absorption and emission spectra of ***B*****-** and ***O*****-P** derivatives (black), as well as of ***B*****-** and ***O*****-PO** derivatives (red) in hexane (ca. 10^{-5} M).

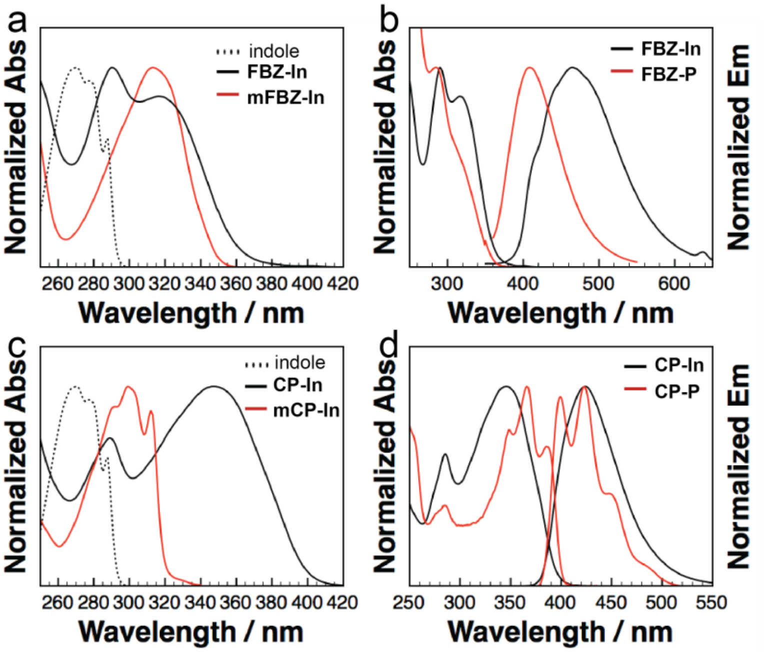

Figure S7. Comparison of UV-vis absorption and emission spectra of **FBZ** and **CP** derivatives.

The absence of its second indole leg in **CP-In** results in a substantial blue shift in the absorption spectrum of **mCP-In**; this blue shift is more substantial than that observed when comparing the absorption spectra of **FBZ-In** with **mFBz-In**. This observation further supports our hypothesis that derivatives with aromatic substitutions exhibit strong electronic confinement.

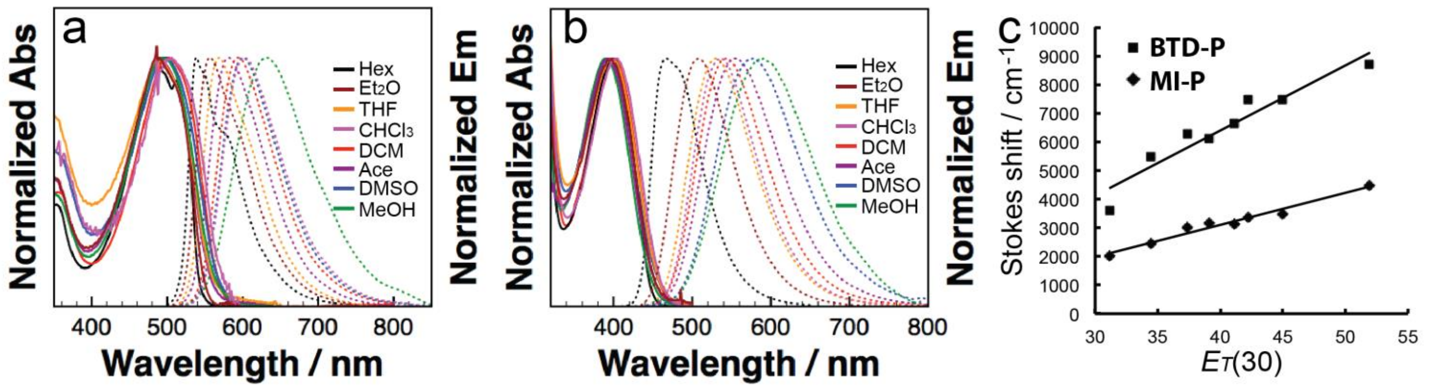

Figure S8. UV-vis absorption and emission spectra of (a) **MI-P** (a) and (b) **BTD-P** in different solvents (Hex: hexane, Et_2O: diethylether, THF: tetrahydrofuran, $CHCl_3$: chloroform, DCM: dichloromethane, Ace: acetone, DMSO: dimethyl sulfoxide, MeOH: methanol. Absorption data are represented by solid lines while emission data are represented by dotted lines. (c) Solvatochromic properties, as quantified by the Stokes shift as function of solvent polarity.

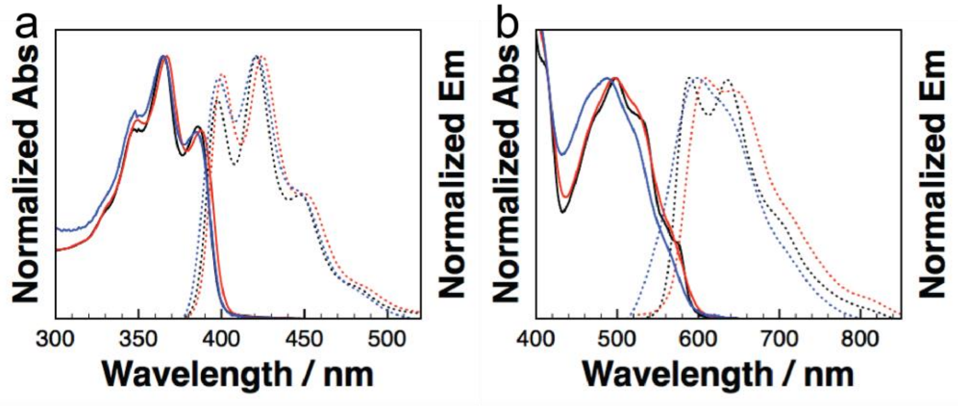

Figure S9. UV-vis absorption and emission spectra of (a) **CP-P** and (b) **AN-P** in different solvents (black: hexane, red: dichloromethane, blue: methanol). Absorption data are provided in solid lines while emission data are provided in dotted lines.

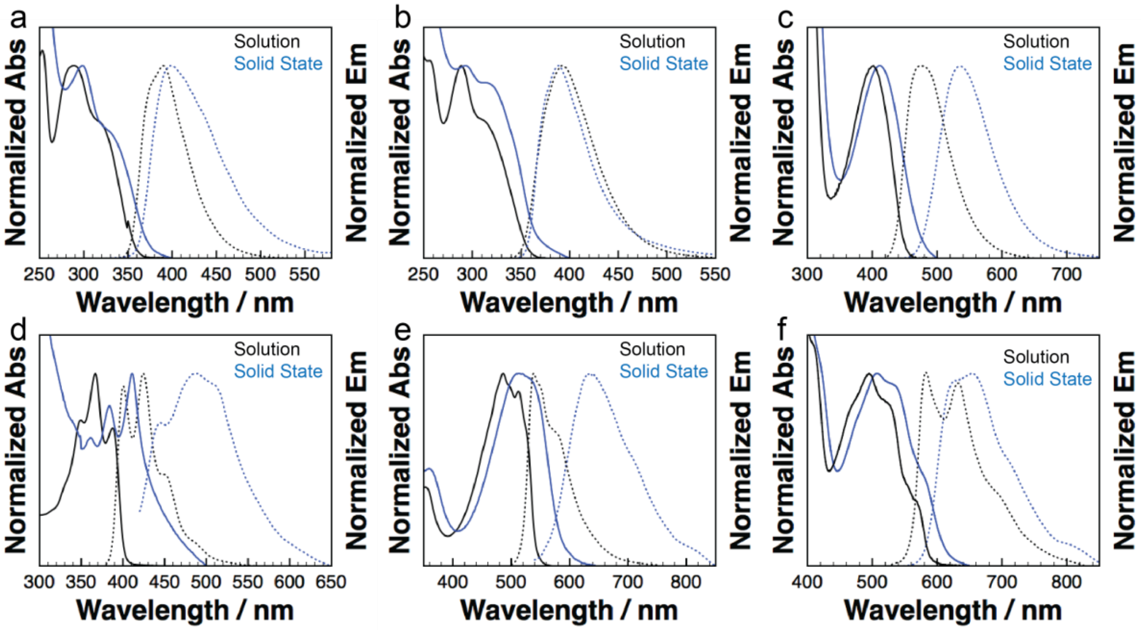

Figure S10. UV-vis absorption and emission spectra of (a) **BZ-P**, (b) **FBZ-P**, (c) **BTD-P**, (d) **CP-P**, (e) **MI-P**, (f) **AN-P** in solution and in the solid state. Absorption data are in solid lines while emission data are in dotted lines.

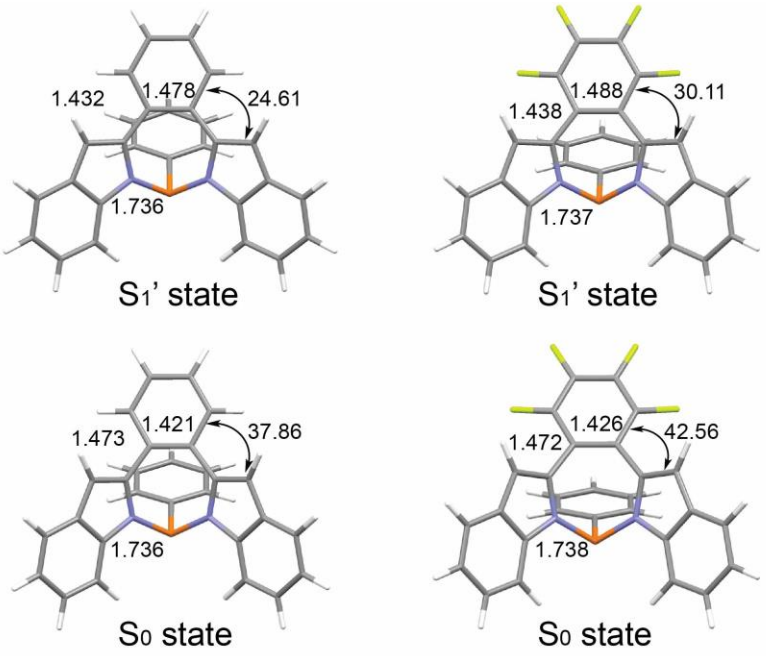

Figure S11. Optimized structure of **BZ-P** and **CP-P** at the S_0 and S_1' states at theory level of B3LYP/6-31+g(d) level.

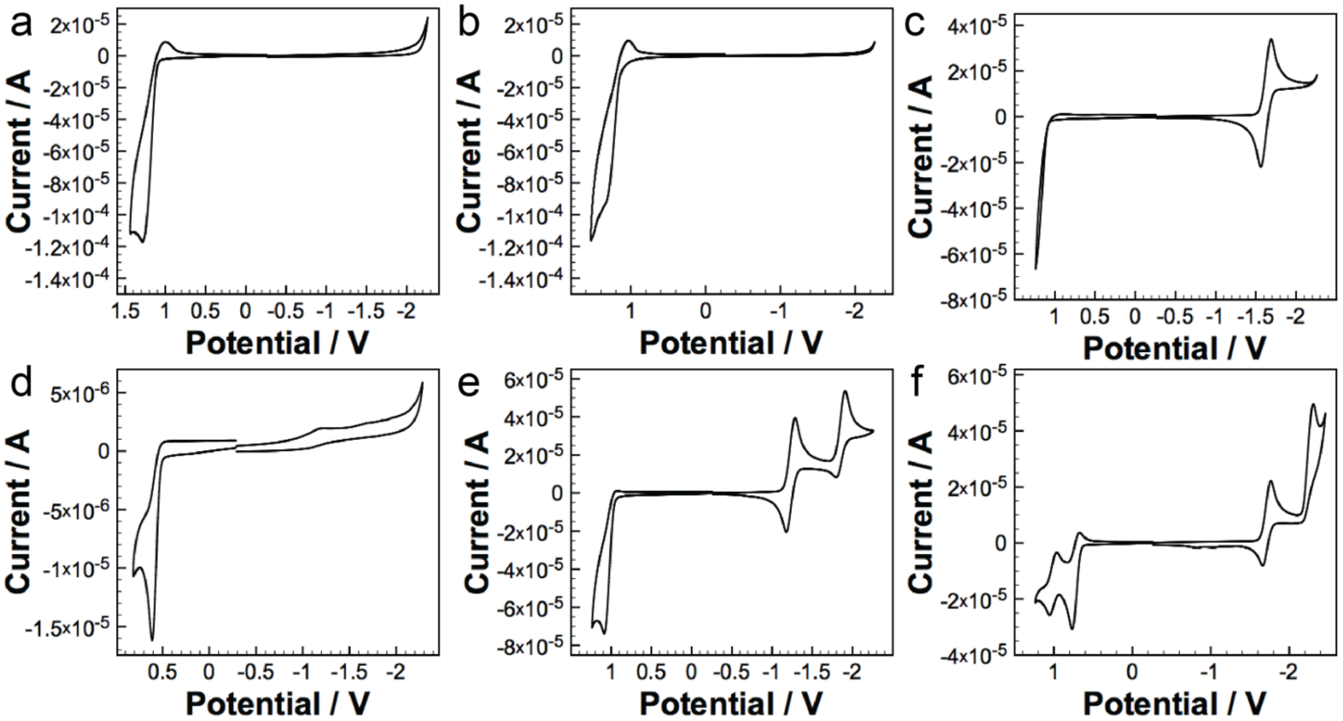

Figure S12. Cyclic voltammograms of ***B-*** and ***O*-PO** species: species (a) **BZ-PO**, (b) **FBZ-PO**, (c) **BTD-PO**, (d) **CP-PO**, (e) **MI-PO** and (f) **AN-PO** in DCM.

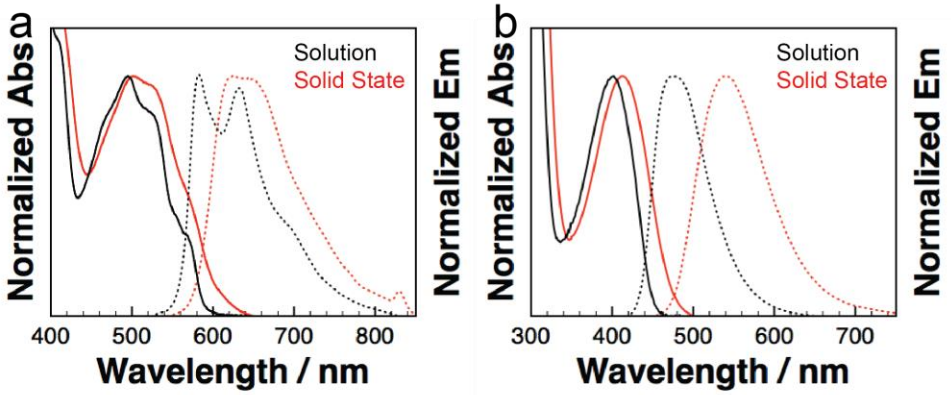

Figure S13. UV-vis absorption and emission spectra of (a) **BTD-PO-C8** and (b) **AN-PO-C8**. Absorption data are in solid lines while emission data are in dotted lines.

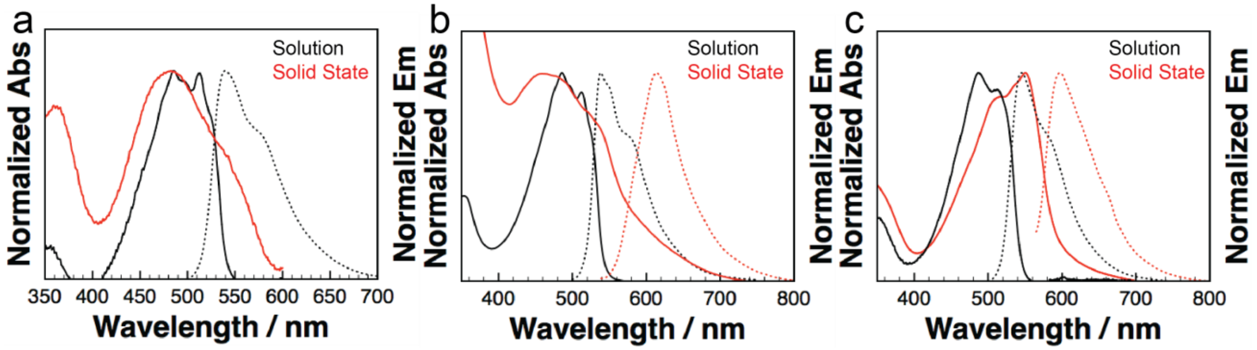

Figure S14. UV-vis absorption and emission spectra of (a) **MI-POH**, (b) **MI-PO-C8** and (c) **Di-MI-PO**. Absorption data are in solid lines while emission data are in dotted lines. Solution spectra were acquired in XX.

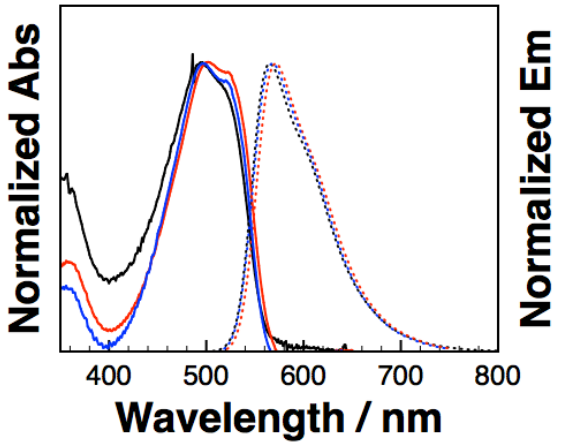

Figure S15. UV-vis absorption and emission spectra of (black) **MI-PO**, (red) **MI-PO-C8** and (blue) **Di-MI-PO** in toluene. Absorption data are in solid lines while emission data are in dotted lines.

Table S1: Photophysical and redox data of diazaphosphepine oxide species.

Compound	$\lambda_{abs}{}^{a}$ [nm]	$\lambda_{em}{}^{b}$ [nm]	ϕ^{c}	$E_{red}{}^{d}$	$E_{ox}{}^{d}$
**BZ-PO**	289	390	6.5%	n.d.^{e}	1.14^{f}
**FBZ-PO**	289	397	17.0%	n.d.^{e}	1.17^{f}
**BTD-PO**	402	474	21.6%	-1.62^{f}	n.d.^{e}
**CP-PO**	364	421	30.0%	n.d.^{e}	0.61^{g}
**MI-PO**	485	538	68.4%<br>72.5%^{h}<br>43.0%^{i}	-1.23^{f}/-1.94^{f}	1.00^{f}
**AN-PO**	495	582	18.7%	-1.71^{f}/-2.03^{g}	0.72^{f}/1.00^{f}
**BTD-PO-C8**	398	474	27.9%	N/A	N/A
**AN-PO-C8**	495	582	14.8%	N/A	N/A
**MI-PO-C8**	485	538	43.5%<br>59.4%^{h}<br>30.2%^{i}	N/A	N/A
**MI-POH**	486	539	27.6%	N/A	N/A
**Di-MI-P**	493	552	35.2%	N/A	N/A
**Di-MI-PO**	512	545	19.1%<br>44.3%^{h}<br>1.2%^{i}	N/A	N/A

^{a} absorption maximum measured in hexane. ^{b} emission maximum in hexane. ^{c} Fluorescence QY determined by a calibrated integrating sphere system in CH_2Cl_2. ^{d} vs. $Fc^{0/+}$, 0.1 M $Bu_4N[PF_6]$ in CH_2Cl_2. ^{e} not detected in solvent range. ^{f} quasi-reversible or reversible ($E_{red}(E_{ox}) = 1/2(E_{pc}+E_{pa})$). ^{g} irreversible ($E_{red}(E_{ox}) = E_{pc}(E_{pa})$). ^{h} in toluene. ^{I} in DMF.

The photophysical properties of **Di-MI-PO** are characteristically different from those of the other MI-derivatives, both in solution and in the solid state. Its fluorescence quantum yield decreases substantially with increasing solvent polarity whereas the fluorescence of **MI-PO-C8** and **MI-PO** do not (Table S1). We attribute the low quantum yield of **Di-MI-PO** in DMF, to the formation of a non-emissive symmetry-breaking charge transfer (SBCT) state in polar solvents. The presence of SBCT in dimeric systems has previously been reported.[S2-S4] The presence of SBCT in electron acceptors have been shown to improve the V_{oc} of organic solar cells, respectively.[S3]

Table S2: Nucleus-independent chemical shifts data at level of GIAO/B3LYP/6-31+g(d).

Compound	NICS(0)	NICS(-1Å)[a] ppm	NICS(+1Å)[b] ppm
BZ-P	3.86	0.72	0.20
FBZ-P	3.23	-0.54	0.55
BTD-P	4.12	0.09	1.04
CP-P	3.24	0.38	0.54
MI-P	2.46	-0.19	0.53
AN-P	2.66	-0.23	0.48

[a] H located at the same direction of Phenyl group on P-center.
[b] H located at the opposite direction of Phenyl group on P-center.

Table S3: Devices characteristics.

acceptor	blend ratio of donor and acceptor	V_{oc}[V]	J_{sc}[mA/cm^2]	*FF* [%]	*PCE* [%]
**Di-MI-PO**	1:1 wt as-cast	0.77±0.01	0.57±0.1	26±0.5	0.11±0.02
	1:1 wt annealed[a]	0.65±0.01	2.04±0.1	30±0.3	0.40±0.02
	1:2 wt annealed[a]	0.67±0.01	2.50±0.05	30±0.2	0.50±0.01
	1:4 wt annealed[a]	0.71±0.02	3.77±0.7	39±3	1.04±0.2
	1:5 wt annealed[a]	0.66±0.01	2.38±0.06	29±0.4	0.46±0.02
**MI-PO-C8**	1:1 wt annealed[b]	0.61±0.004	1.93±0.1	37±0.6	0.44±0.02
	1:2 wt annealed[b]	0.61±0.01	3.07±0.1	38±0.5	0.71±0.02
	1:4 wt annealed[b]	0.55±0.06	1.22±0.02	44±0.6	0.29±0.01

[a] annealed at 120C for 10 min. [b]annealed at 100C for 10 min,

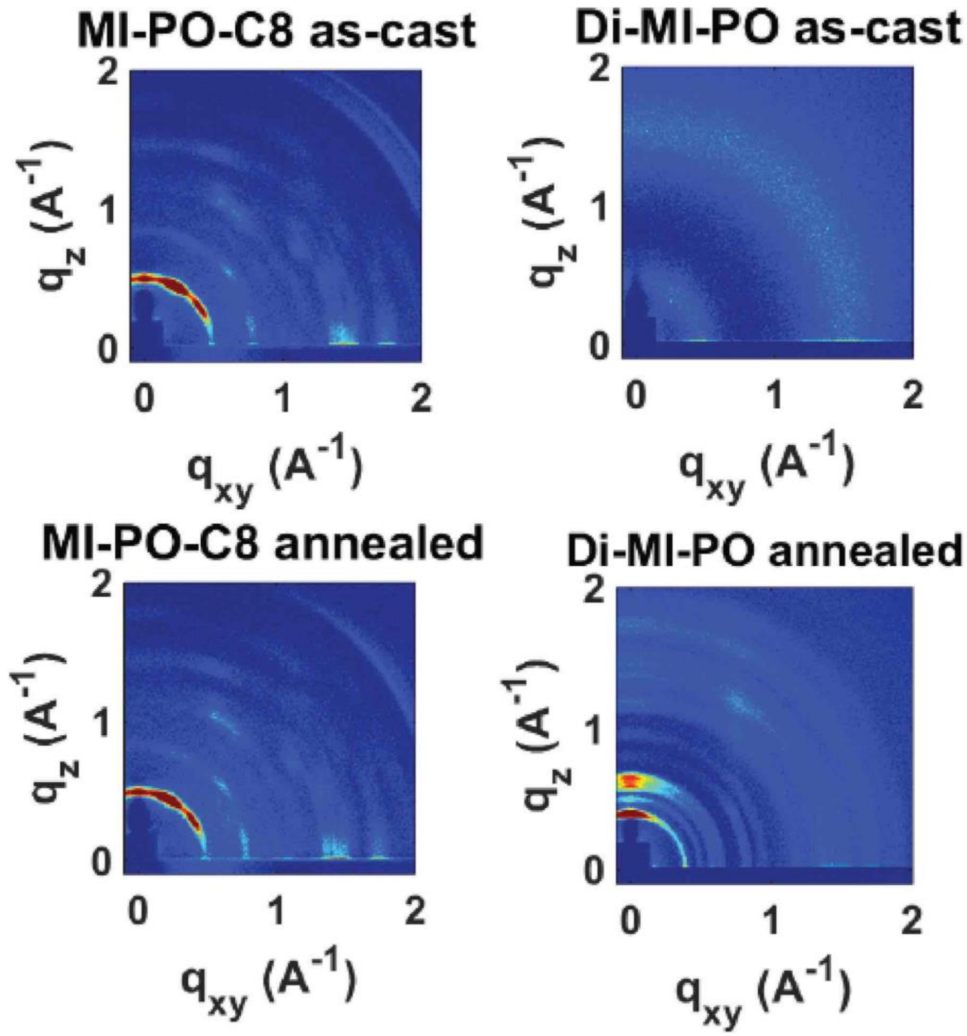

Figure 16. GIXD images of **Di-MI-C8** and **Di-MI-PO** as-cast and annealed films.

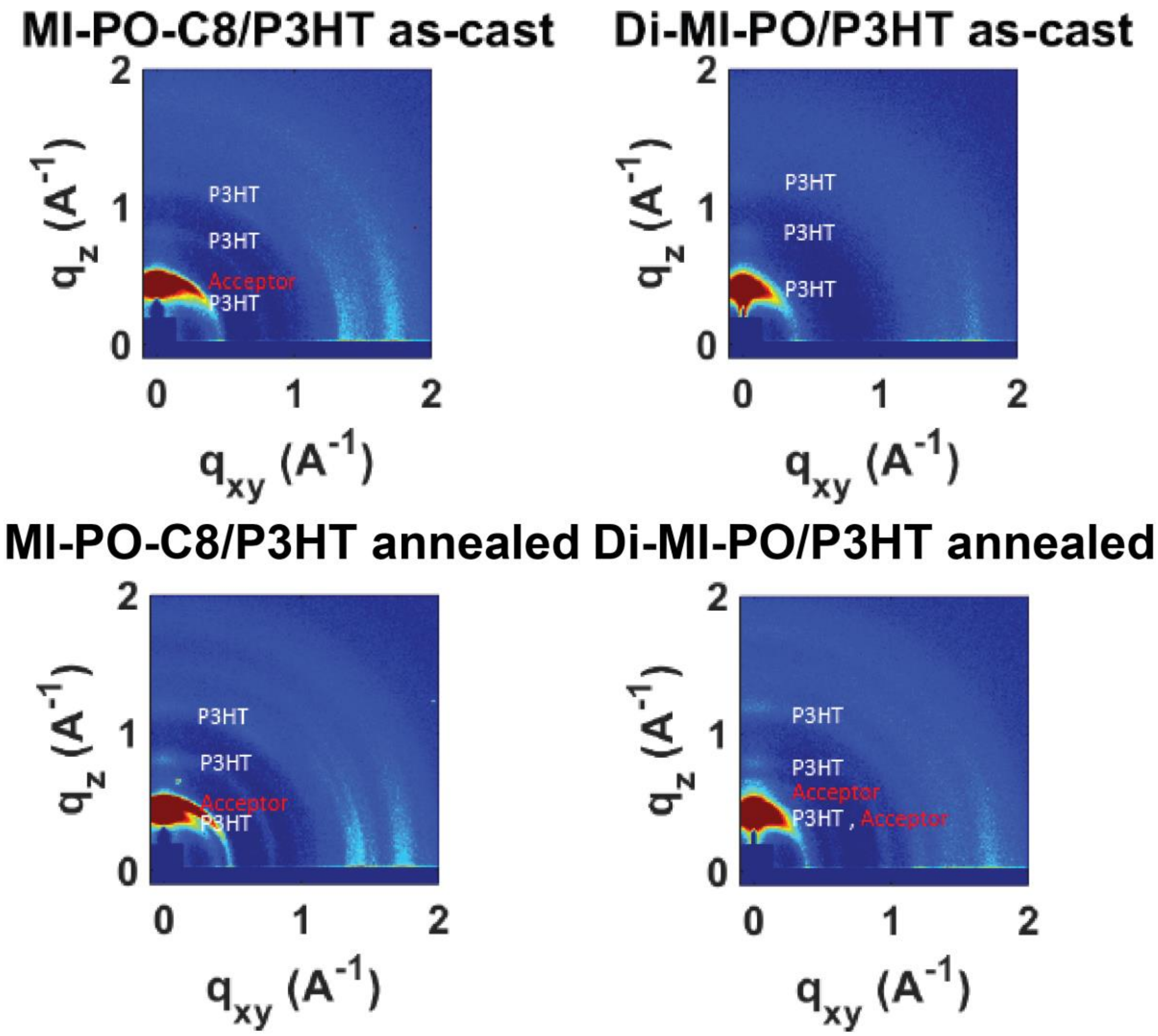

Figure 17. GIXD images of active layers comprising **Di-MI-C8** and P3HT as well as **Di-MI-PO** and P3HT before and after thermal annealing.

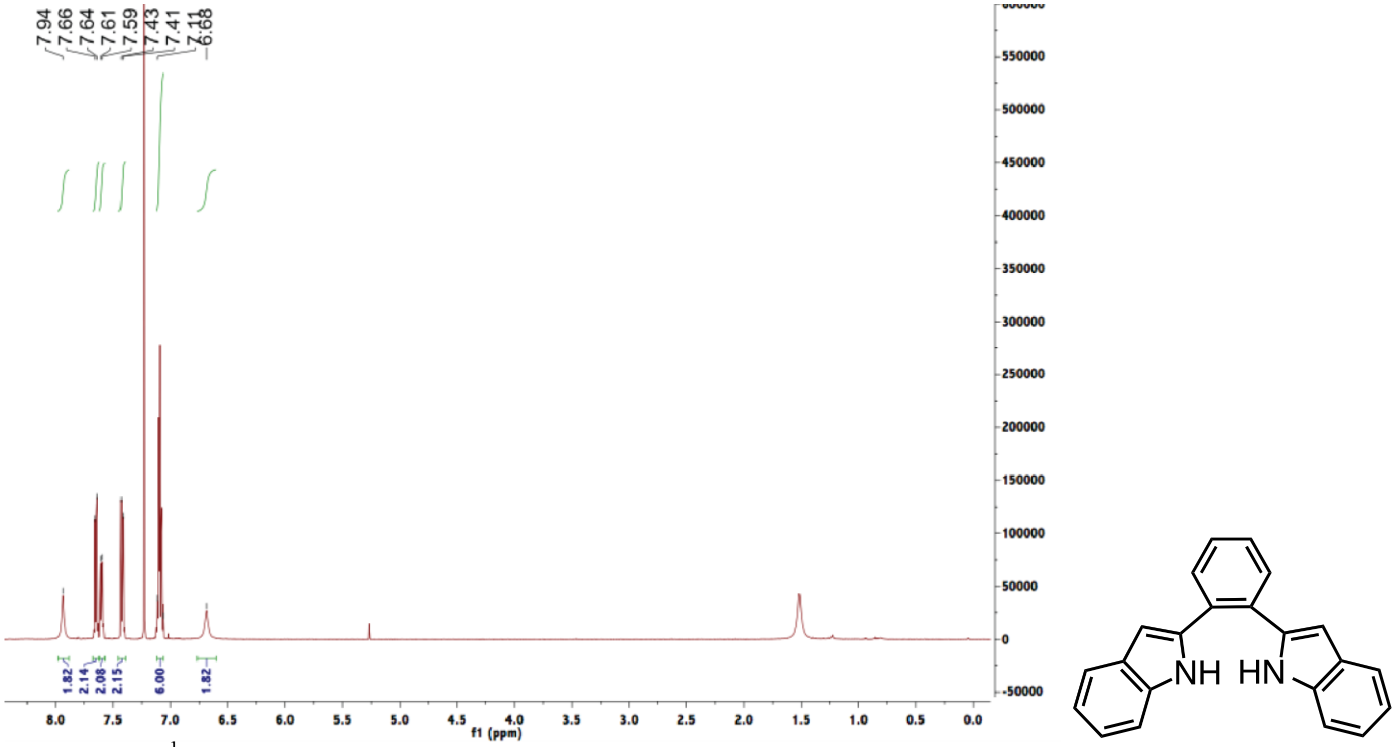

Figure S18. ^{1}H NMR spectrum of **BZ-In** in $CDCl_3$.

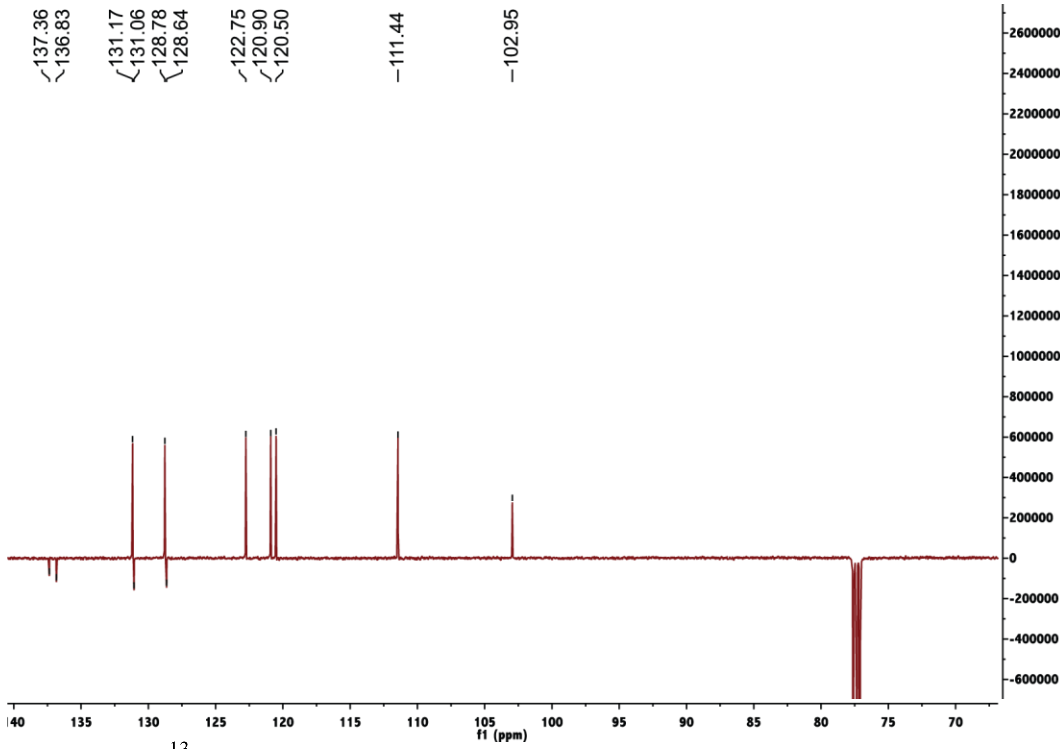

Figure S19. ^{13}C NMR spectrum of **BZ-In** in $CDCl_3$.

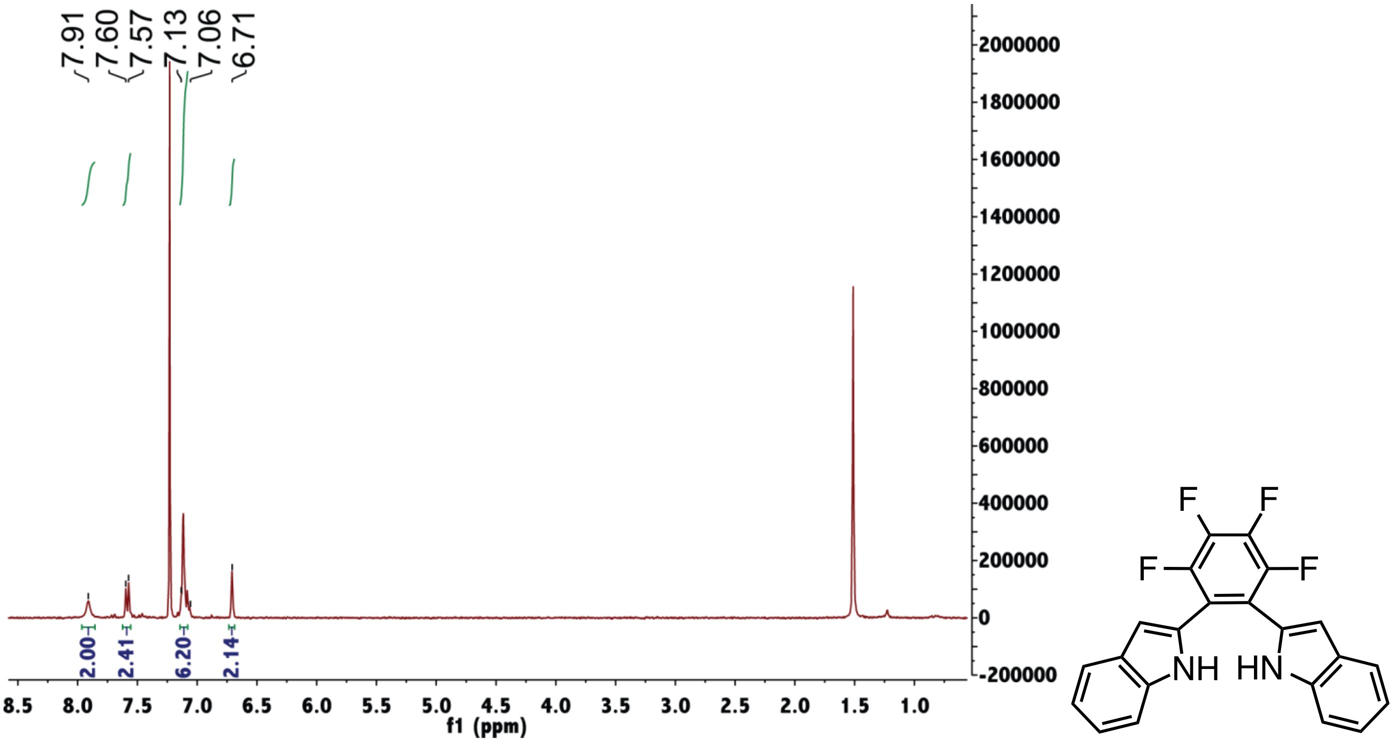

Figure S20. ^{1}H NMR spectrum of **FBZ-In** in $CDCl_3$.

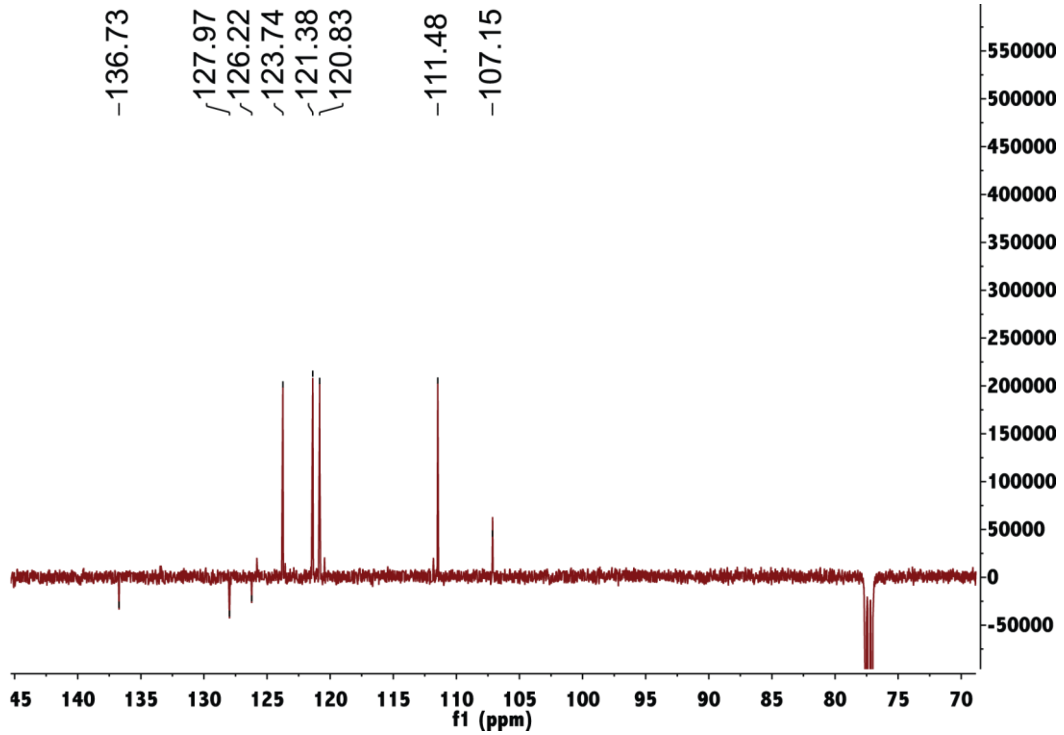

Figure S21. ^{13}C NMR spectrum of **FBZ-In** in $CDCl_3$.

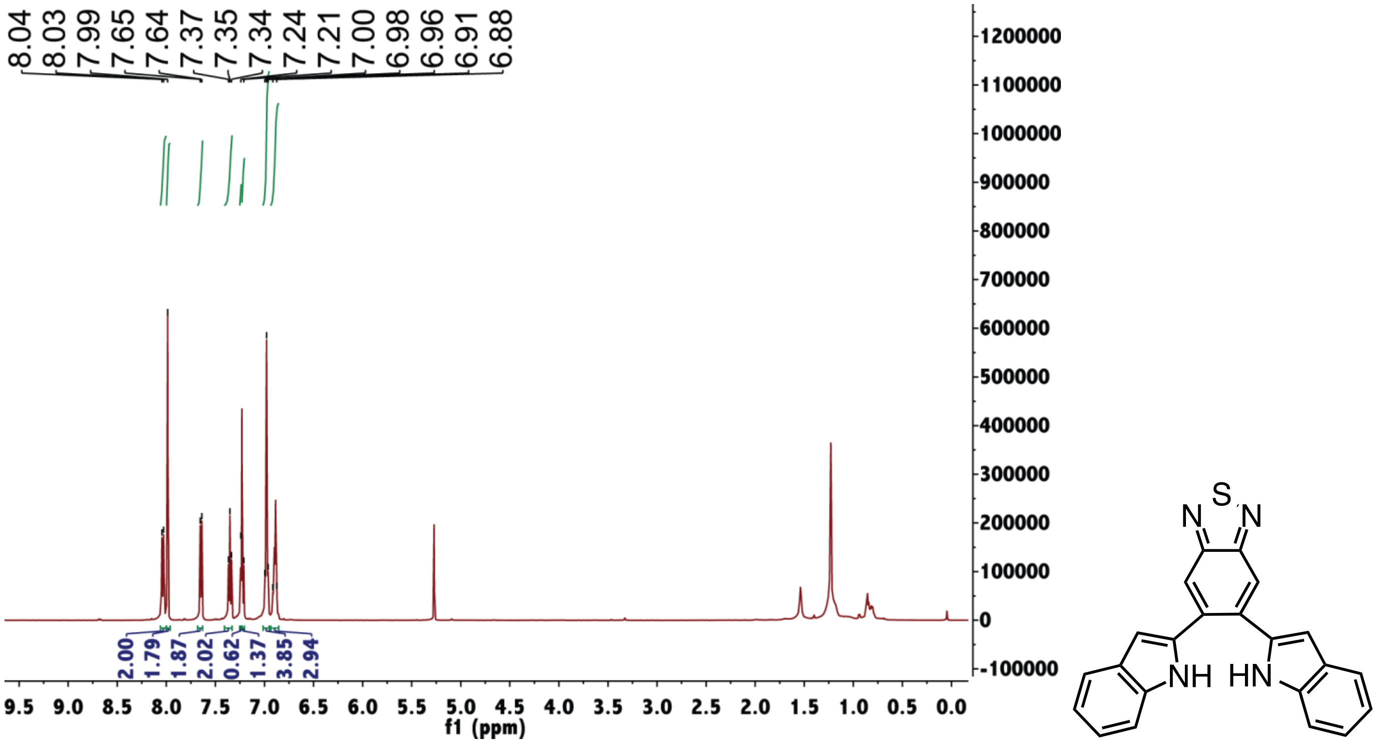

Figure S22. ^{1}H NMR spectrum of **BTD-In** in $CDCl_3$.

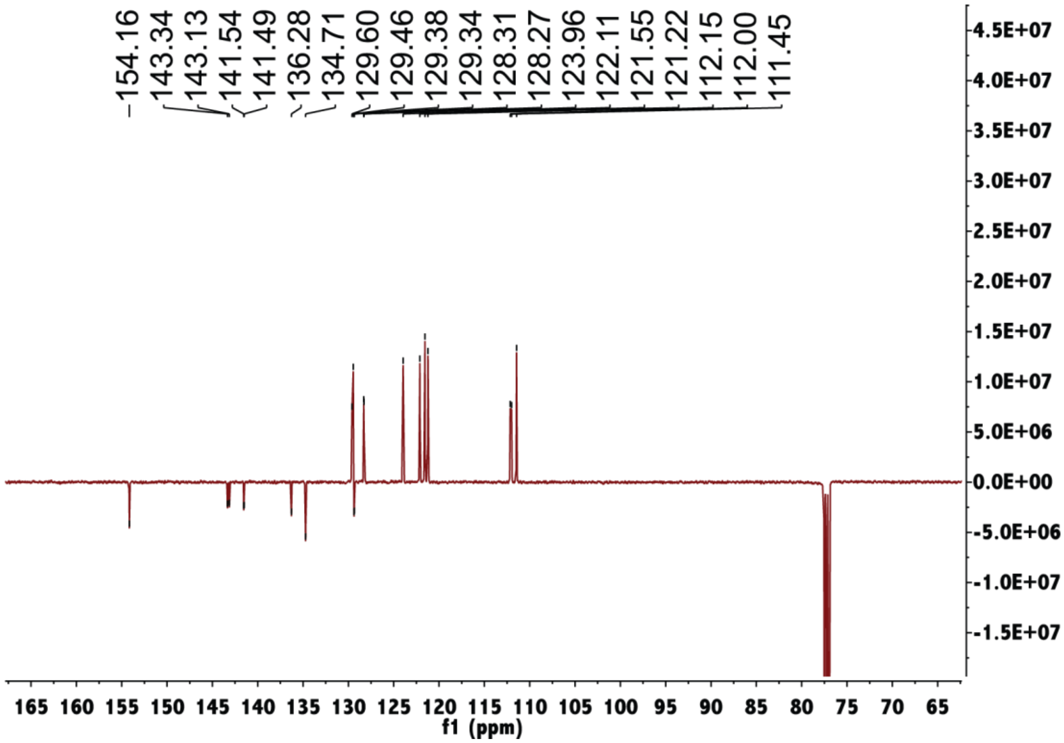

Figure S23. ^{13}C NMR spectrum of **BTD-In** in $CDCl_3$.

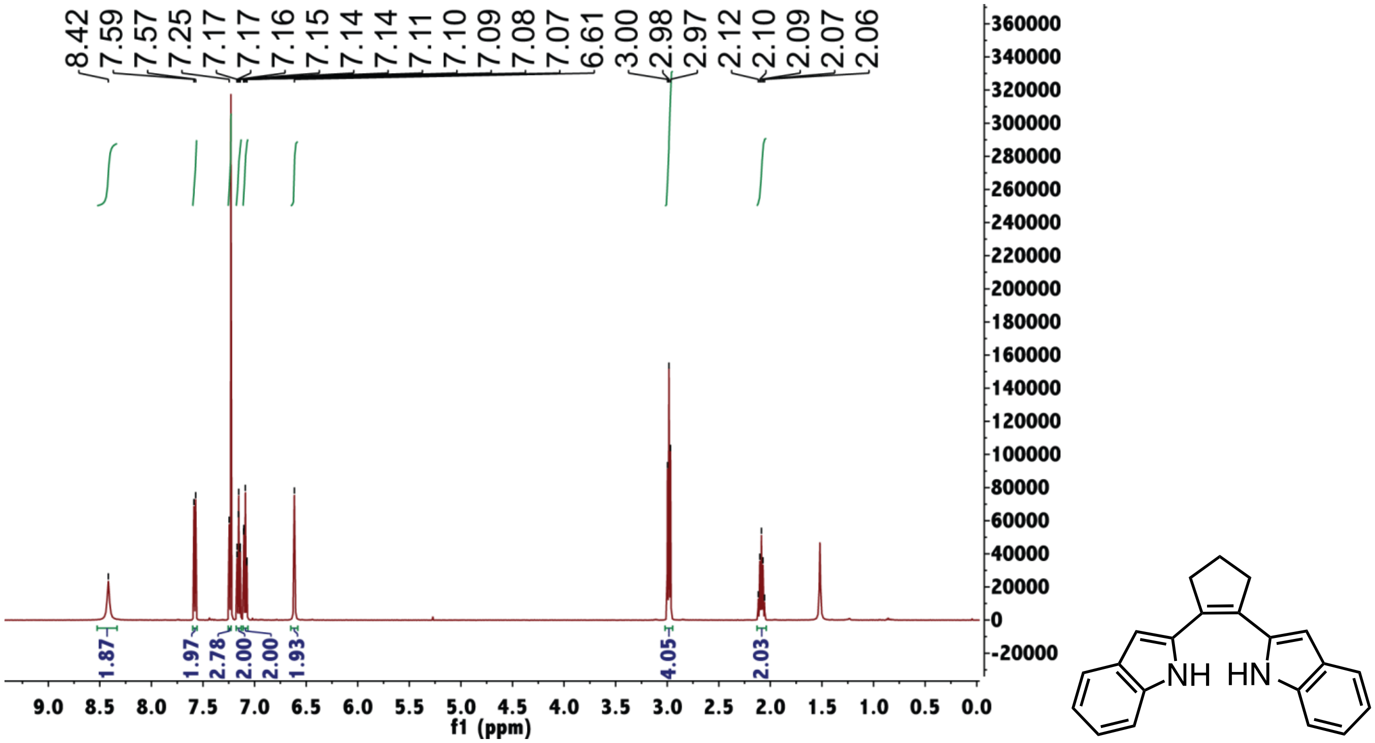

Figure S24. ^{1}H NMR spectrum of **CP-In** in $CDCl_3$.

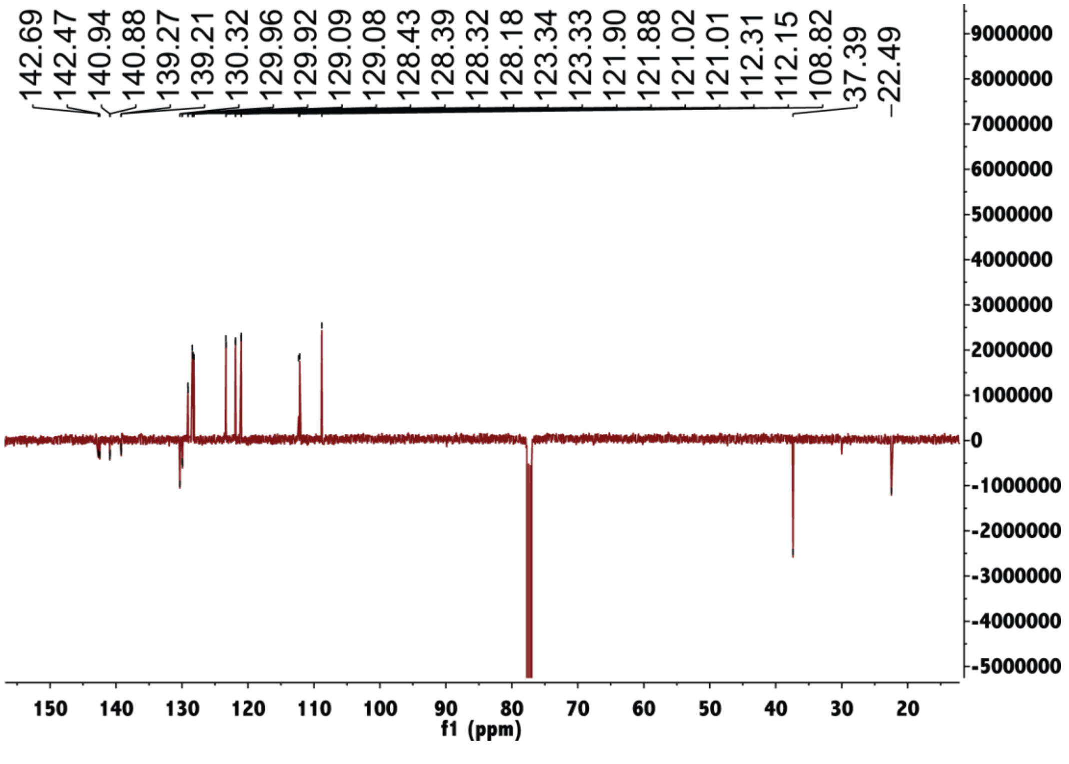

Figure S25. ^{13}C NMR spectrum of **CP-In** in $CDCl_3$.

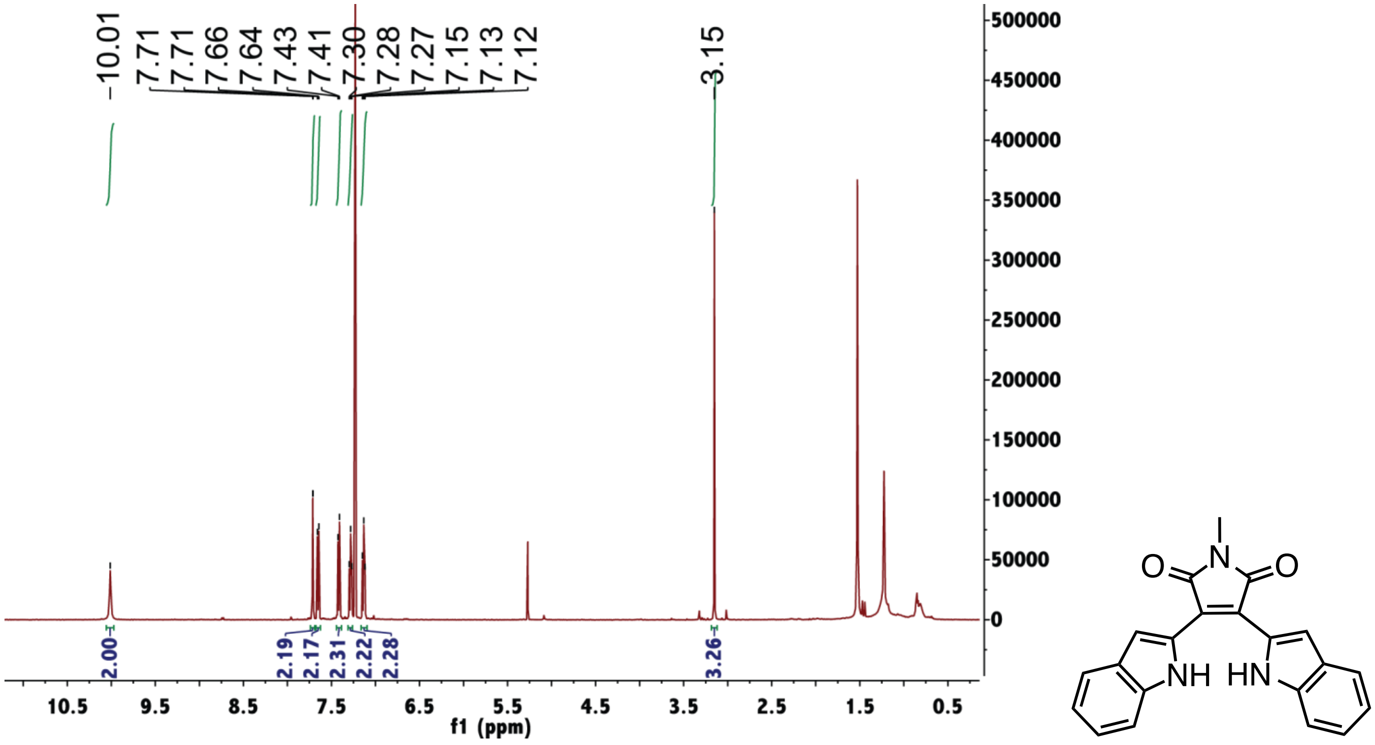

Figure S26. ^{1}H NMR spectrum of **MI-In** in $CDCl_3$.

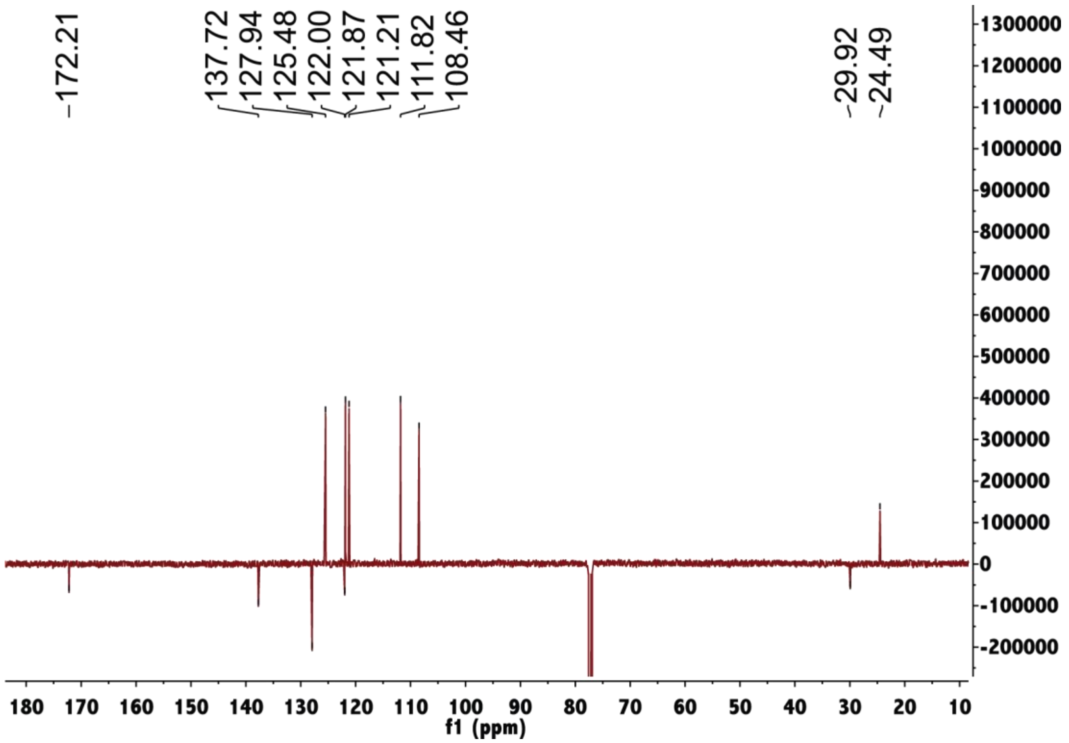

Figure S27. ^{13}C NMR spectrum of **MI-In** in $CDCl_3$.

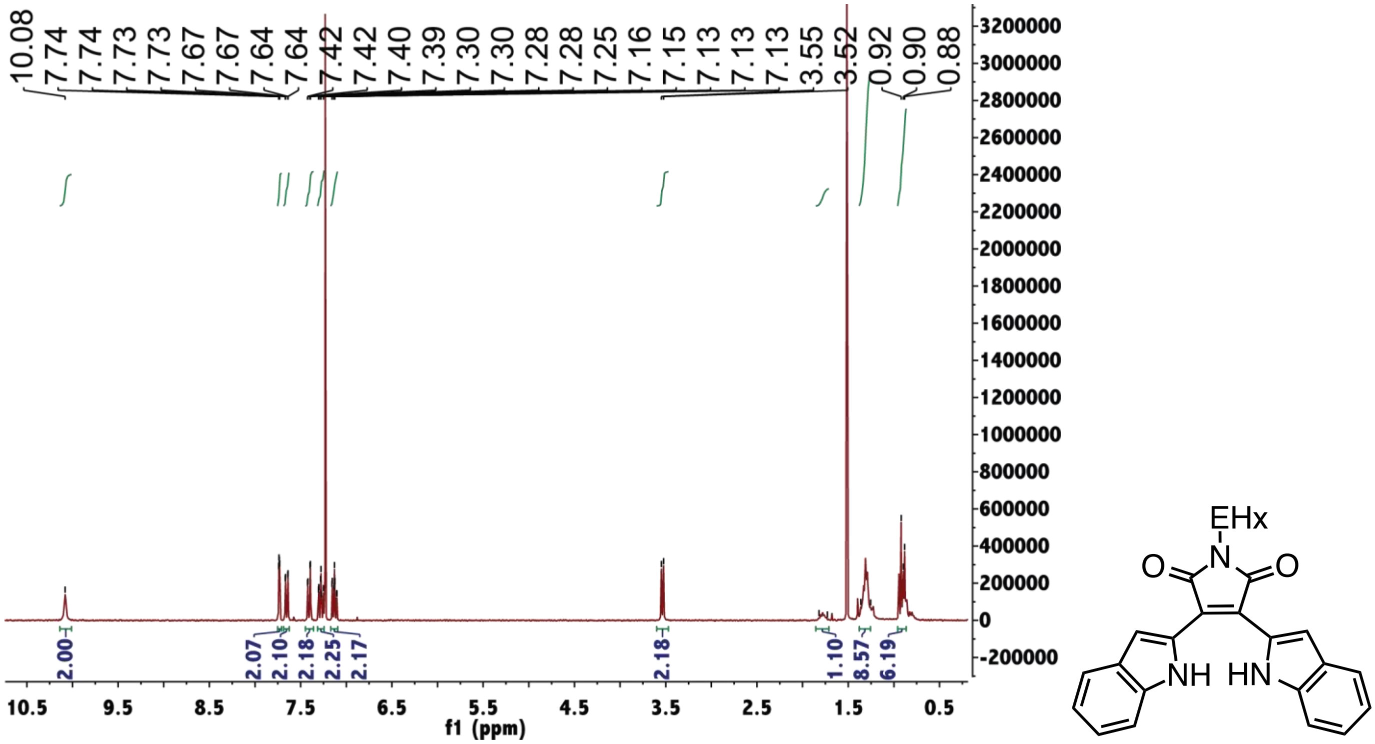

Figure S28. ^{1}H NMR spectrum of **MI-In-EHx** in $CDCl_3$.

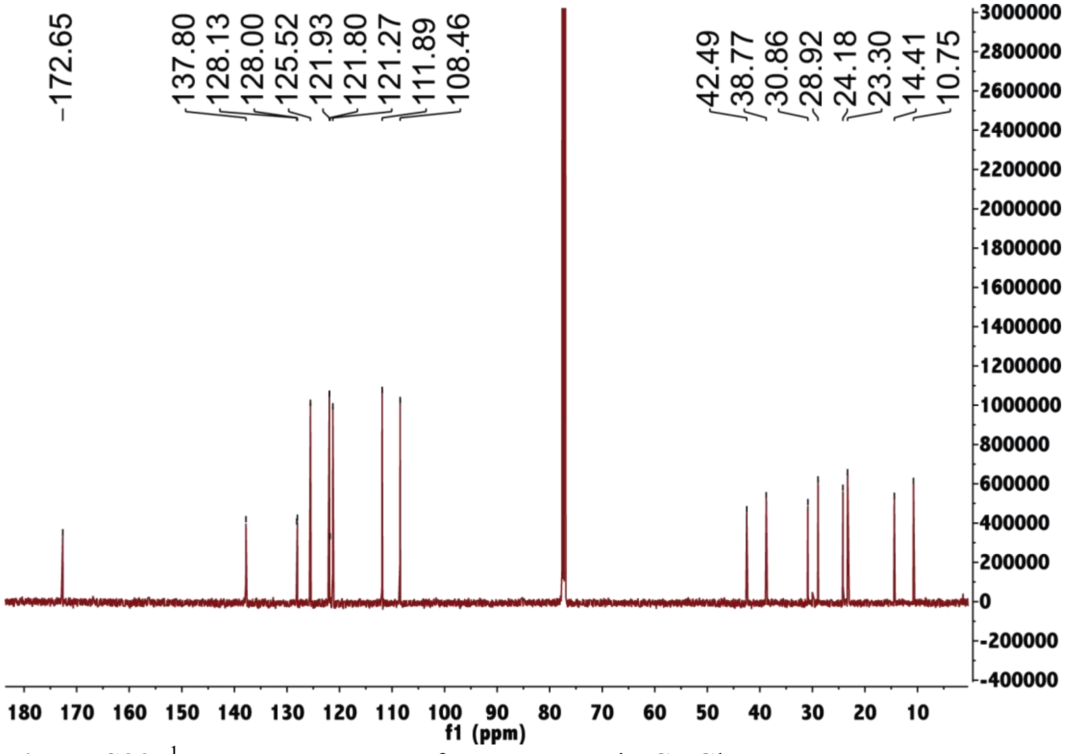

Figure S29. ^{1}H NMR spectrum of **MI-In-EHx** in $CDCl_3$.

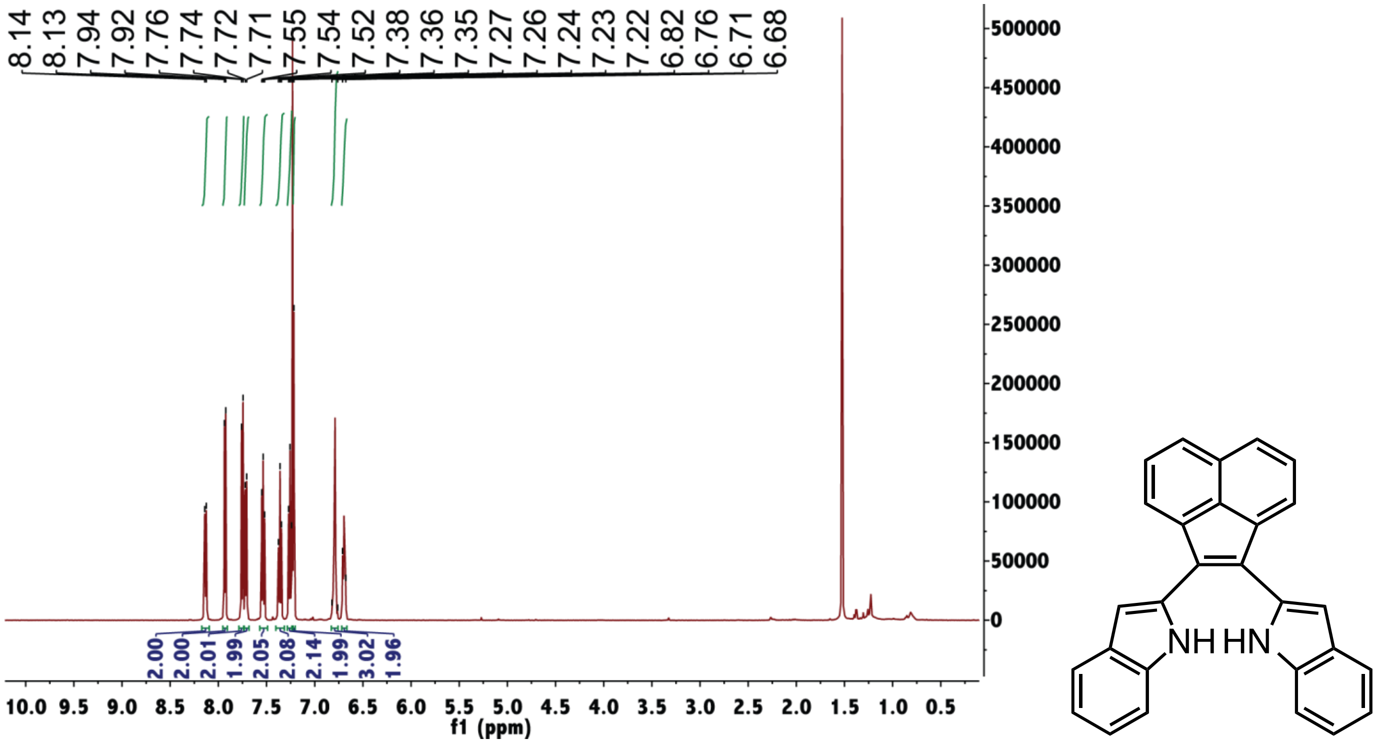

Figure S30. ^{1}H NMR spectrum of **AN-In** in $CDCl_3$.

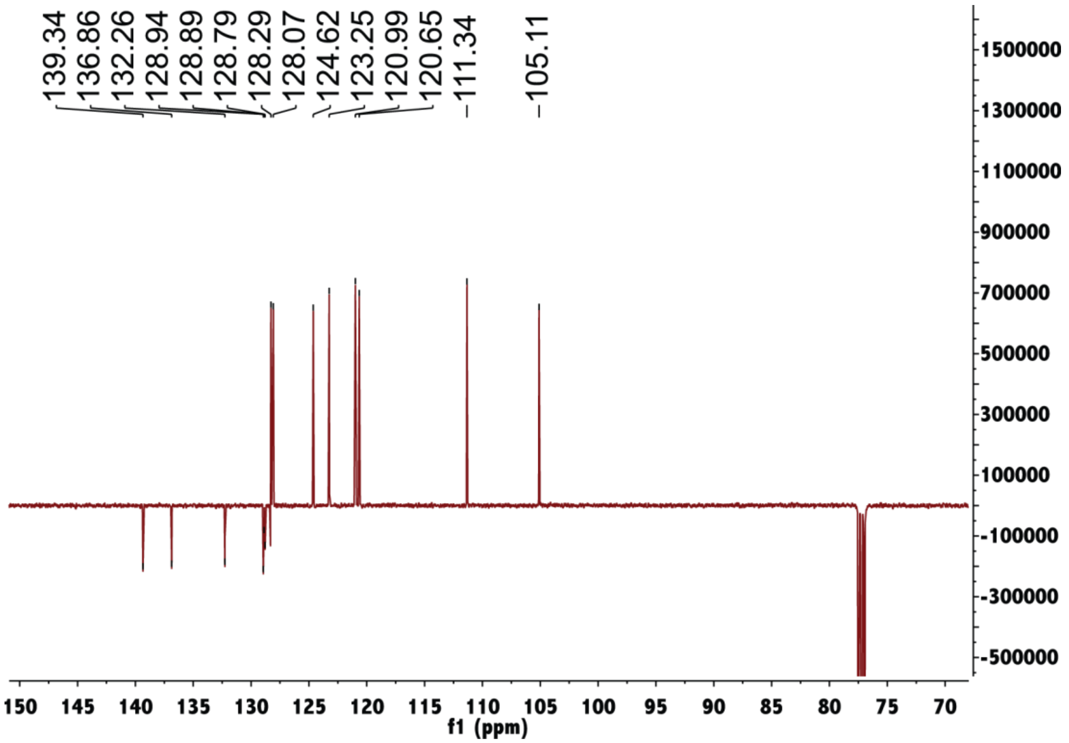

Figure S31. ^{13}C NMR spectrum of **AN-In** in $CDCl_3$.

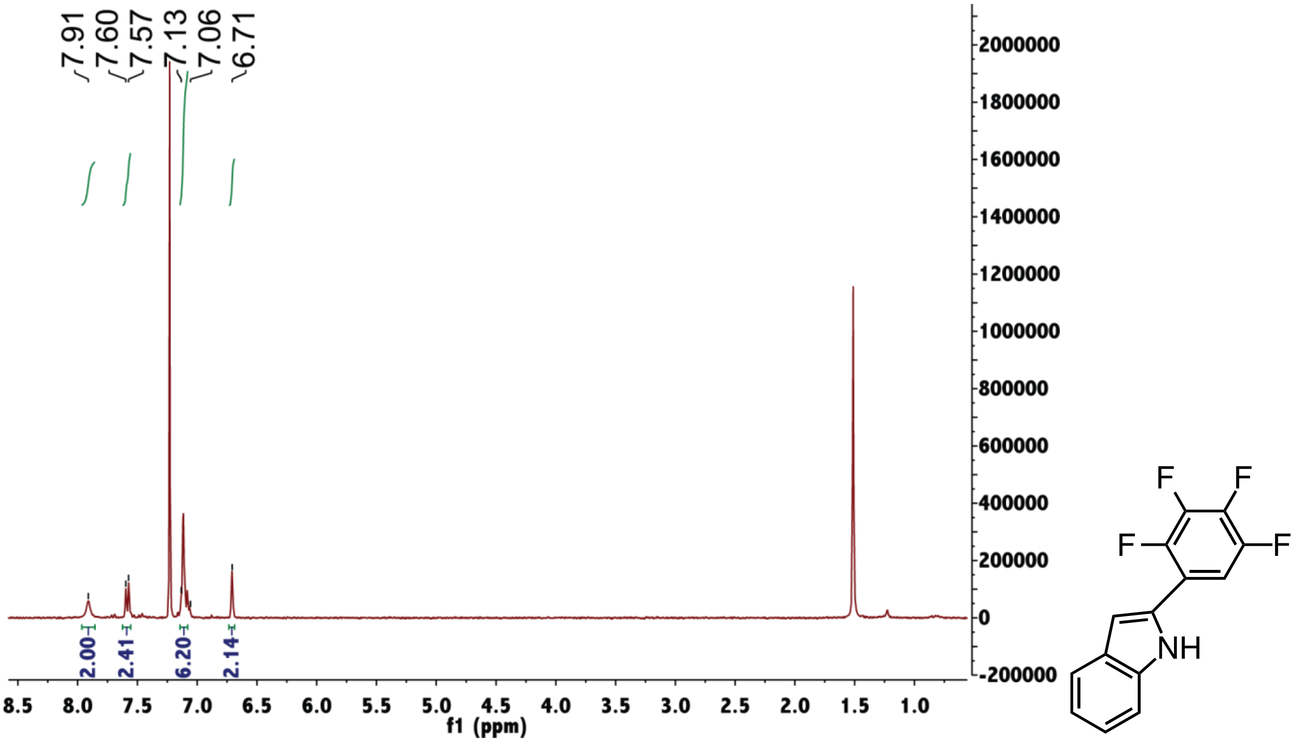

Figure S32. ^{1}H NMR spectrum of **mFBZ-In** in $CDCl_3$.

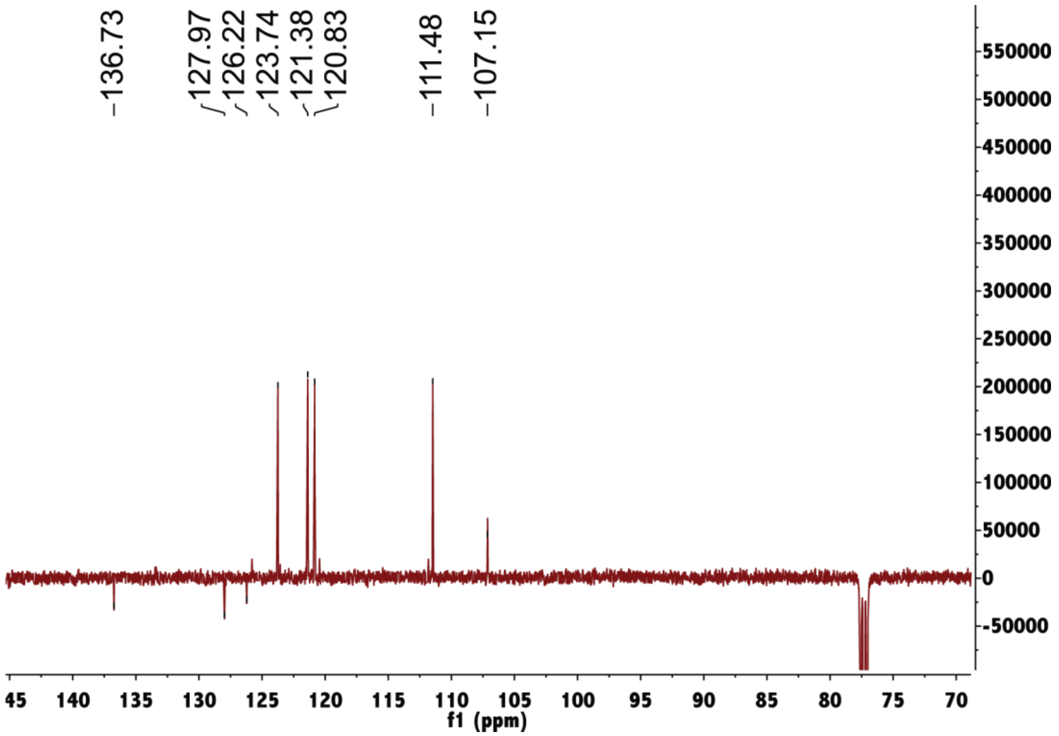

Figure S33. ^{13}C NMR spectrum of **mFBZ-In** in $CDCl_3$.

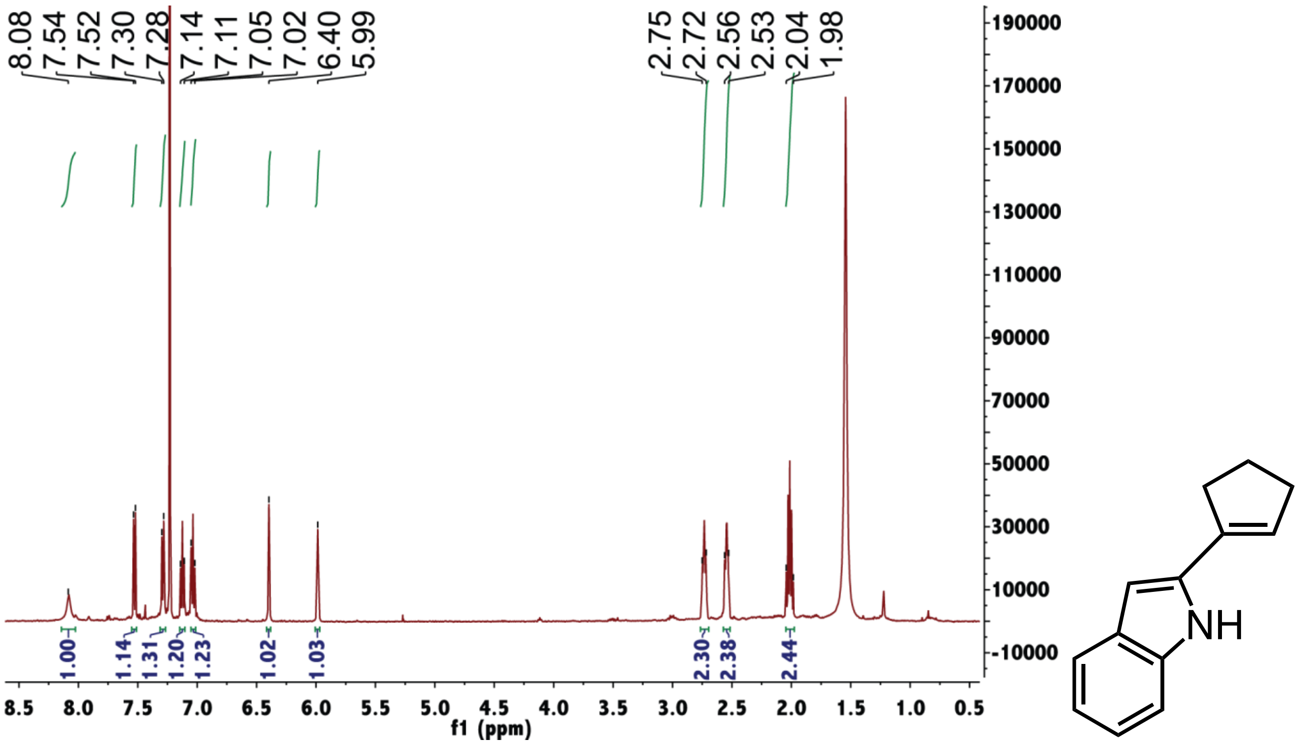

Figure S34. ^{1}H NMR spectrum of **mCP-In** in $CDCl_3$.

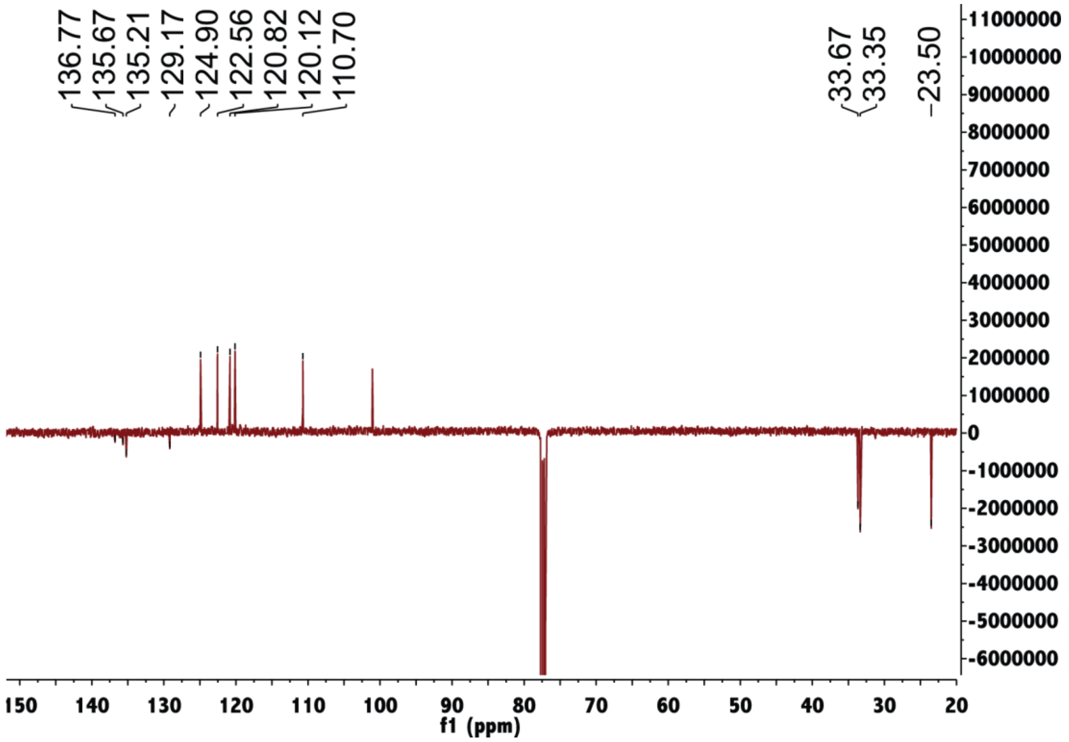

Figure S35. ^{13}C NMR spectrum of **mCP-In** in $CDCl_3$.

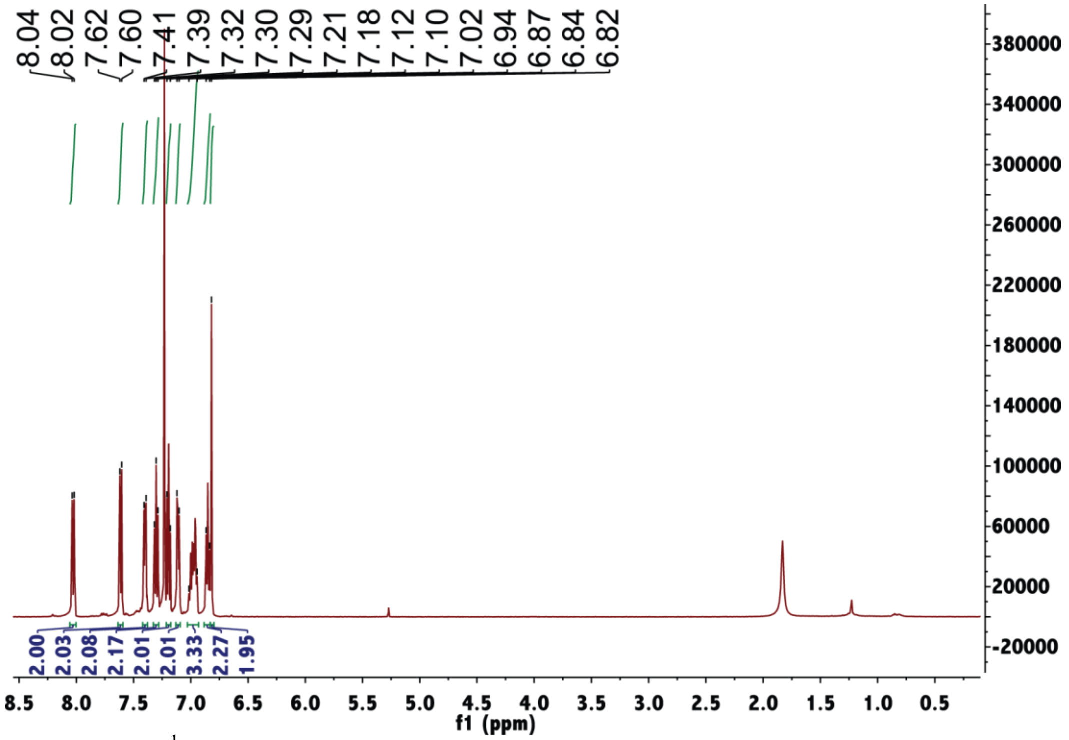

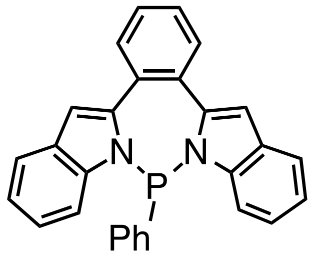

Figure S36. ^{1}H NMR spectrum of **BZ-P** in $CDCl_3$.

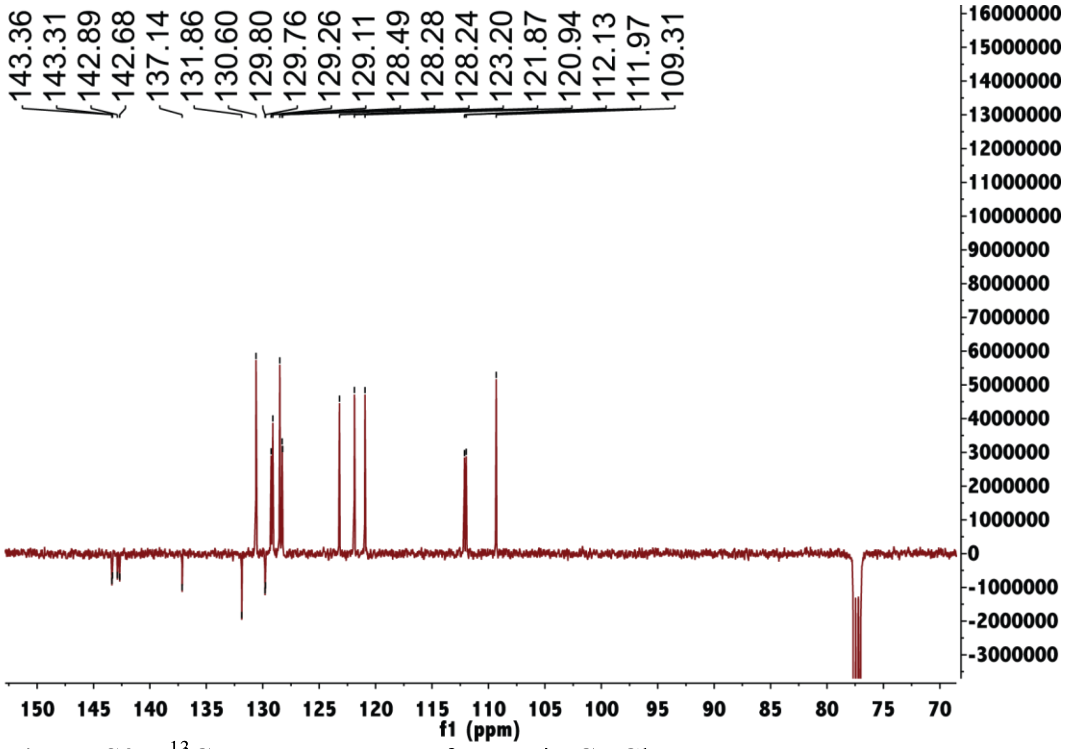

Figure S37. ^{13}C NMR spectrum of **BZ-P** in $CDCl_3$.

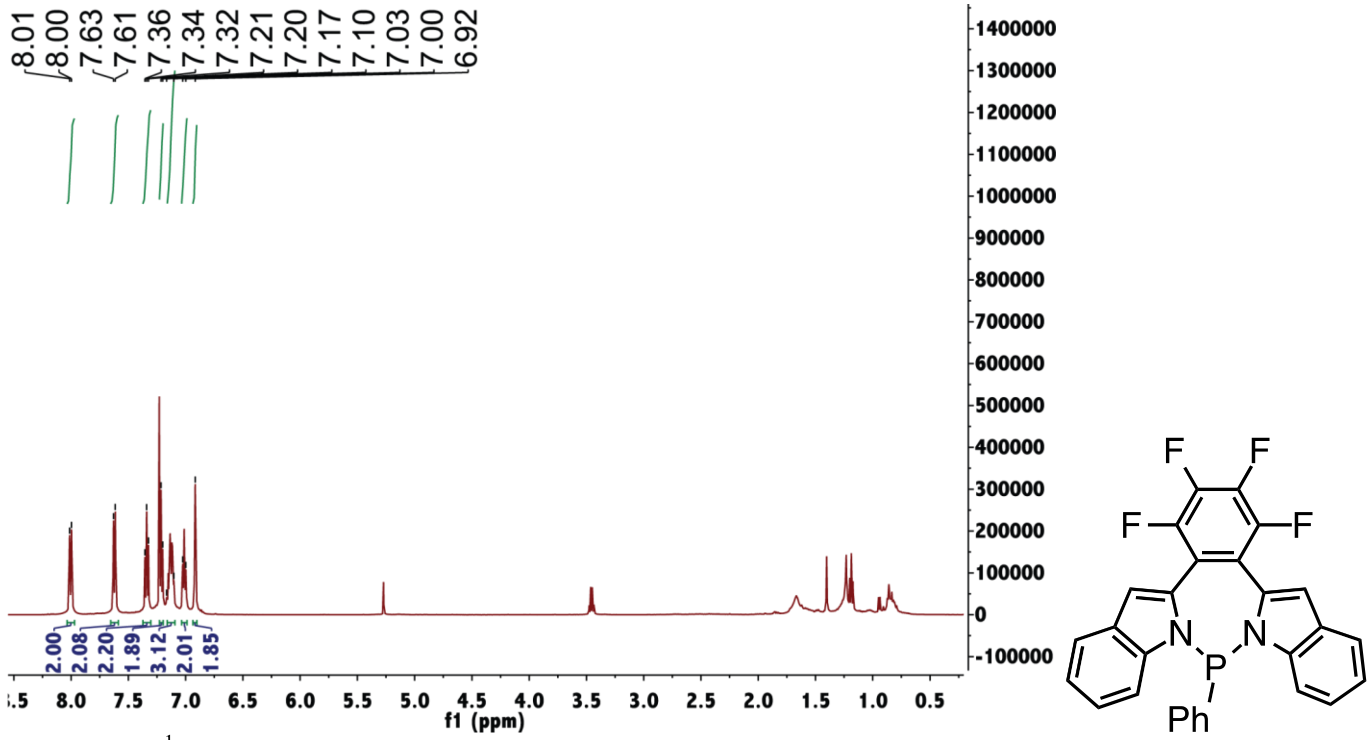

Figure S38. ^{1}H NMR spectrum of **FBZ-P** in $CDCl_3$.

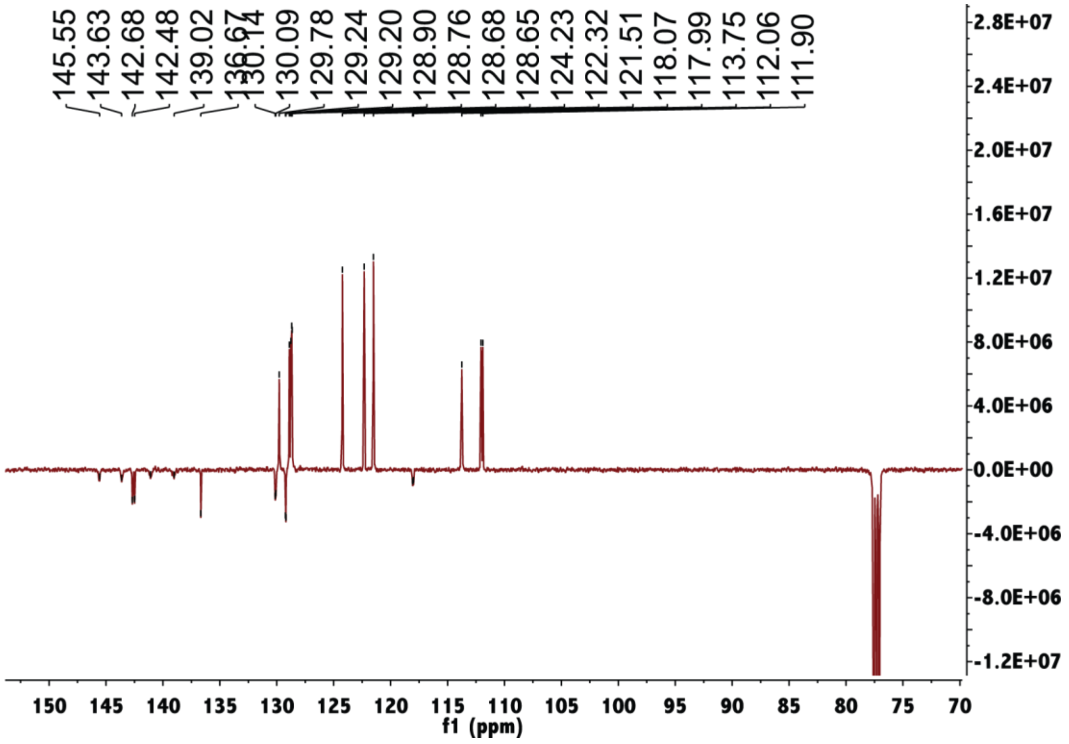

Figure S39. ^{13}C NMR spectrum of **FBZ-P** in $CDCl_3$.

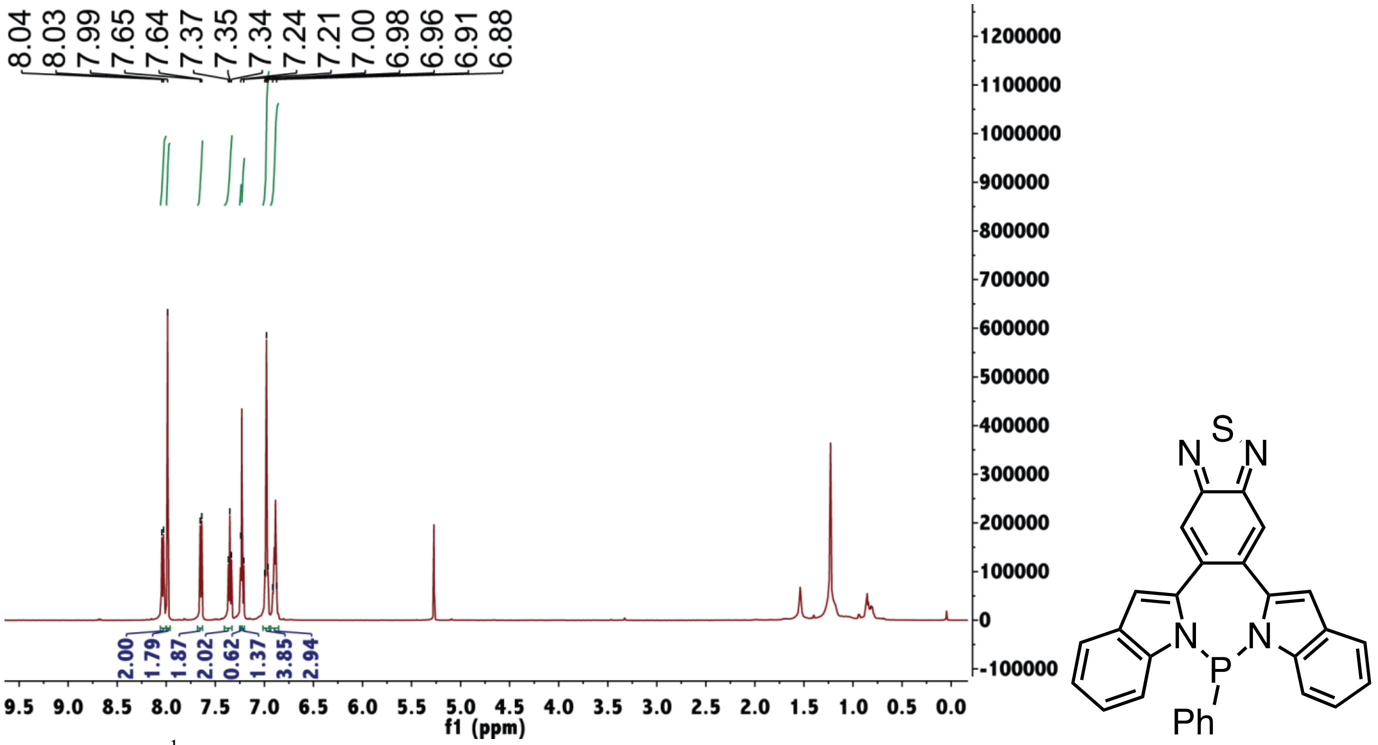

Figure S40. ^{1}H NMR spectrum of **BTD-P** in $CDCl_3$.

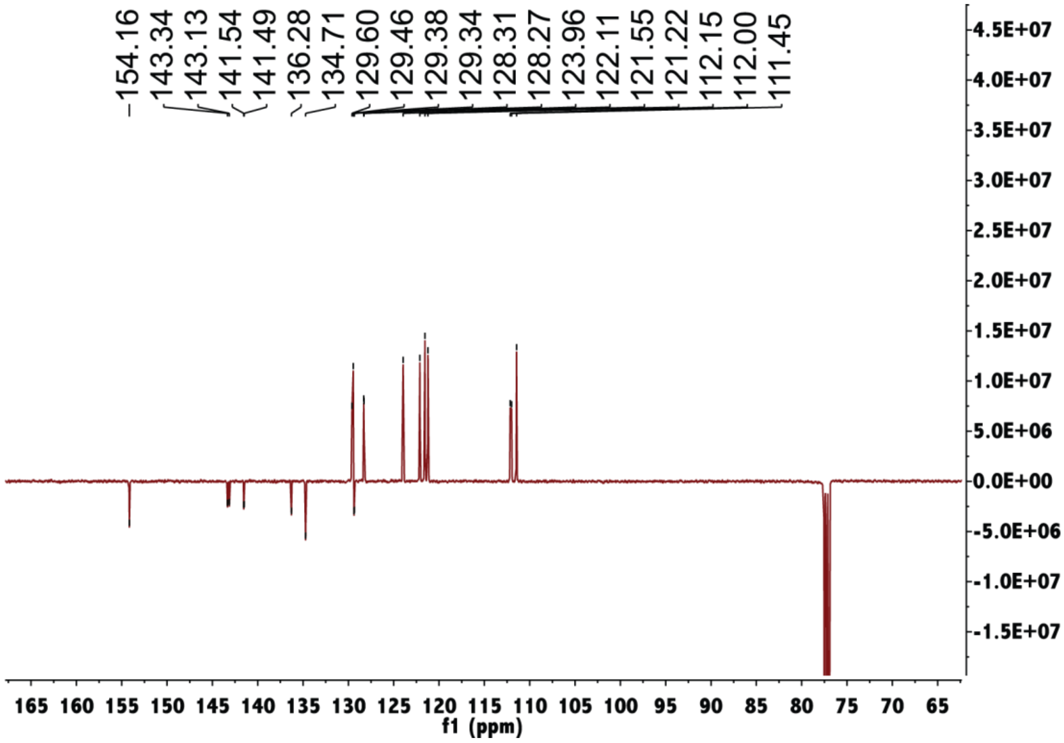

Figure S41. ^{1}H NMR spectrum of **BTD-P** in $CDCl_3$.

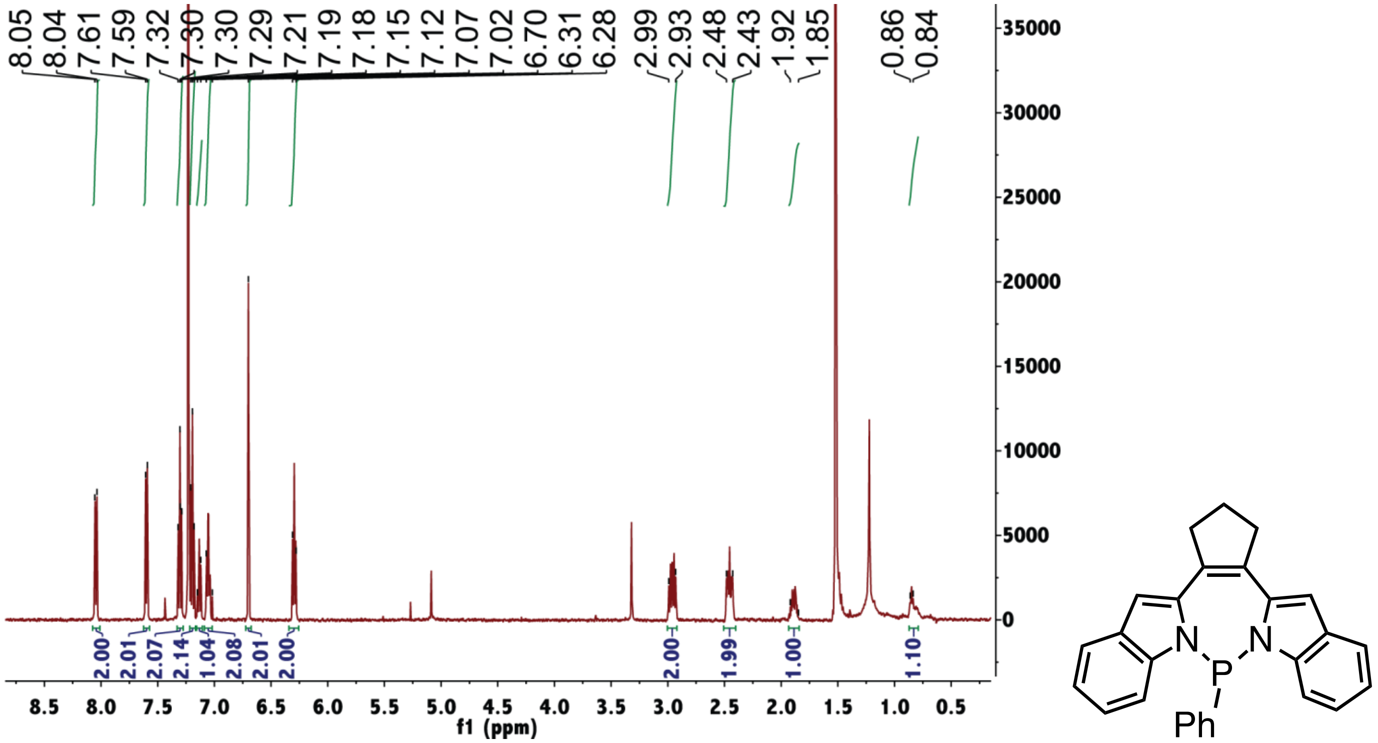

Figure S42. ^{1}H NMR spectrum of **CP-P** in $CDCl_3$.

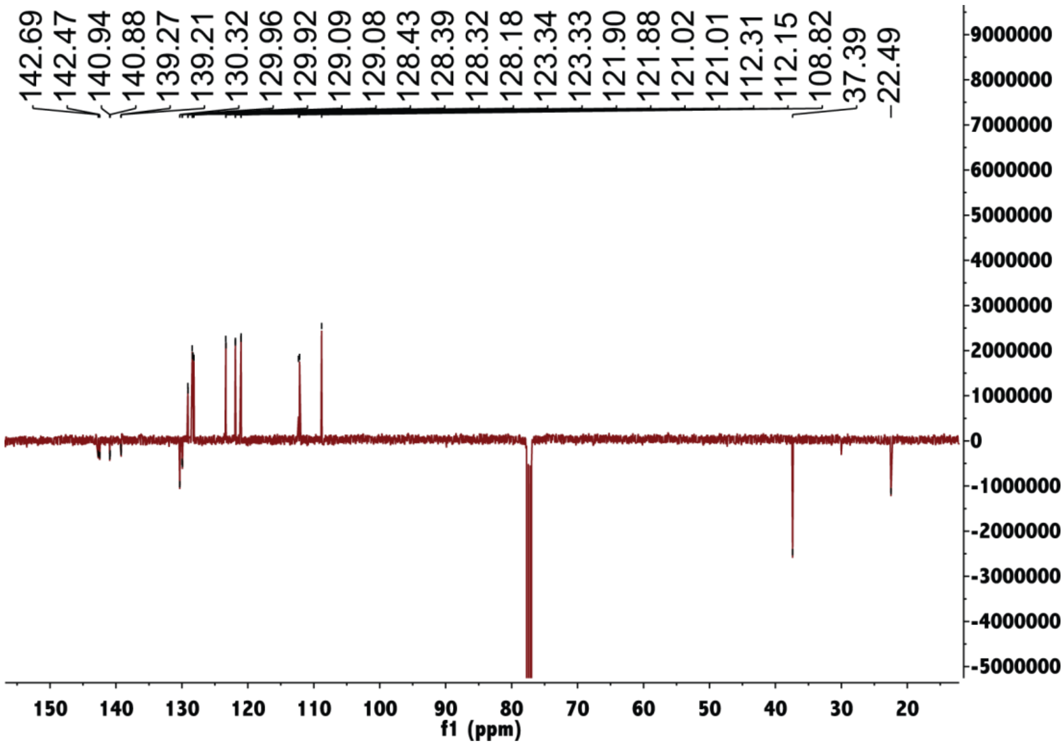

Figure S43. ^{13}C NMR spectrum of **CP-P** in $CDCl_3$.

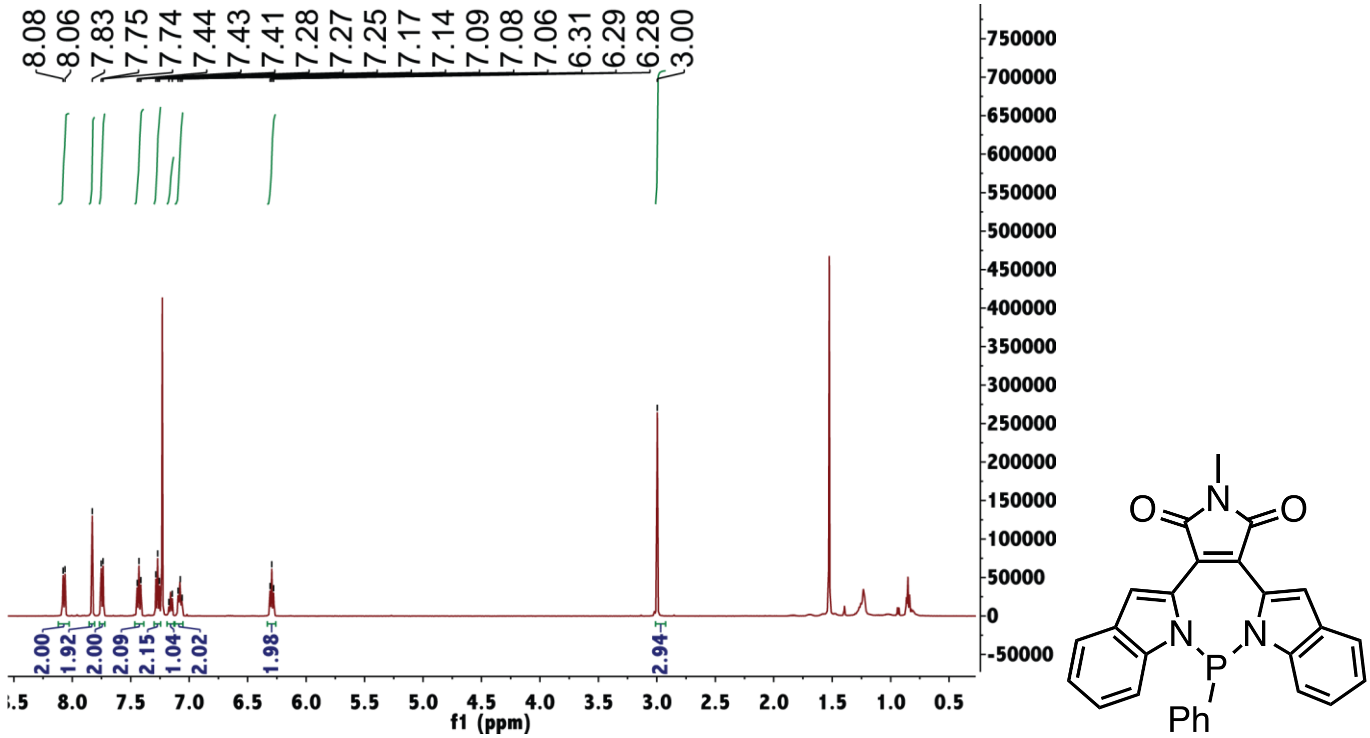

Figure S44. ^{1}H NMR spectrum of **MI-P** in $CDCl_3$.

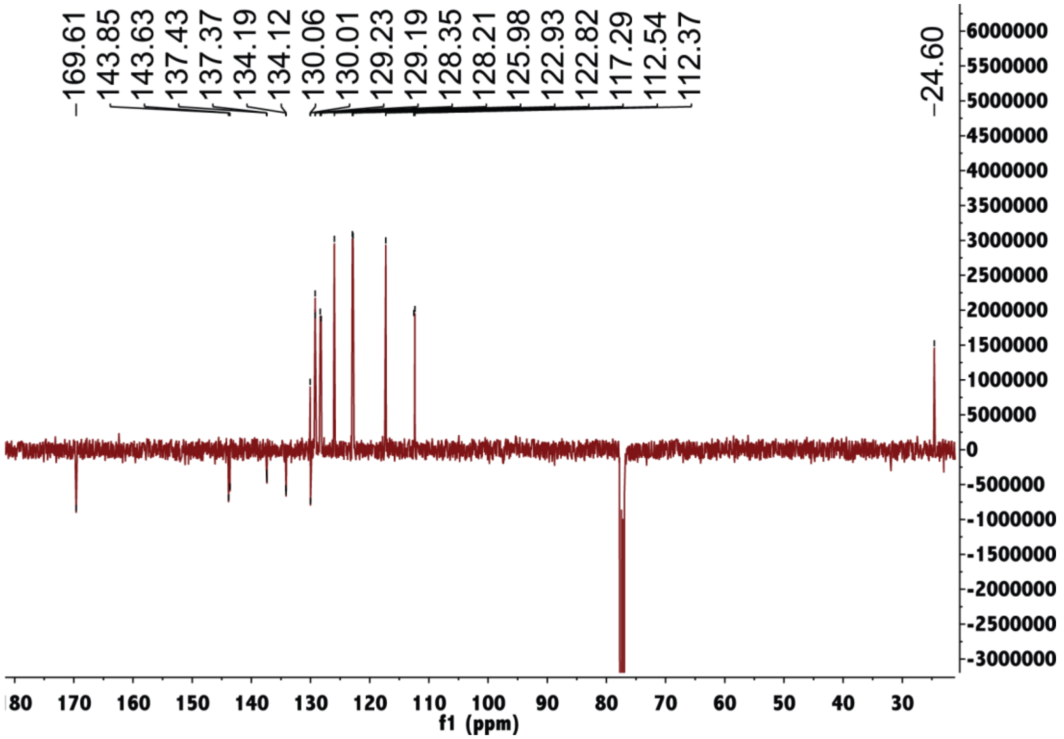

Figure S45. ^{13}C NMR spectrum of **MI-P** in $CDCl_3$.

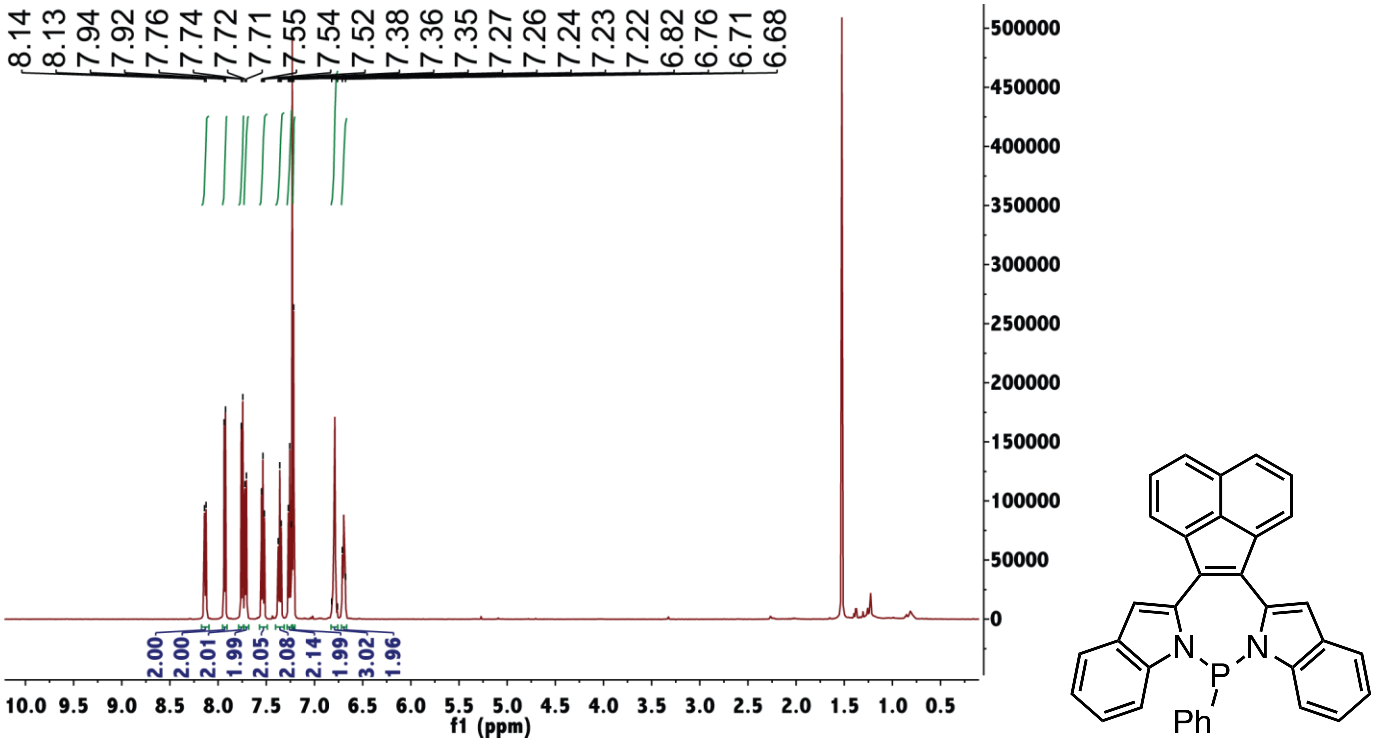

Figure S46. ^{1}H NMR spectrum of **AN-P** in $CDCl_3$.

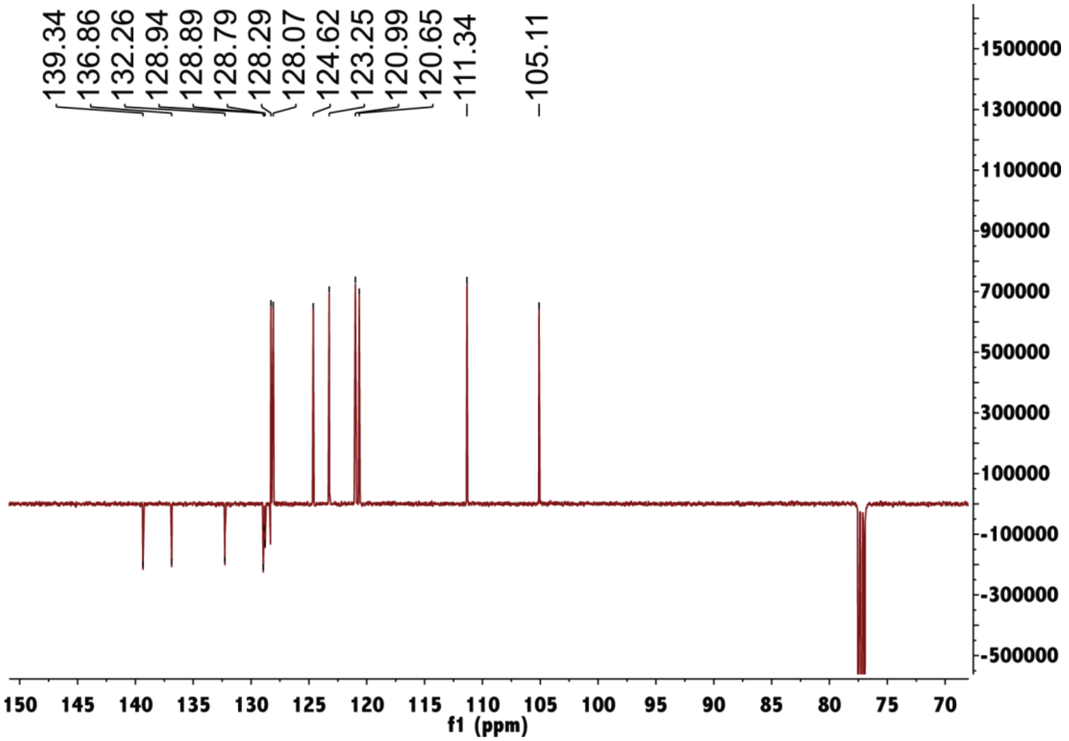

Figure S47. ^{13}C NMR spectrum of **AN-P** in $CDCl_3$.

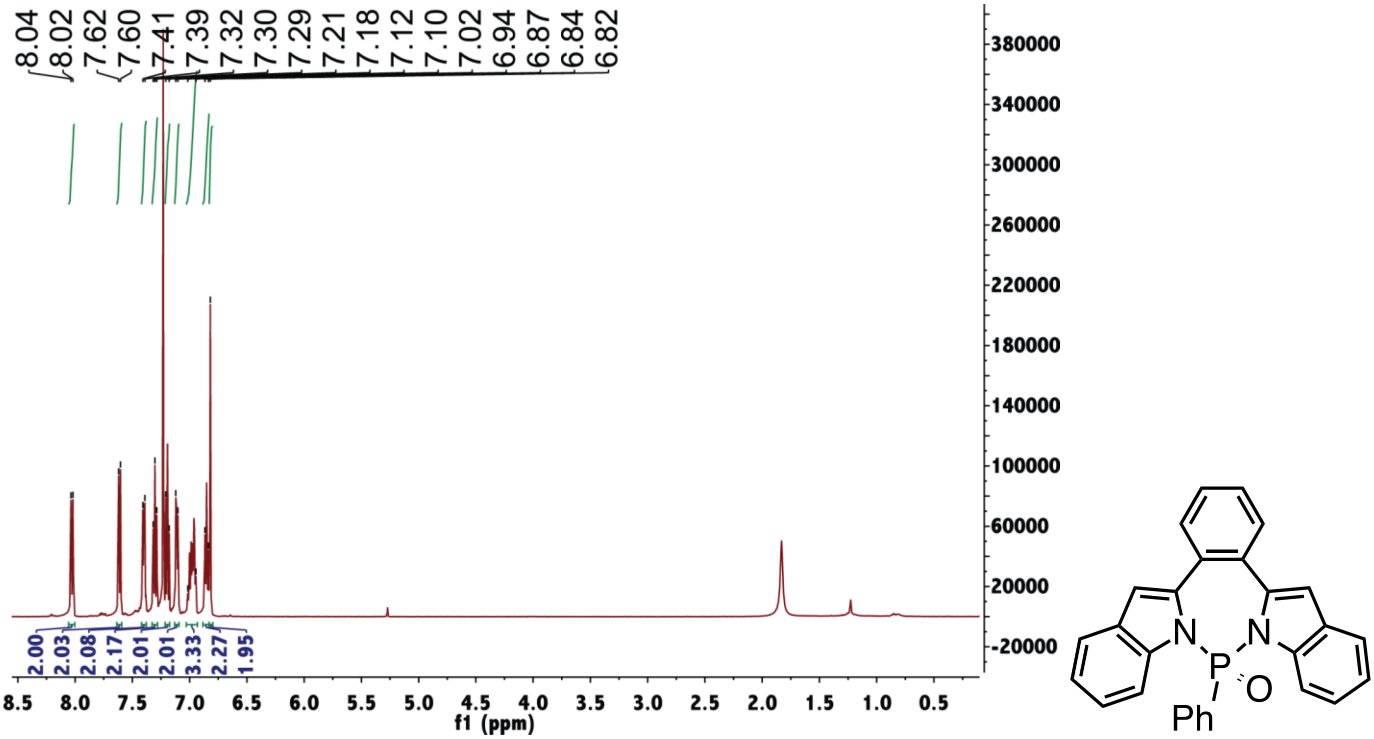

Figure S48. ^{1}H NMR spectrum of **BZ-PO** in $CDCl_3$.

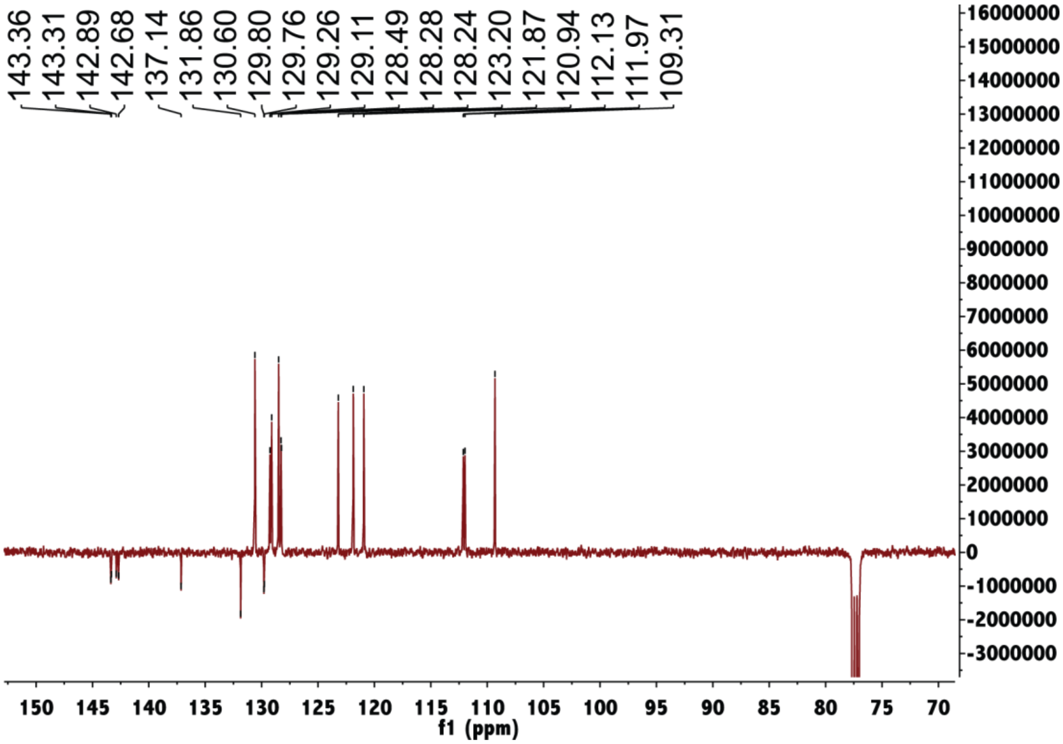

Figure S49. ^{13}C NMR spectrum of **BZ-PO** in $CDCl_3$.

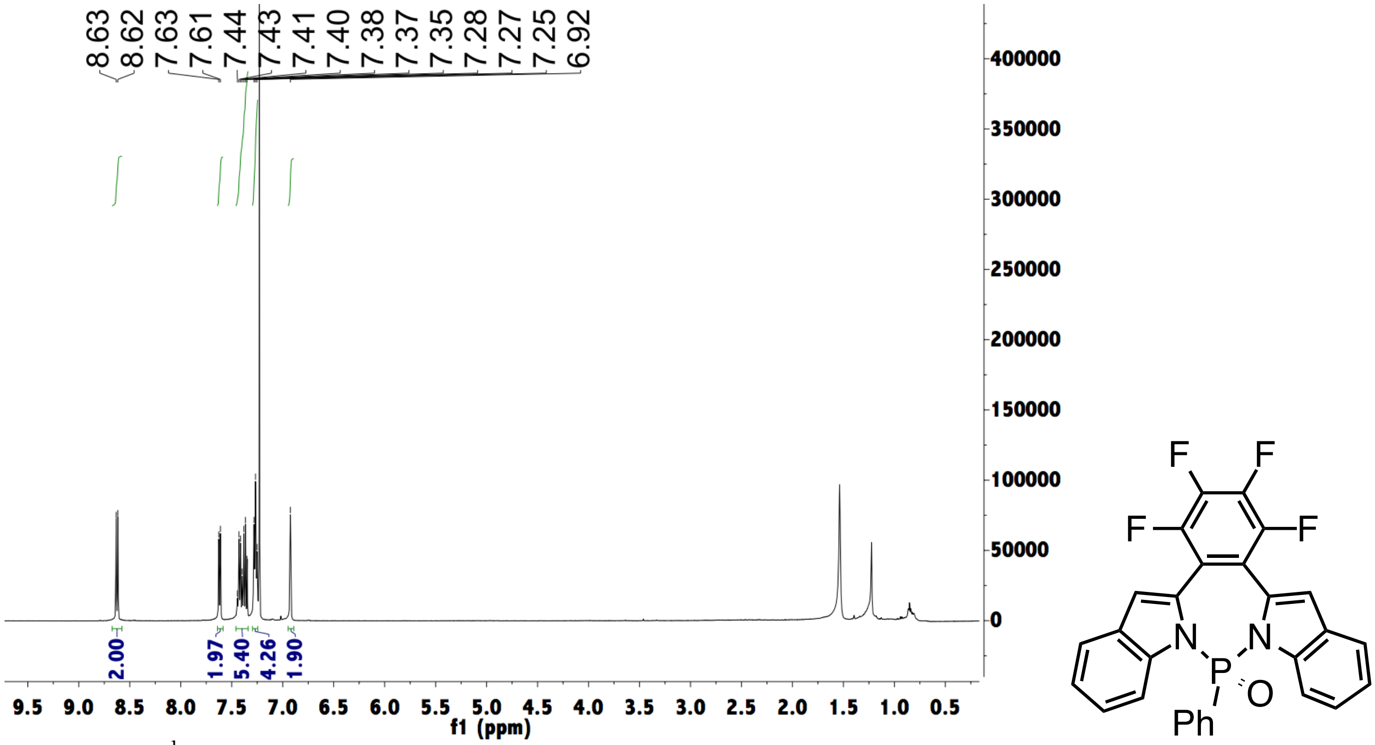

Figure S50. ^{1}H NMR spectrum of **FBZ-PO** in $CDCl_3$.

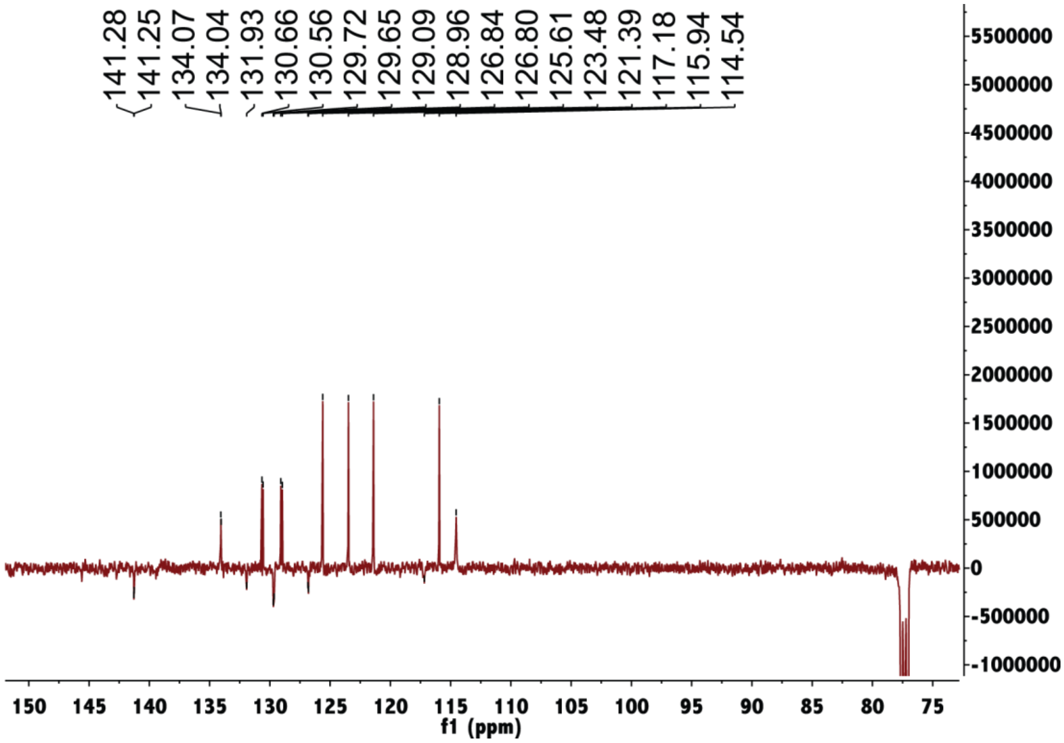

Figure S51. ^{13}C NMR spectrum of **FBZ-PO** in $CDCl_3$.

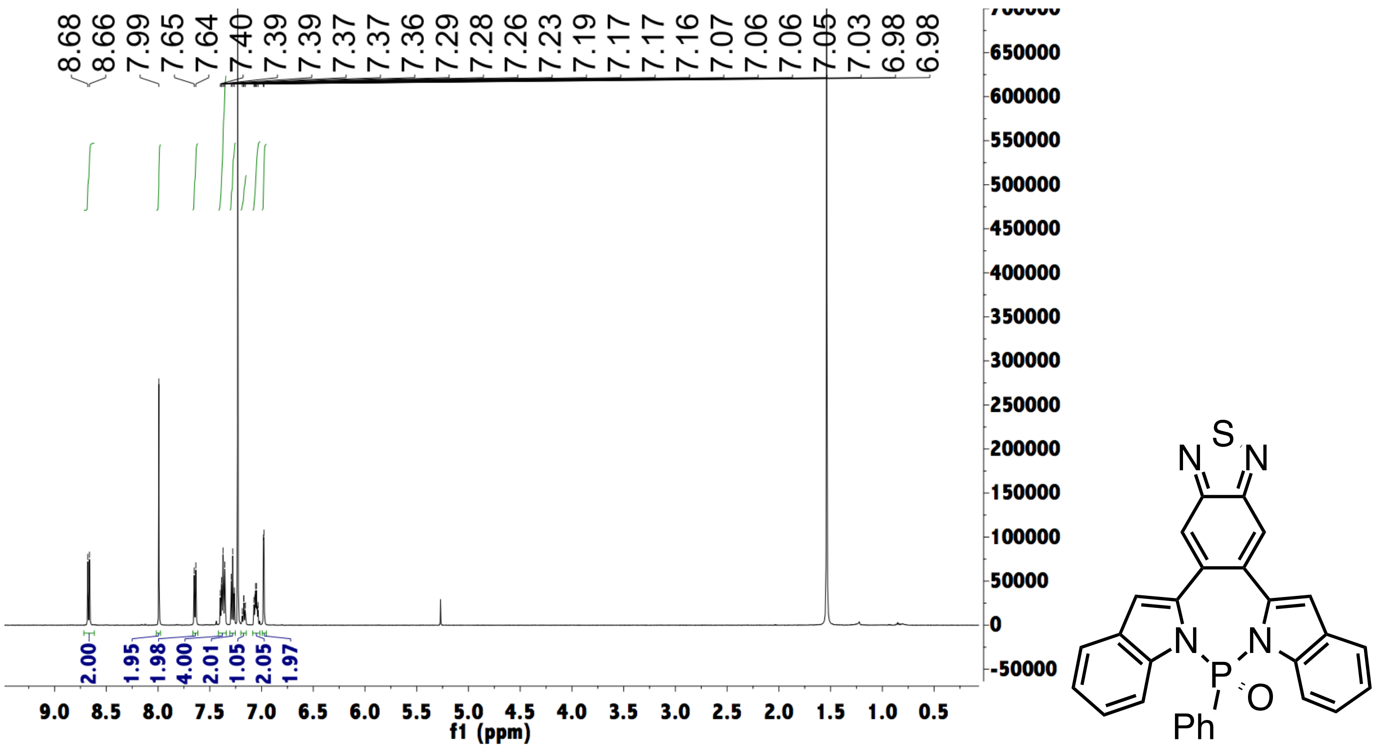

Figure S52. ^{1}H NMR spectrum of **BTD-PO** in $CDCl_3$.

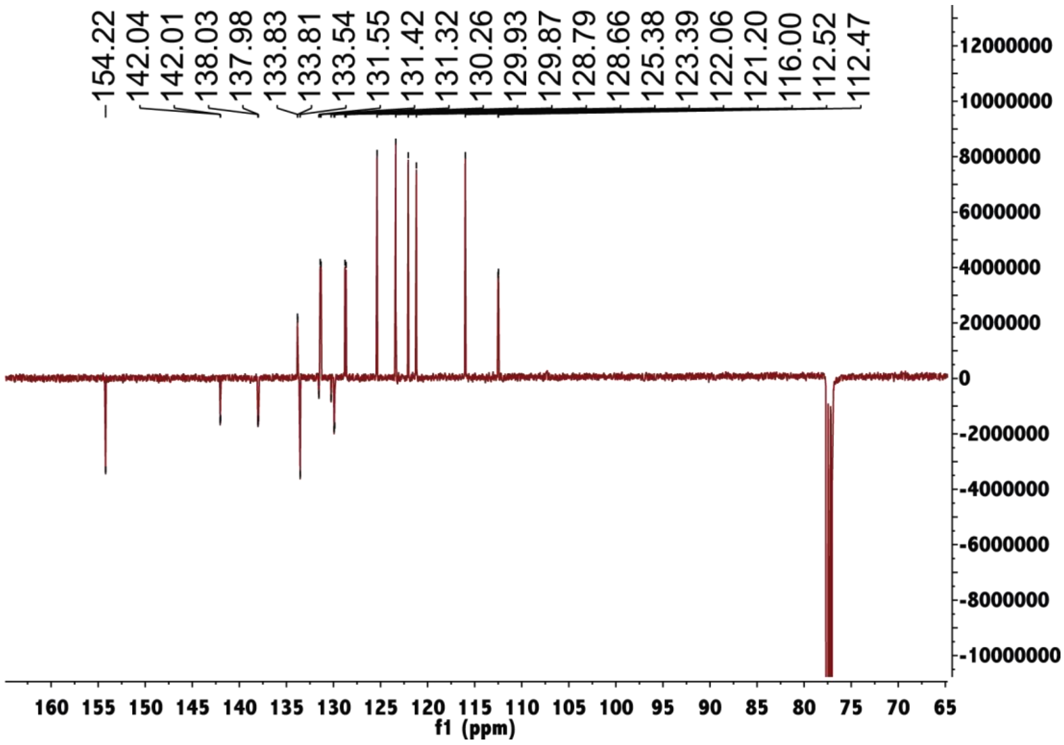

Figure S53. ^{13}C NMR spectrum of **BTD-PO** in $CDCl_3$.

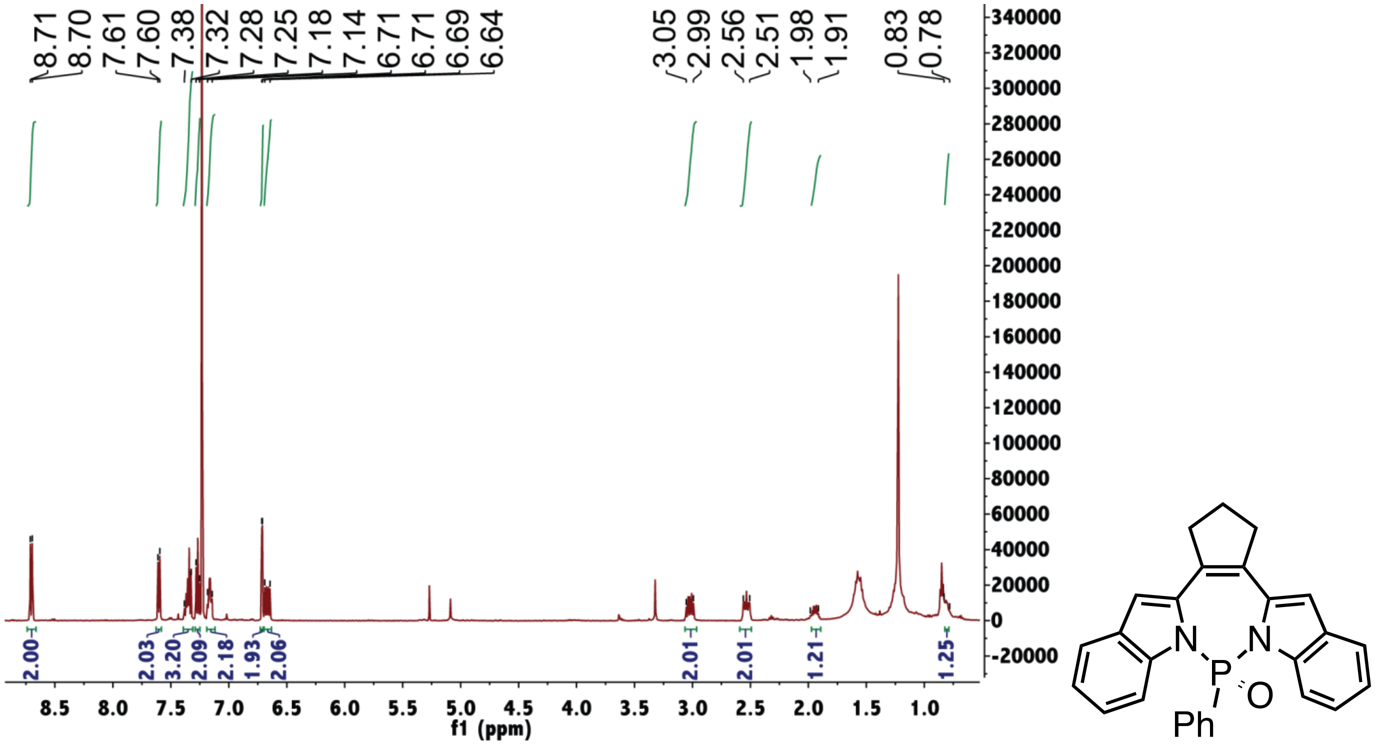

Figure S54. ^{1}H NMR spectrum of **CP-PO** in $CDCl_3$.

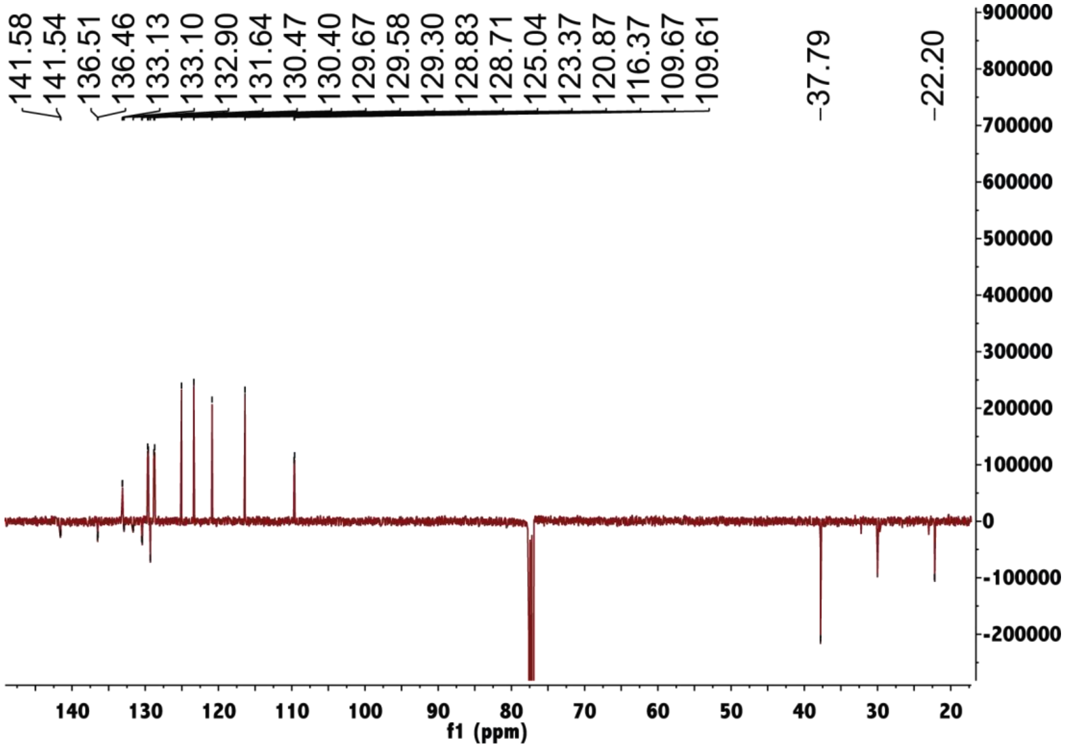

Figure S55. ^{13}C NMR spectrum of **CP-PO** in $CDCl_3$.

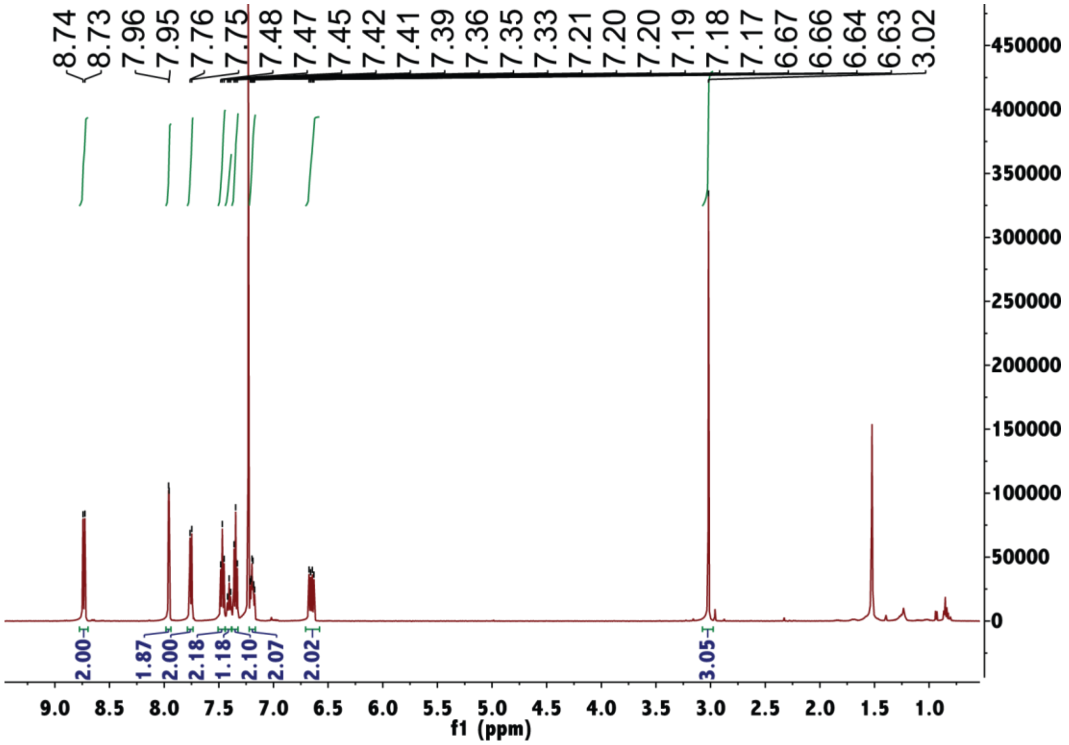

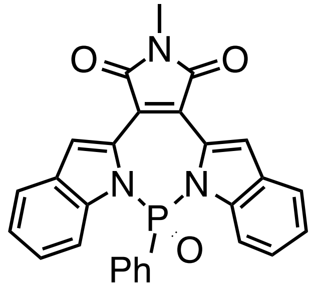

Figure S56. ^{1}H NMR spectrum of **MI-PO** in $CDCl_3$.

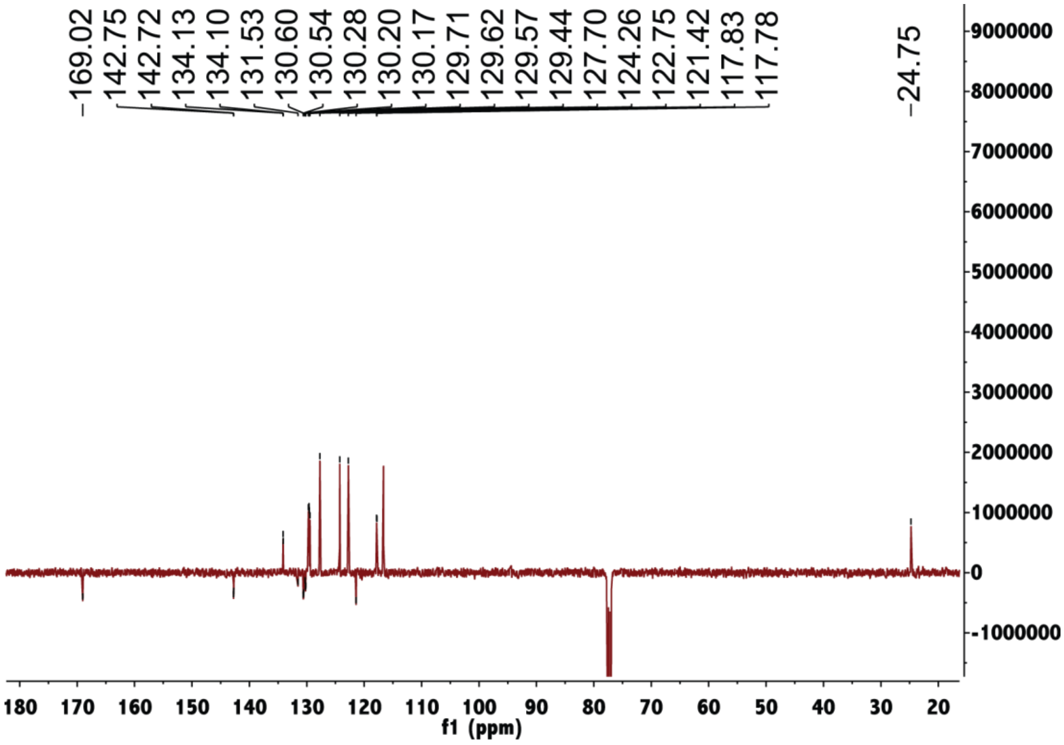

Figure S57. ^{13}C NMR spectrum of **MI-PO** in $CDCl_3$.

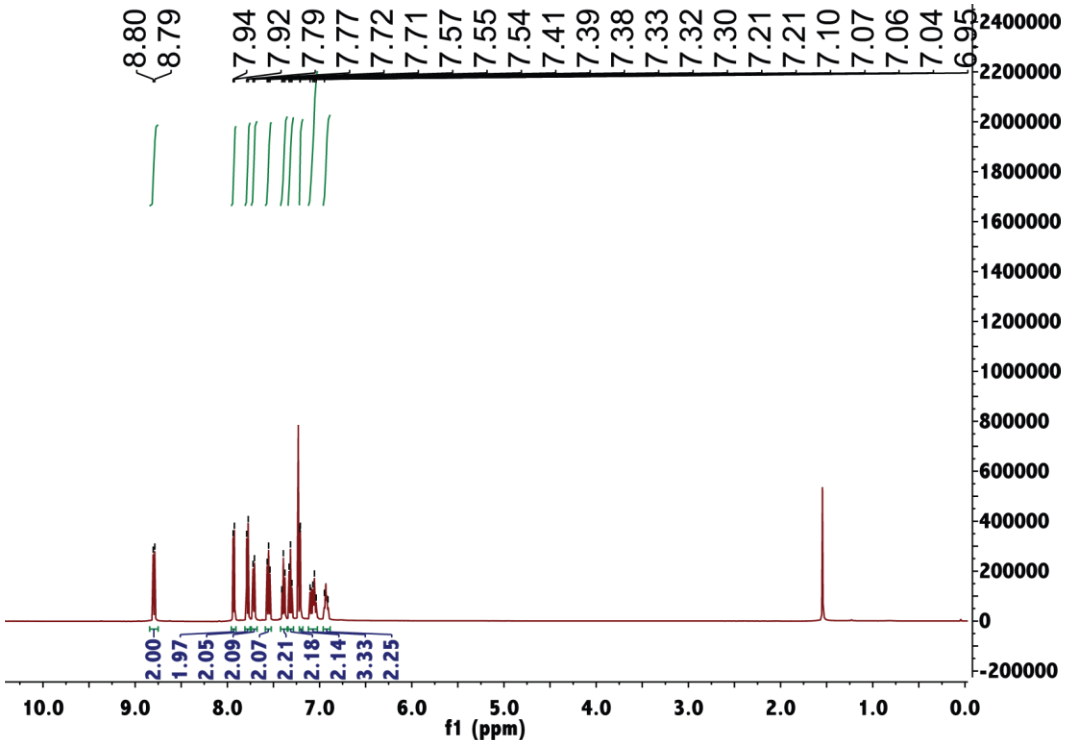

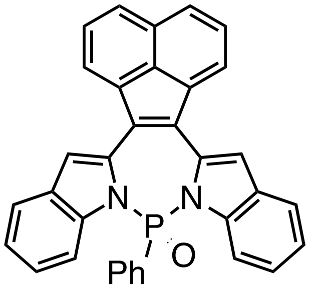

Figure S58. ^{1}H NMR spectrum of **AN-PO** in $CDCl_3$.

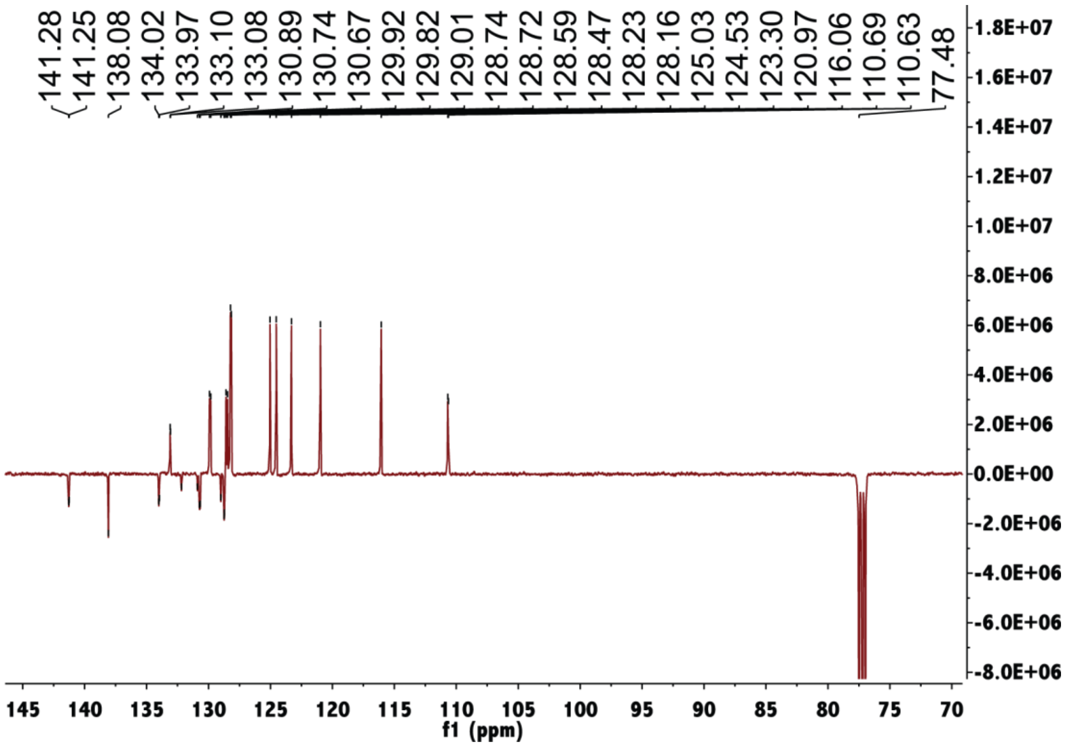

Figure S59. ^{13}C NMR spectrum of **AN-PO** in $CDCl_3$.

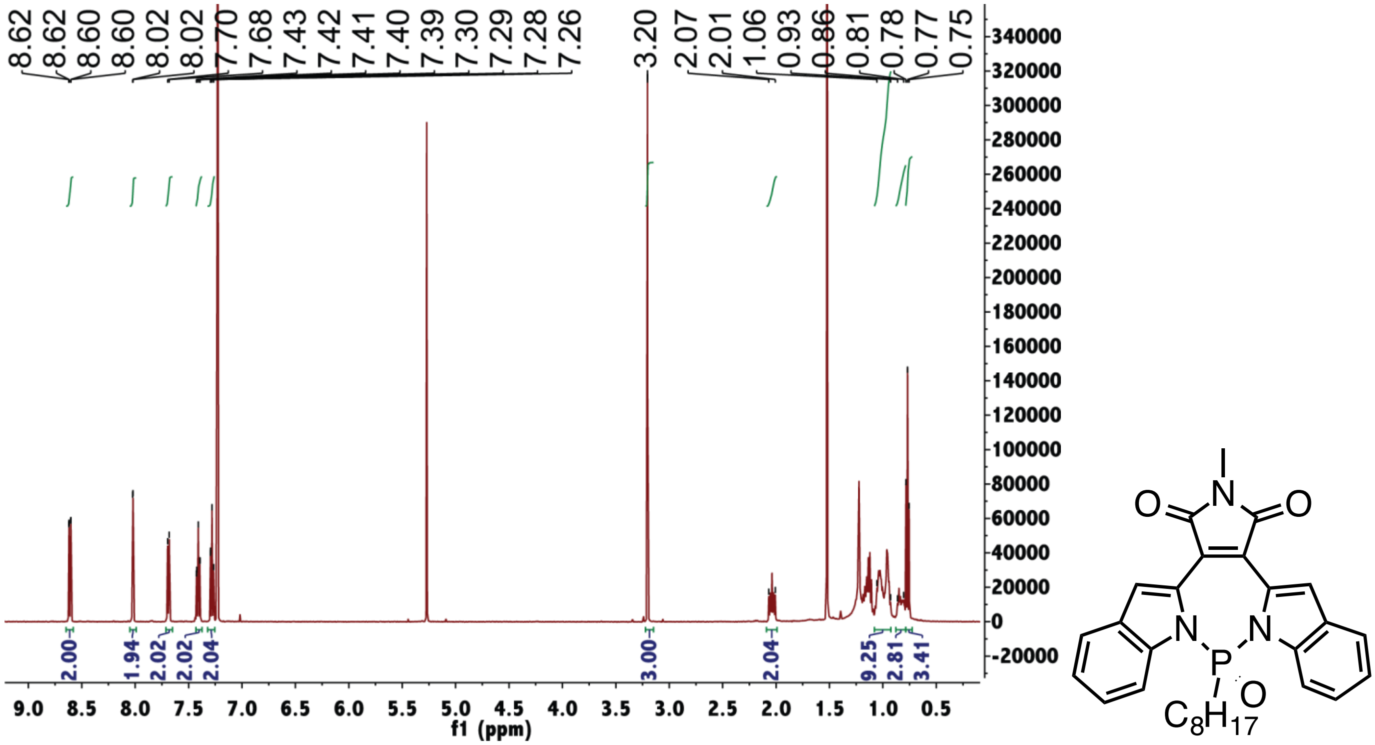

Figure S60. ^{1}H NMR spectrum of **MI-PO-C8** in $CDCl_3$.

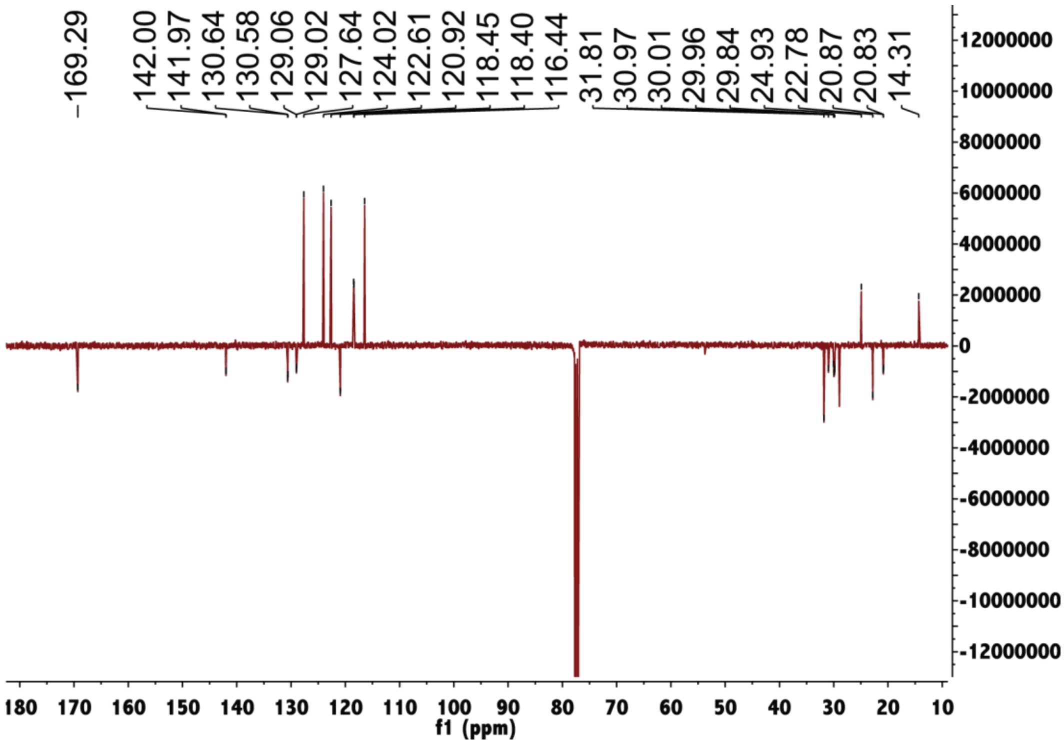

Figure S61. ^{13}C NMR spectrum of **MI-PO-C8** in $CDCl_3$.

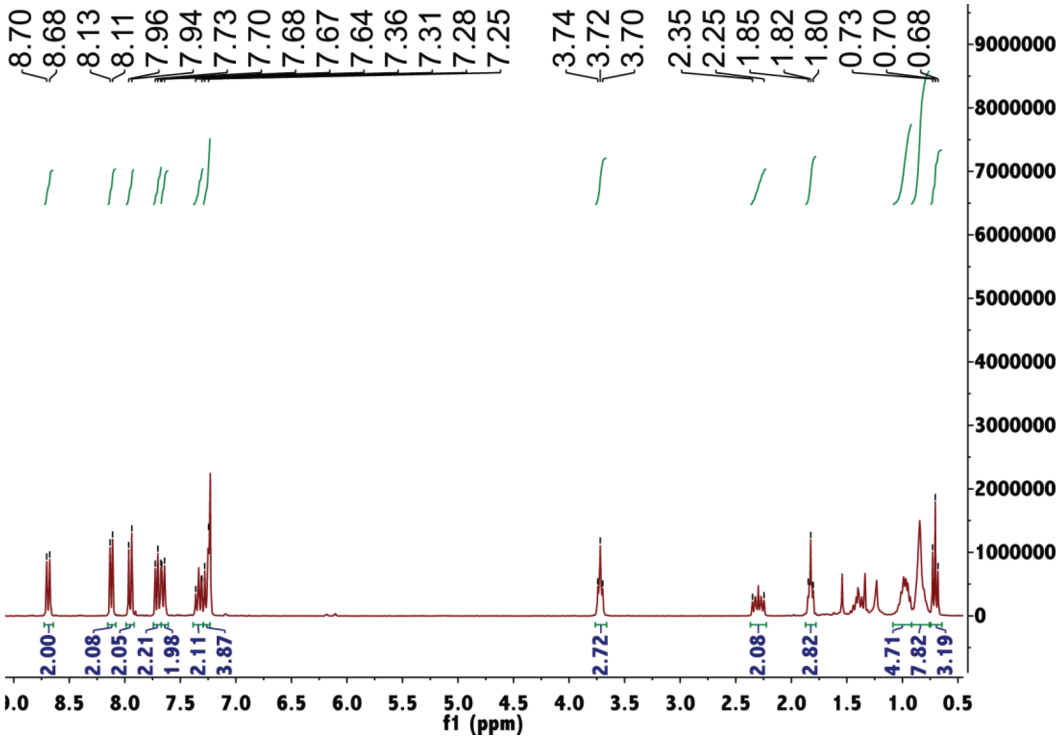

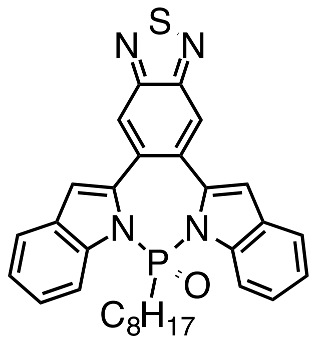

Figure S62. ^{1}H NMR spectrum of **BTD-PO-C8** in $CDCl_3$.

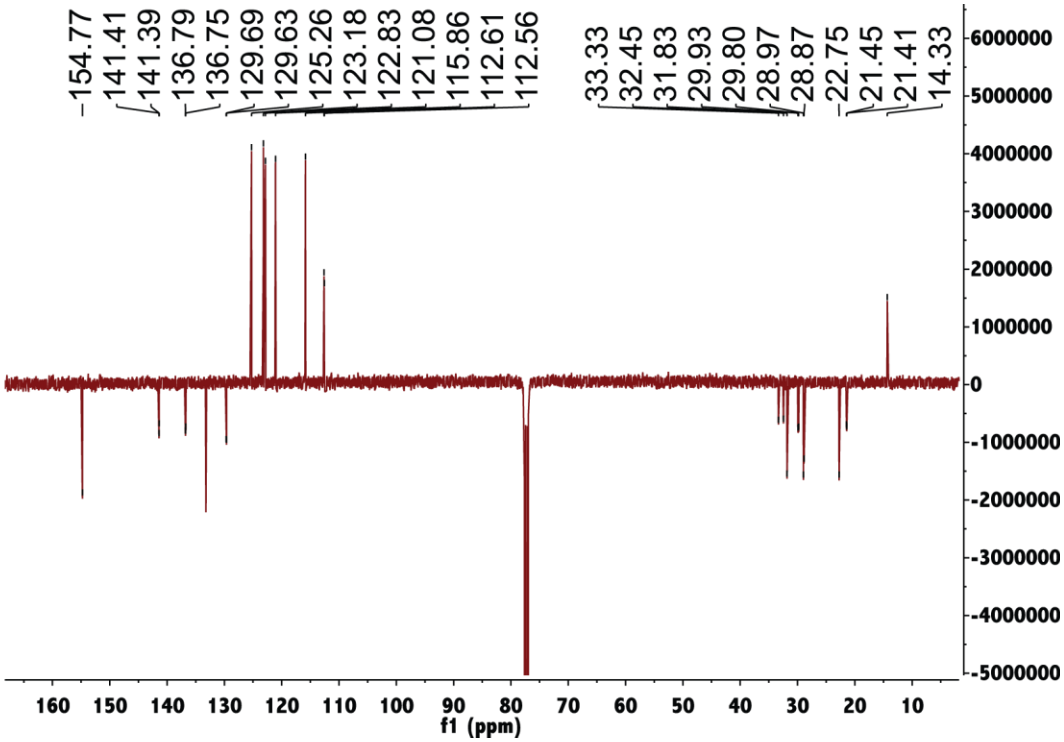

Figure S63. ^{13}C NMR spectrum of **BTD-PO-C8** in $CDCl_3$.

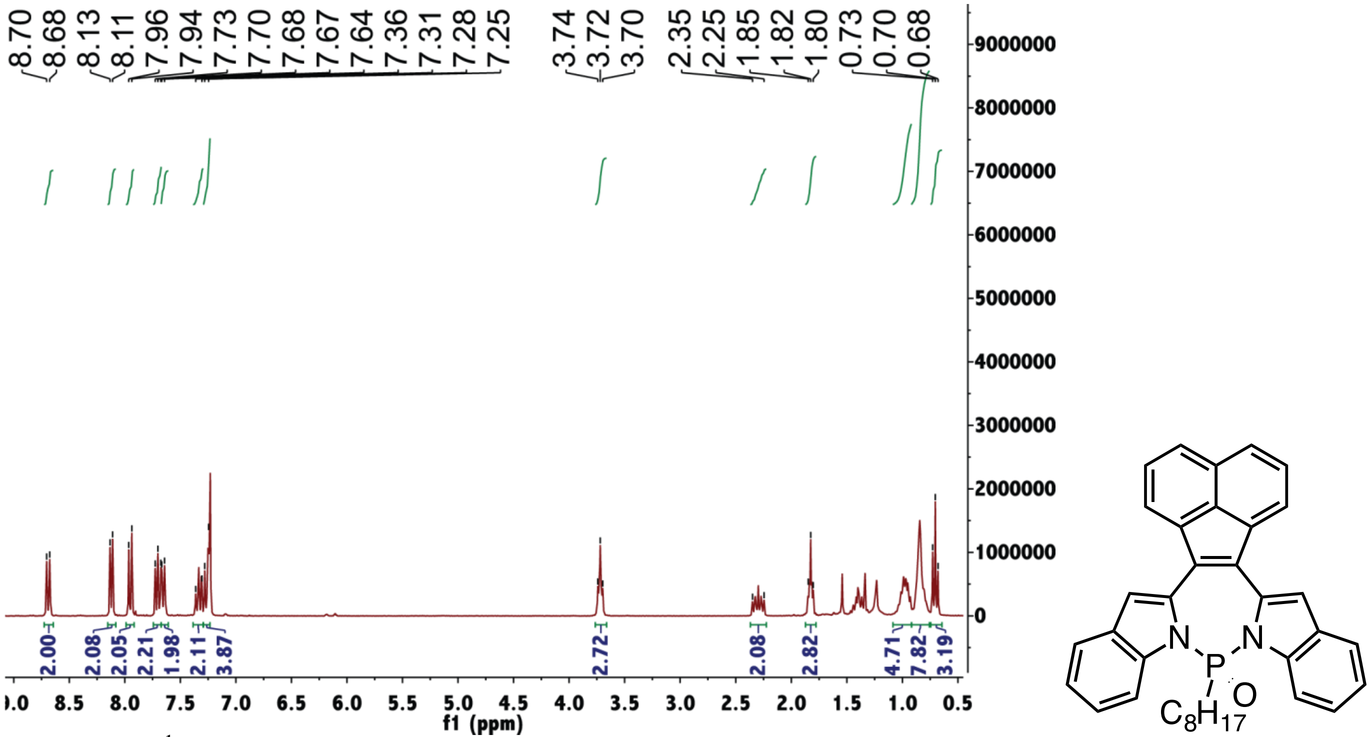

Figure S64. ^{1}H NMR spectrum of **AN-PO-C8** in $CDCl_3$.

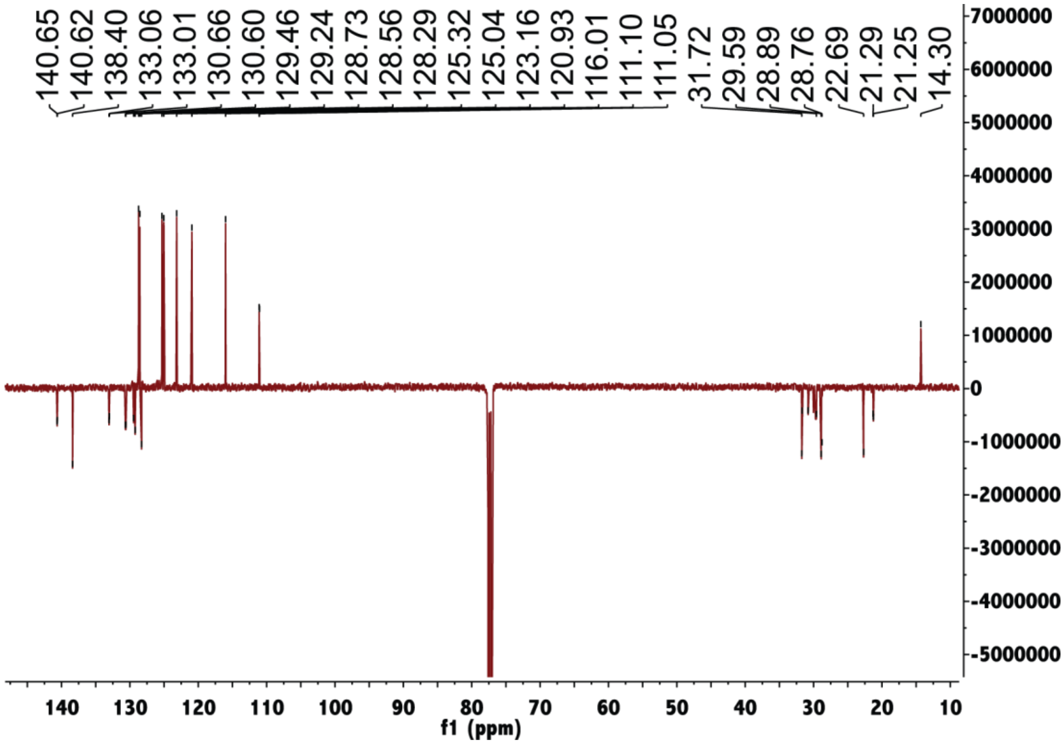

Figure S65. ^{13}C NMR spectrum of **AN-PO-C8** in $CDCl_3$.

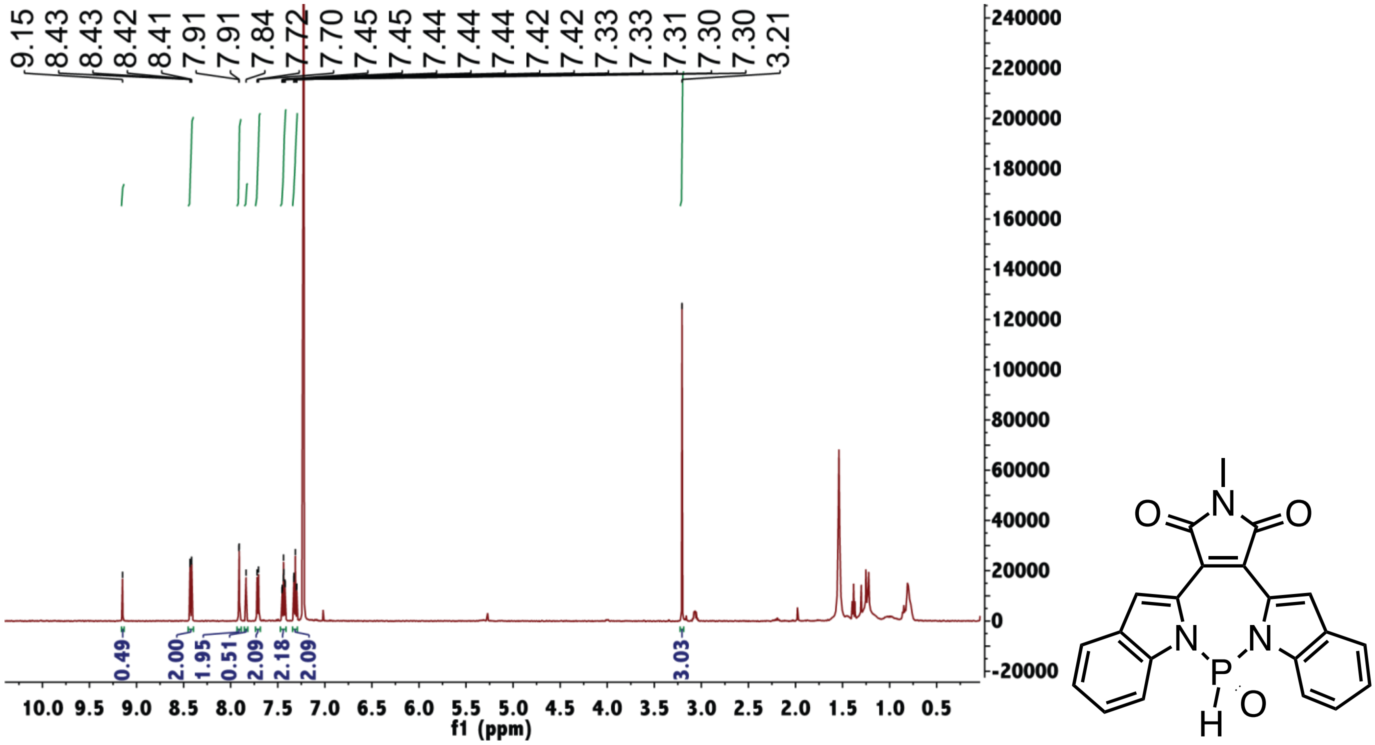

Figure S66. ^{1}H NMR spectrum of **MI-PO-H** in $CDCl_3$.

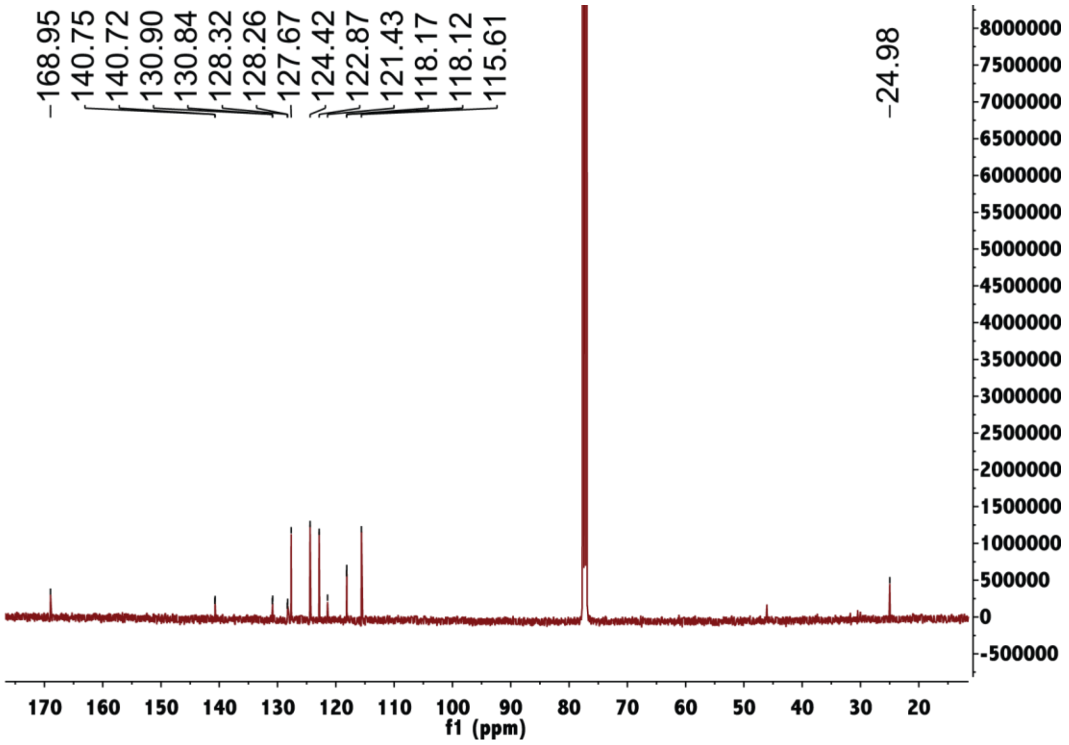

Figure S67. ^{13}C-NMR spectrum of **MI-PO-H** in $CDCl_3$.

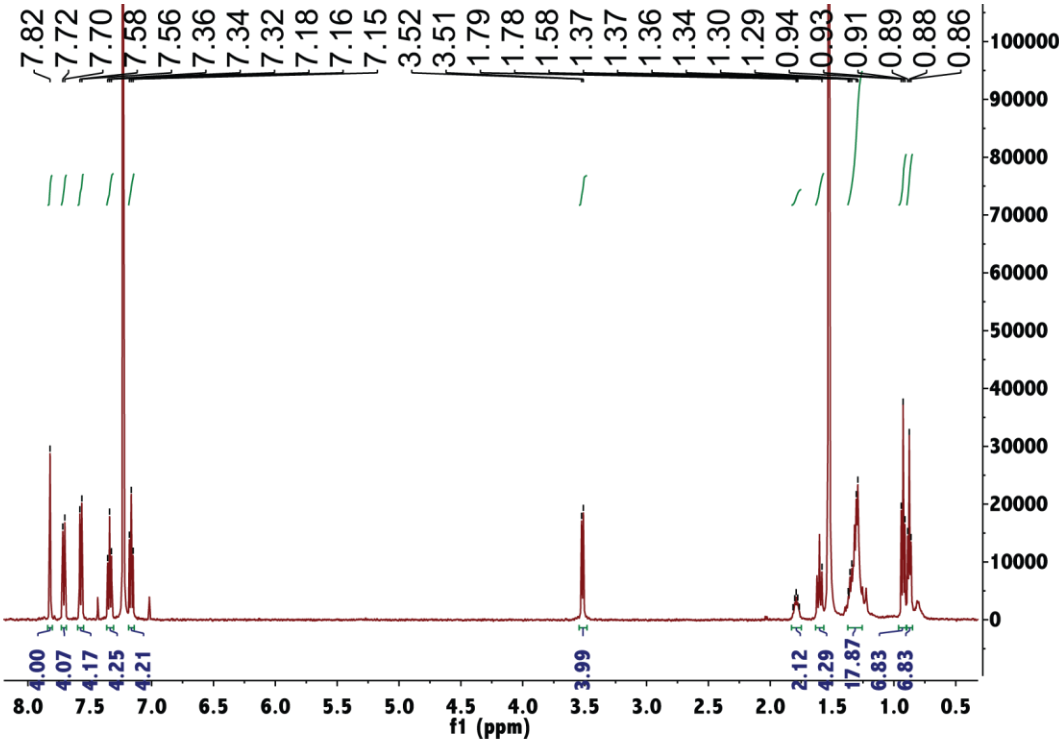

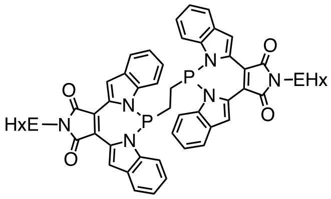

Figure S68. ^{1}H NMR spectrum of **Di-MI-P** in $CDCl_3$.

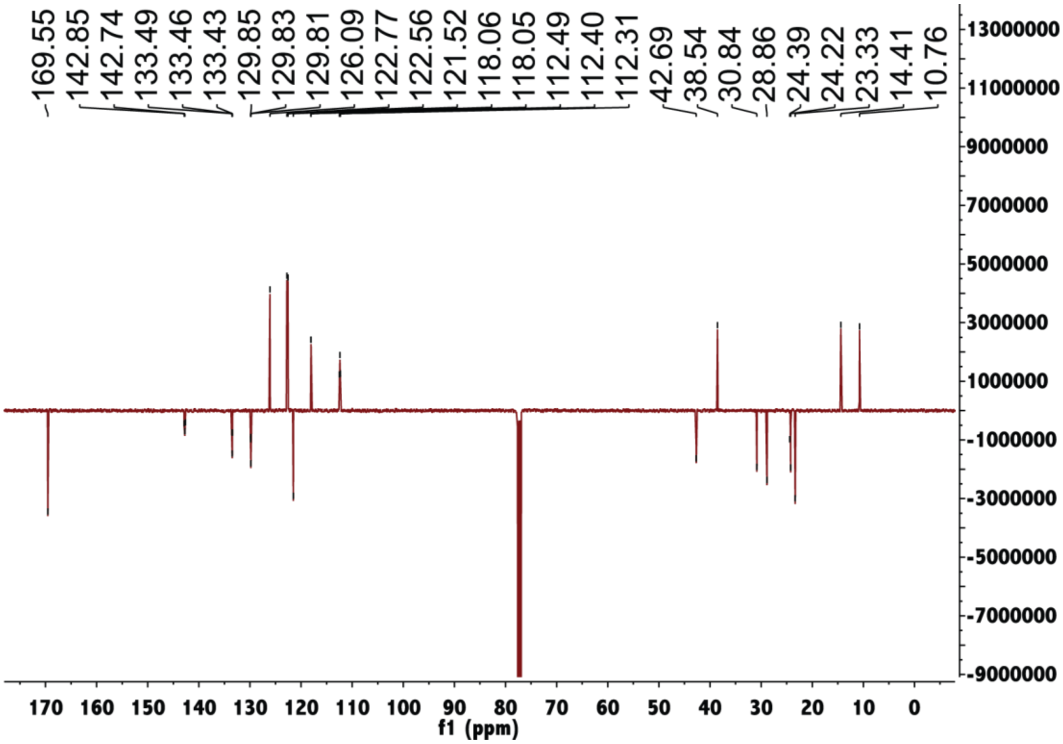

Figure S69. ^{13}C NMR spectrum of **Di-MI-P** in $CDCl_3$.

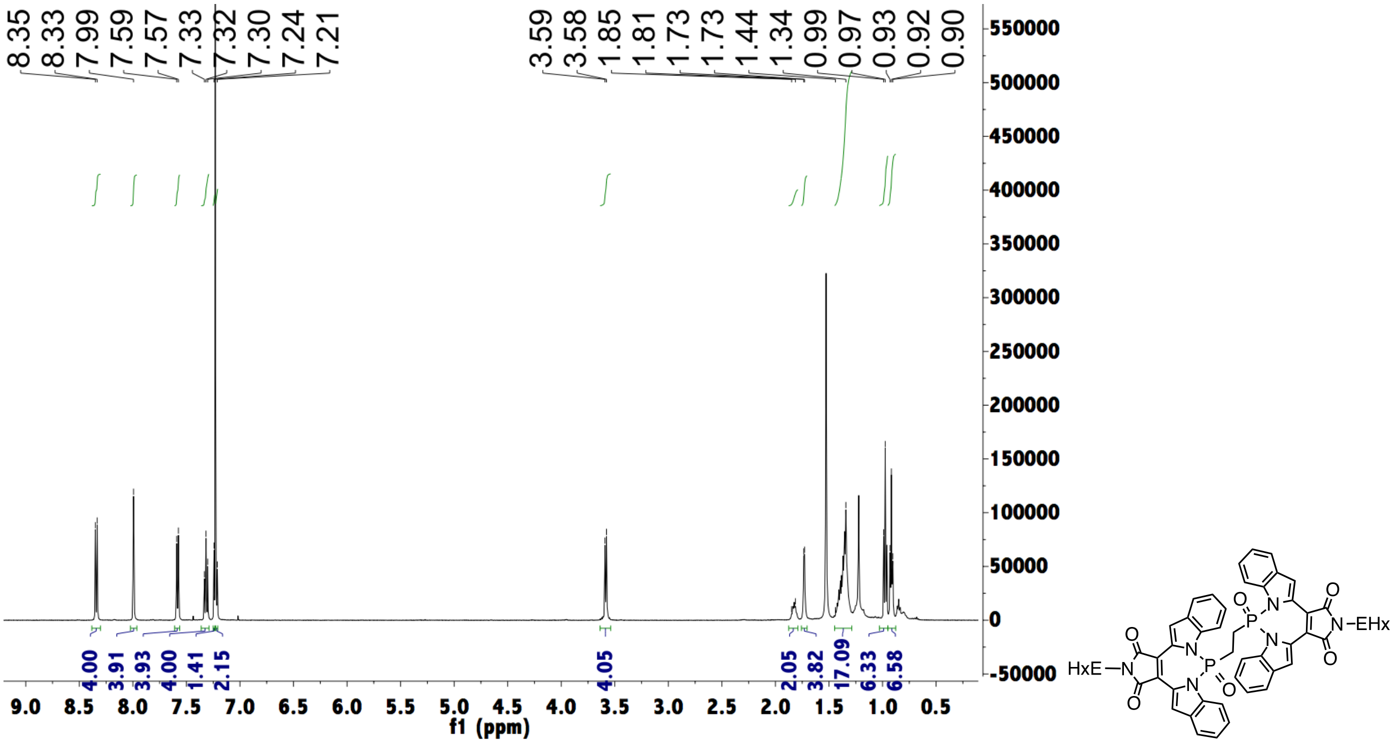

Figure S70. ^{1}H NMR spectrum of **Di-MI-PO** in $CDCl_3$.

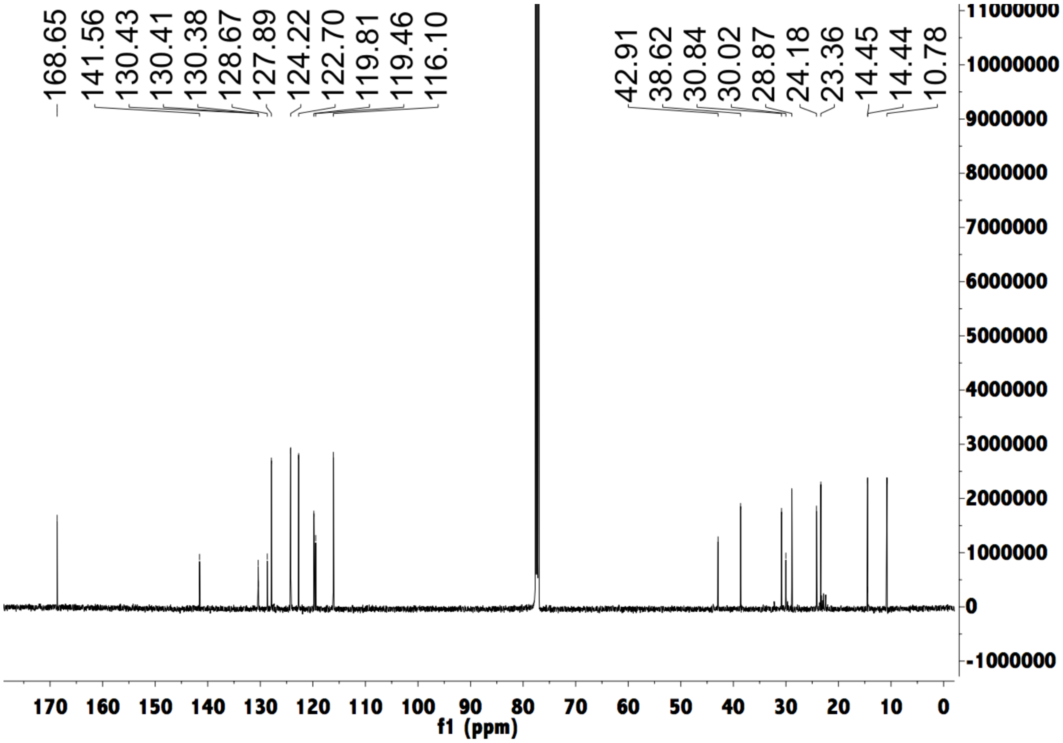

Figure S71. ^{13}C NMR spectrum of **Di-MI-PO** in $CDCl_3$.

Reference:

S1. M. J. Frisch, G. W. Trucks, H. B. Schlegel, G. E. Scuseria, M. A. Robb, J. R. Cheeseman, J. A., Jr., Montgomery, T. Vreven, K. N. Kudin, J. C. Burant, J. M. Millam, S. S. Iyengar, J. Tomasi, V. Barone, B. Mennucci, M. Cossi, G. Scalmani, N. Rega, G. A. Petersson, H. Nakatsuji, M. Hada, M. Ehara, K. Toyota, R. Fukuda, J. Hasegawa, M. Ishida, T. Nakajima, Y. Honda, O. Kitao, H. Nakai, M. Klene, X. Li, J. E. Knox, H. P. Hratchian, J. B. Cross, V. Bakken, C. Adamo, J. Jaramillo, R. Gomperts, R. E. Stratmann, O. Yazyev, A. J. Austin, R. Cammi, C. Pomelli, J. W. Ochterski, P. Y. Ayala, K. Morokuma, G. A. Voth, P. Salvador, J. J. Dannenberg, V. G. Zakrzewski, S. Dapprich, A. D. Daniels, M. C. Strain, O. Farkas, D. K. Malick, A. D. Rabuck, K. Raghavachari, J. B. Foresman, J. V. Ortiz, Q. Cui, A. G. Baboul, S. Clifford, J. Cioslowski, B. B. Stefanov, G. Liu, A. Liashenko, P. Piskorz, I. Komaromi, R. L. Martin, D. J. Fox, T. Keith, M. A. Al-Laham, C. Y. Peng, A. Nanayakkara, M. Challacombe, P. M. W. Gill, B. Johnson, W. Chen, M. W. Wong, C. Gonzalez, J. A. Pople, Gaussian 03, revision E.01; Gaussian Inc.: Wallingford, CT, 2007.

S2. Y. Ren. A. Orthaber, R. Pietschnig, T, Baumgartner, *Dalton Trans.* **2013**, *42*, 5314-5321.

S3. A. N. Bartynsk, M. Gruber, S. Das, S. Rangan, S. Mollinger, C. Trinh, S. E. Bradforth, K. Vandewal, A. Salleo, R. A. Bartynski, W. Bruetting, M. E. Thompson, *J. Am. Chem. Soc.* **2015**, *137*,5397-5405.

S4. C. Trinh, K. Kirlikovali, S. Das, M. E. Ener, H. B. Gray, Djurovich, P.; Bradforth, S. E.; Thompson, M. E. *J. Phys. Chem. C,* **2014**, *118*, 21834-21845.
